# Ecological patterns of the gut mycobiome and microbiome in ulcerative colitis across life stages

**DOI:** 10.3389/fcimb.2026.1769892

**Published:** 2026-04-28

**Authors:** Francesca Toto, Sabrina Cardile, Matteo Scanu, Valeria Marzano, Valentina Petito, Letizia Masi, Pierluigi Puca, Valentina Giorgio, Tommaso Alterio, Antonella Diamanti, Paola De Angelis, Loris Riccardo Lopetuso, Franco Scaldaferri, Lorenza Putignani, Federica Del Chierico

**Affiliations:** 1 Unit of Microbiome, Bambino Gesù Children’s Hospital, IRCCS, Rome, Italy; 2 Digestive Diseases and Nutritional Rehabilitation Clinical Unit, Nutritional Treatments in Complex Diseases Research Unit, Bambino Gesù Children’s Hospital, IRCCS, Rome, Italy; 3 CeMAD Translational Research Laboratories, Centro Malattie Apparato Digerente, Dipartimento di Scienze Mediche e Chirurgiche, Fondazione Policlinico Universitario “A. Gemelli”, IRCCS, Rome, Italy; 4 Dipartimento di Medicina Traslazionale e Chirurgia, Università Cattolica del Sacro Cuore, Rome, Italy; 5 IBD unit, Centro Malattie Apparato Digerente, Dipartimento di Scienze Mediche e Chirurgiche, Fondazione Policlinico Universitario “A. Gemelli”, IRCCS, Rome, Italy; 6 UOSD Spina Bifida e Altre Branche Specialistiche, Department of Woman and Child Health and Public Health, Fondazione Policlinico Universitario “A. Gemelli” IRCCS, Università Cattolica del Sacro Cuore, Rome, Italy; 7 Hepatology and Liver transplantation Unit, Bambino Gesù Children’s Hospital, IRCCS, Rome, Italy; 8 Gastroenterology, Digestive Endoscopy and Nutrition Unit, Bambino Gesù Children’s Hospital, IRCCS, Rome, Italy; 9 Department of Life Science, Health, and Health Professions, Link Campus University, Rome, Italy; 10 Unit of Microbiomics and Unit of Microbiome, Bambino Gesù Children’s Hospital, IRCCS, Rome, Italy

**Keywords:** Faecalibacterium prausnitzii, gut microbiota, gut mycobiota, inflammatory bowel diseases (IBD), intestinal dysbiosis, lysozyme, SIgA, ulcerative colitis

## Abstract

**Introduction:**

Age-related variations in the gut microbial communities may influence immune regulation and inflammatory processes in inflammatory bowel diseases (IBD). However, distinguishing age effects from differences in clinical characteristics remains challenging.

**Methods:**

We investigated life-stage–associated patterns of the gut microbiome and mycobiome while accounting for clinical heterogeneity between paediatric and adult ulcerative colitis (UC) populations. We analysed 73 targeted metagenomes of bacteria and 69 targeted metagenomes of fungi from 26 paediatric and 47 adult patients with UC. Microbial diversity metrics and multivariate analyses were applied to evaluate community variation, and mucosal immune markers were assessed by ELISA. Clinical variables, including disease activity, duration, and treatment exposure, were considered when interpreting age-related microbial differences.

**Results:**

Fungal communities exhibited higher richness in adults and formed distinct age-related clusters in beta-diversity analyses, whereas bacterial composition remained largely comparable across age groups. Children were enriched in inflammation-associated fungi (Saccharomycetes, *Aureobasidium*, *Cladosporium*) and depleted in taxa commonly linked to gut health (*Clavispora*, *Vishniacozyma*, *Betamyces*). Stratification by life stage identified young adults as displaying the most pronounced dysbiosis, characterised by Basidiomycota/Ascomycota and Firmicutes/Bacteroidota ratios, and reduced *Faecalibacterium prausnitzii* abundance. Age-associated immune patterns were observed, with lysozyme levels increasing across life stages, correlating with sIgA, and positively associating with *F. prausnitzii*, although declining with increasing disease severity.

**Discussion:**

Age-related variation was more evident in fungal than bacterial communities, suggesting that host developmental and immunological factors contribute to mycobiome configuration beyond clinical imbalance alone. Together, these findings indicate that life stage is linked to ecological variation of the gut mycobiome and mucosal immune responses in UC, while bacterial communities appear primarily shaped by disease-related factors. The transition from childhood to adulthood may represent a critical window of host–fungal interaction relevant for age-tailored microbiome-based strategies.

## 1 Introduction

Inflammatory bowel disease (IBD) is a chronic inflammatory condition of the gastrointestinal tract that includes Crohn’s disease (CD) and ulcerative colitis (UC). Although its exact aetiology remains unknown, immune dysregulation, environmental exposures and genetic susceptibility are recognized as major contributing factors (Guan, 2019; Maaser et al., 2019; Muzammil et al., 2023).

Age at diagnosis plays a crucial role in shaping the natural history of UC, influencing disease severity, clinical course, and treatment requirements (Gonçalves et al., 2015). Late-onset UC typically follows a milder trajectory, whereas early-onset disease is more frequently associated with steroid dependency or refractoriness (Tragnone et al., 1996), chronic intermittent symptoms (Azevedo et al., 2010), an increased risk of hospitalisation and greater use of systemic steroids and immunomodulators (Cosnes et al., 2011; Molodecky et al., 2012).

Beyond immunological factors and the influence of age on disease behaviour, an essential component of IBD pathophysiology is the intricate microbial ecosystem of the gut. The human gut harbours a complex community of bacteria, viruses, fungi, and other microorganisms that collectively maintain intestinal homeostasis (Hou et al., 2022). Fungi form an integral part of this ecosystem, shaping immune responses and interacting closely with bacterial communities (Huang et al., 2024). Dysbiosis—an imbalance within the bacteriome and mycobiome—is a recognised hallmark of IBD (Houshyar et al., 2021; Ha et al., 2024; Yadav et al., 2024; Kushkevych et al., 2025).

Fungal dysbiosis in IBD is characterised by alterations in fungal load and diversity including increased levels of pathogenic fungi such as *Candida* and *Malassezia* and reduced abundance of beneficial fungi such as *Saccharomyces cerevisiae* (Lam et al., 2019; Wang et al., 2021; Jena and Dutta, 2024). These mycobiome changes can exacerbate intestinal inflammation and contribute to the onset and progression of IBD (Mahmoudi et al., 2021; Wang et al., 2021). Moreover, fungal dysbiosis can influence bacterial community composition amplifying gut microbial imbalance (Lam et al., 2019; Balderramo et al., 2023; Yu et al., 2023). Interactions between fungi and the immune system—particularly through Dectin-1 signalling—play a critical role in modulating inflammatory responses (Li et al., 2024), whereas genetic factors, such as caspase recruitment domain family member 9 (CARD9) polymorphisms, further influence susceptibility to fungal colonisation and inflammation (Lam et al., 2019).

Age-related alterations in the gut microbiota can also occur, leading to chronic, low-grade inflammation known as “inflammaging”, which is associated with multiple age-related diseases (Thevaranjan et al., 2017; Santoro et al., 2020; D’Amico et al., 2024). Interactions between the gut mycobiome and the immune system may further influence the aging process and disease susceptibility (Al Bataineh et al., 2022; Shuai et al., 2022). Current therapeutic strategies aimed at correcting fungal dysbiosis include antifungal agents and probiotics containing beneficial fungi, such as *Saccharomyces boulardii* (Lam et al., 2019). Dietary interventions can also modulate the fungal community; for instance, high-carbohydrate diets have been shown to increase *Candida* abundance, whereas protein-rich diets have the opposite effect (Buttar et al., 2024).

Despite extensive research over the past decade using gut microbiome profiling and 16S rRNA sequencing to support IBD diagnosis and predict treatment escalation, the predictive accuracy achieved remains insufficient (Aldars-García et al., 2021).

Further investigations into etiological and prognostic factors are therefore needed to enhance diagnostic precision and enable more personalised treatment strategies.

In this study, we analysed the gut bacterial and fungal microbiota in paediatric and adult patients with UC to investigate life stage–associated ecological variation in gut dysbiosis.

## 2 Materials and methods

### 2.1 Sample collection

The adult population described in this study was derived from a subgroup of patients enrolled in the Division of Internal Medicine and Gastroenterology at the Policlinico Universitario A. Gemelli IRCCS Hospital in Rome, Italy, as described in Scanu et al. (2024b). The paediatric population was derived from a subgroup of patients enrolled at Bambino Gesù Children’s Hospital (OPBG) in Rome, Italy, and described in Del Chierico et al. (2024) (Del Chierico et al., 2024). UC diagnosis was established based on clinical, endoscopic, histological, and radiological criteria according to the Porto criteria for paediatric patients and standard international guidelines for adults. Exclusion criteria included antibiotic or probiotic use within 4 weeks prior to enrollment and the presence of infectious, ischemic, or other significant comorbid conditions. Clinical data collected at enrolment included age, sex, body mass index (BMI), disease duration, clinical activity scores, and ongoing therapies.

A single faecal sample was collected from each patient and stored at −80 °C at the OPBG Microbiota Biobank until further processing.

For bacterial metagenomic profiling, total DNA was extracted from 200 mg of stool samples using the QIAamp Fast DNA Stool Mini Kit (Qiagen, Hilden, Germany), following the manufacturer’s protocol. The V3–V4 hypervariable regions of the 16S rRNA gene were amplified using primer pairs specified in the Illumina MiSeq 16S Metagenomic Sequencing Library Preparation protocol (Illumina, San Diego, CA, USA). Fungal metagenomic analysis included a preliminary lysis step in which 200 mg of stool were resuspended in 500 μl of lysis buffer (50 mM Tris [pH 7.5], 10 mM EDTA, 28 mM 2-mercaptoethanol, and 10 U/ml lyticase) and incubated at 37 °C for 30 minutes under agitation. DNA extraction was carried out using the same procedure described for bacterial analysis. The fungal ITS2 region (~350 bp) was amplified using previously described primers (Lemoinne et al., 2020; Del Chierico et al., 2024). PCR amplification was performed with an initial denaturation at 94 °C for 2 minutes, followed by 35 cycles of 94 °C for 15 seconds, 52 °C for 30 seconds, and 72 °C for 45 seconds. Amplicons were purified using AMPure XP beads (Beckman Coulter, Brea, CA, USA). A second PCR step was then performed using Illumina adapter-linked ITS2 primers under the same thermal conditions for 15 cycles. After an additional purification step, libraries were indexed using the Nextera XT Index Kit (Illumina, San Diego, CA, USA). Final bacterial and fungal libraries were quantified using the Quant-iT PicoGreen dsDNA Assay Kit (Thermo Fisher Scientific, Waltham, MA, USA), pooled, and sequenced on the Illumina MiSeq platform according to the manufacturer’s instructions. Negative and positive controls were included in all amplification steps to monitor potential contamination and ensure data reliability for both 16S rRNA and ITS2 sequencing workflows. All samples from both cohorts were processed using an identical experimental workflow and sequencing platform.

### 2.2 16S rRNA and ITS2 data analyses

Bioinformatics analysis was conducted using the qiime2-amplicon-2024.5 pipeline (Bolyen et al., 2019). The DADA2 plugin (Callahan et al., 2016) was used for quality control, which included trimming the forward and reverse reads, denoising, removing chimeras, and merging the paired-end sequences. Representative sequences for each amplicon sequence variant (ASV) were taxonomically classified using the Greengenes2 plugin (McDonald et al., 2024) against the Greengenes reference database v2022.2 (http://ftp.microbio.me/greengenes_release/current/) for bacterial sequences, and using the naive Bayes classifier against the UNITE ITS dynamic database v10 (https://unite.ut.ee) for fungal sequences (Abarenkov et al., 2024).

All sequencing datasets were reprocessed using a standardized bioinformatic pipeline to minimize technical variability and batch effects to minimize technical variability (Scanu et al., 2024a).

The bacterial and fungal ASV matrices were imported into R software v4.4.1 using the qiime2R package v0.99.6.

Prior to alpha- and beta-diversity analyses, ASV matrices were normalised using the rarefaction method based on the minimum sequencing depth across samples. Alpha-diversity indices were compared using the non-parametric Mann–Whitney U test, while beta-diversity differences were assessed using PERMANOVA.

For the univariate and multivariate analyses, the ASV tables were normalized using the Cumulative Sum Scaling (CSS) method (Paulson et al., 2013). Taxa with a relative abundance below 0.01% and present in fewer than 25% of the samples in the smallest comparison group were excluded from subsequent analyses. Univariate analysis was performed using the Linear Discriminant Analysis Effect Size (LEfSe) method (Segata et al., 2011), with a significance threshold of α = 0.05 and a log LDA score > 3.0, in order to identify differentially abundant taxa. Multivariate analysis was performed using Partial Least Squares Discriminant Analysis (PLS-DA) with the mixOmics R package v6.28 and Principal Component Analysis (PCA) with the ropls R package v1.36 from Bioconductor.

Associations between microbial genera and host variables were assessed using MaAsLin2 (Mallick et al., 2021), including categorical age, Mayo endoscopic score (disease activity), BMI, disease duration, and treatment exposure as covariates. P-values were corrected using the Benjamini–Hochberg procedure, and associations with q-values < 0.1 were considered statistically significant.

Supervised machine-learning models, including Random Forest (RF), Gradient Boosting Machine (GBM), and linear Support Vector Machine (SVM), were trained using microbial abundance profiles derived from both bacterial and fungal ASV matrices as input features. Model performance was assessed using an outer repeated stratified k-fold cross-validation procedure, in which hyperparameter tuning was performed within an inner stratified k-fold cross-validation loop on the corresponding training data. Performance metrics included Accuracy, Precision, Recall, F1-score, and ROC-AUC, with AUC computed using the predicted probability of the positive class. Out-of-fold predictions were aggregated across all outer resamples to estimate pooled ROC-AUC values for each model.

Inter-kingdom correlation analyses were performed using the Spearman’s correlation method. Significant correlations were visualized using network graphs generated with the igraph and ggraph R packages (v2.1.1 and v2.2.1, respectively).

To predict metagenome functional content from 16S rRNA gene surveys, the PICRUSt (Phylogenetic Investigation of Communities by Reconstruction of Unobserved States) was applied and an unpaired Linear discriminant analysis (LDA) was used for statistical analysis (Langille et al., 2013). Only pathways with p < 0.05 and an LDA score > 3 were considered as significantly altered.

The raw sequences of 73 bacterial metagenomic profiles (PRJNA280490; PRJNA996768) and 69 fungal metagenomic profiles (PRJNA996917; PRJNA935155) are available in the NCBI database (https://www.ncbi.nlm.nih.gov/).

### 2.3 Real-Time PCR quantitative analysis of *F. prausnitzii*

We extracted DNA from 200 mg of stool following the instructions provided with the QIAamp PowerFecal Pro DNA Kit (Qiagen, Hilden, Germany).

The RIDA^®^GENE Faecalibacterium prausnitzii kit (R-Biopharm AG, Darmstadt, Germany) was used to qualitatively and quantitatively detect F. prausnitzii DNA in stool samples using a multiplex real-time PCR (LightCycler 480II, Roche Diagnostics, Mannheim, Germany), following the manufacturer’s instructions. The F. prausnitzii qPCR data, representing gene copies per reaction, were converted into absolute copy numbers (Cp) per gram of stool using the following formula:


$$\text{Cp}/\text{gr stool}=\frac{\text{copies reaction}*\text{elution volume of extracted DNA}}{\text{volume of DNA used}*\text{gr of stool sample used in DNA extraction}}$$

### 2.4 Normalization for Linear chart

To allow comparison between variables with different scales in line charts, values were normalized to the [0,1] range using the following formula:


$$Xnorm=\frac{X-Xmin}{Xmax-Xmin}$$

where Xmin and Xmax represent the minimum and maximum values of the variable, respectively. This normalization was applied exclusively for graphical representation and did not affect statistical analyses.

### 2.5 Immunity and inflammation of mucosa

Faecal secretory immunoglobulin A (sIgA) and faecal lysozyme levels were measured using enzyme-linked immunosorbent assay (ELISA) kits (Immundiagnostik AG, Bensheim, Germany) according to the manufacturer’s instructions. Absorbance was measured at 450 nm using a microplate reader for both tests.

## 3 Results

### 3.1 Patients’ characteristics

The paediatric UC cohort included 26 children with a median age of 11.2 years (± 4.8). The adult UC cohort included 47 patients with a median age of 40.9 (± 14.7) years (**Table 1**). Gender distribution was comparable between groups (paediatric F:M 13:13; adult F:M 23:24). Paediatric patients had a lower mean BMI than adults (19.2 ± 4.1 vs 23.8 ± 2.1 kg/m²) and a shorter disease duration (4.4 ± 2.9 vs 9.3 ± 7.5 years).

**Table 1:** Distribution of demographical and clinical characteristics of the cohorts.

Features		Paediatric population (n=26)	Adult population (n=47)	p value
Gender (F:M)		13:13	23:24	–
Age (years) mean ± SD		11.2 ± 4.8	40.9 ± 14.7	0.000^1^
BMI (Kg/m^2^) mean ± SD		19.2 ± 4.1	23.8 ± 2.1	0.000^1^
Disease duration (years) mean ± SD		4.4 ± 2.9	9.3 ± 7.5	0.008^1^
Mayo Endoscopic (%)	Remission	38	2	0.000^2^
	Mild activity	23	2	
	Moderate activity	19	50	
	Severe activity	20	46	
Steroids (%)		43	73	0.023^2^
Immunomodulators (%)		85	43	0.010^2^
Biologic therapy (%)		35	70	0.006^2^
5-ASA therapy (%)		89	64	0.029^2^

^1^ T test p value, ^2^Fisher’s Test p value.

As shown in **Table 1**, 39% of the paediatric population and 96% of the adult population reported moderate-to-severe UC activity, characterized by marked erythema, loss of vascular pattern, friability, erosions, spontaneous bleeding, ulceration and, severe mucosal damage.

Furthermore, 73% and 96% of the paediatric and adult populations, respectively, reported a moderate-to-severe grade of UC, with diarrhea, as well as mild anaemia, fever, tachycardia, and elevated erythrocyte sedimentation rate (ESR) and C-reactive protein (CRP) levels. Regarding treatment exposure, steroid use was more frequent among adults than paediatric patients (73% vs 43%). Immunomodulator therapy was more common in the paediatric population (85% vs 43%), while biologic therapy was more frequently administered in adults (70% vs 35%). Use of 5-aminosalicylic acid (5-ASA) therapy was higher in paediatric patients compared with adults (89% vs 64%).

### 3.2 Gut mycobiota shows stronger age-associated variation than bacterial microbiota in UC cohorts

We analysed gut microbiota profiles to explore differences between paediatric and adult patients with UC. Bacterial profiling yielded 8,154 ASVs, which were filtered and assigned to 353 bacterial genera. No significant differences were observed in alpha-diversity indices between the two cohorts (**Supplementary Figures 1A, B**). For both the Shannon and Simpson indices, effect sizes were negligible (partial η² = 0.008 and 0.002, respectively), indicating that age group accounted for less than 1% of the variance in bacterial diversity.

Beta-diversity analysis similarly revealed no significant separation between paediatric and adult samples (PERMANOVA p=0.44, R²=0.014; **Supplementary Figure 1C**). At the genus level, only minor compositional differences were observed between paediatric and adult UC patients (**Supplementary Figure 1D**).

Fungal profiling yielded 1,075 ASVs, which were filtered and grouped into 149 fungal genera. In contrast to bacterial communities, gut mycobiota analysis showed significantly higher alpha-diversity values in paediatric patients compared with adults (p ≤ 0.05; **Supplementary Figure 1E** and S1F). Effect size estimates indicated a moderate-to-large contribution of age group to fungal diversity variation (partial η²=0.167 and 0.754 for the Shannon and Simpson indices, respectively).

Beta-diversity analysis confirmed significant differences between paediatric and adult cohorts (PERMANOVA p=0.001, R²=0.142; **Supplementary Figure 1G**). Paediatric samples formed a relatively compact cluster, whereas adult samples were distributed across three sub-clusters, suggesting greater compositional heterogeneity within the adult cohort.

At the genus level, distinct differences in fungal community composition were observed between paediatric and adult UC patients (**Supplementary Figure 1H**).

**Figure 1:**
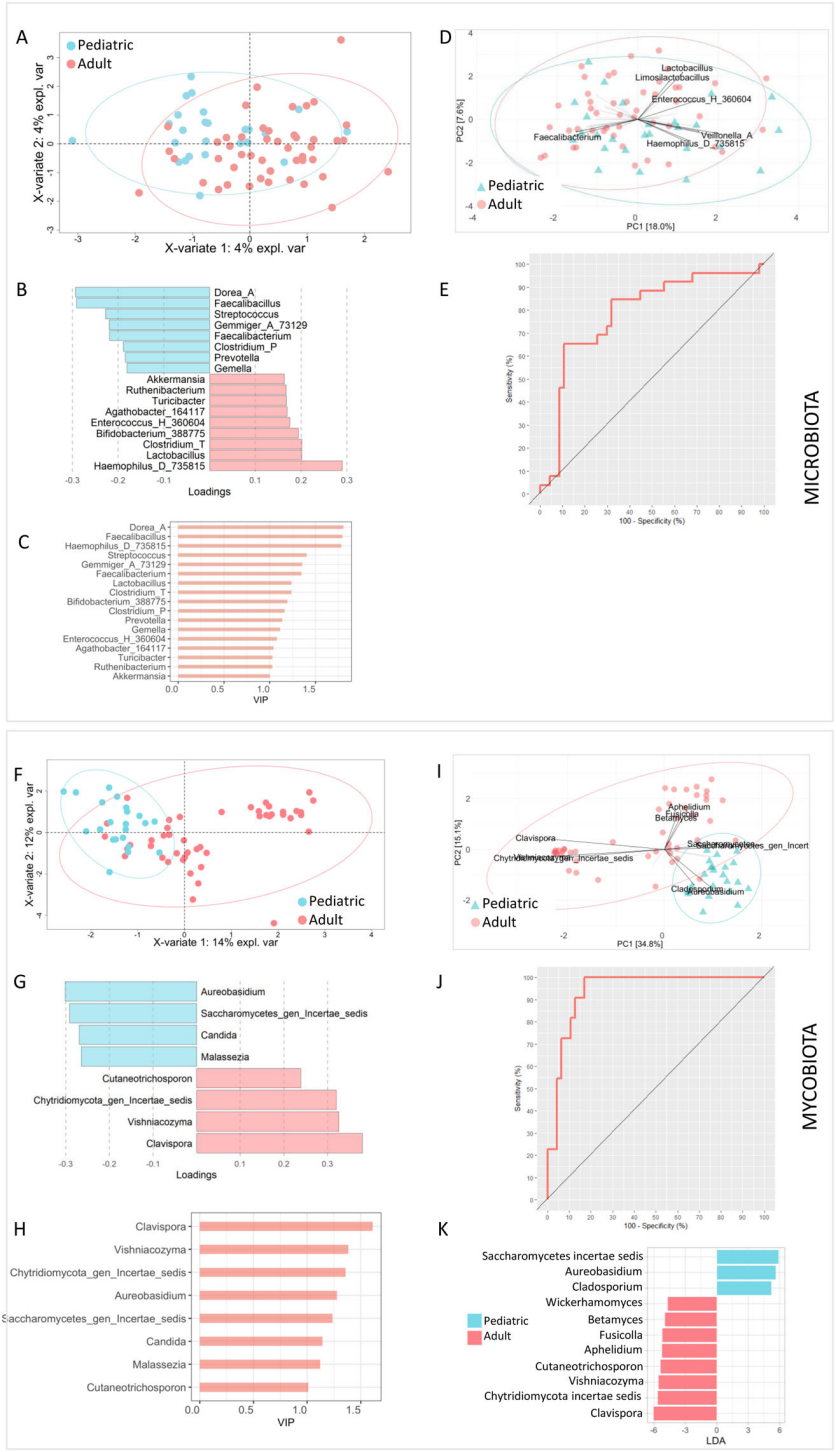
Comparison of bacterial and fungal gut microbiota profiles between paediatric and adult ulcerative colitis (UC) patients. **(A–D)** Genus-level bacterial community analyses comparing paediatric and adult UC patients. Partial least squares discriminant analysis (PLS-DA) **(A)** and principal component analysis (PCA) **(D)** showed no clear separation between the two patient groups, with samples widely distributed across ordination space. The loading variables plot and the variable importance in projection (VIP) scores from PLS-DA are shown in panels B and C, respectively. Receiver operating characteristic (ROC) analysis confirmed the limited discriminatory performance of the bacterial model (RMSE = 0.315; R² = 0.578, p = 0.4; Q² = 0.184, p = 0.02; AUROC = 0.7848) **(E–J)** Genus-level fungal community analyses comparing paediatric and adult UC patients. In contrast to bacteria, both PLS-DA **(F)** and PCA **(I)** revealed distinct clustering between the two groups. Corresponding loading variables and VIP score plots are presented in panels **(G, H)**. ROC analysis demonstrated strong model performance in discriminating paediatric and adult patients based on mycobiome composition (RMSE = 0.168; R² = 0.876, p = 0.02; Q² = 0.655, p = 0.02; AUROC = 0.9381) **(J, K)** Linear discriminant analysis effect size (LEfSe) identified fungal taxa differentially enriched between paediatric and adult UC patients.

Both multivariate PLS-DA and PCA confirmed the absence of differences between the gut microbial profiles of the two groups (**Figure 1**, panels A-D). ROC curve revealed the model’s poor ability to distinguish between the two classes (RMSE = 0.315; R^2^ = 0.578, p value=0.4; Q^2^ = 0.184, p value= 0.02; AUROC = 0.7848) (**Figure 1E**). LEfSe confirmed that there were no differences between the paediatric and adult groups. In fact, no bacterial markers were found to be associated with age.

The multivariate statistical approach applied to the mycobiota results confirmed marked differences between the two groups. Both PLS-DA and PCA showed distinct clusters for the paediatric and adult groups (**Figure 1**, panels F-H). Both analyses showed Saccharomycetes, *Aureobasidium*as characteristic of the paediatric group, while genera *Cutaneotrichosporon*, *Vishniacozyma*, Chytridiomycota and *Clavispora* characteristic of the adult group. ROC curve revealed model’s ability to distinguish between the two groups (RMSE = 0.168; R^2^ = 0.876, p value=0.02; Q^2^ = 0.655, p value= 0.02; AUROC = 0.9381) (**Figure 1J**).

Fungal biomarkers distinguishing paediatric and adult UC patients were identified using LEfSe analysis (**Figure 1K**). Taxa enriched in the paediatric cohort included the class Saccharomycetes and the genera *Aureobasidium* and *Cladosporium*. In contrast, *Wickerhamomyces*, *Betamyces*, *Fusicolla*, *Aphelidium*, *Cutaneotrichosporon*, *Vishniacozyma*, members of Chytridiomycota, and *Clavispora* were relatively more abundant in adult patients (**Figure 1K**).

To evaluate the potential influence of disease activity as a confounding factor, stratified analyses were performed comparing paediatric and adult patients within the same disease activity categories. Several fungal taxa—including *Cutaneotrichosporon*, *Clavispora*, *Cladosporium*, and *Aureobasidium*—remained significantly different between age groups across multiple activity stratification (**Supplementary Table 1**). These findings indicate that differences in fungal community composition persist after accounting for disease activity, supporting the presence of life-stage–associated variation in the gut mycobiome.

### 3.3 Multivariable associations between fungal taxa and host clinical variables

To address potential confounding by disease severity, Mayo endoscopic score was included as a covariate in the MaAsLin2 multivariable models together with BMI, disease duration, and treatment exposure. After adjustment, several fungal taxa remained significantly associated with categorical age, whereas no significant associations were observed between fungal taxa and Mayo score after multiple-testing correction. These findings indicate that disease activity did not independently explain the observed mycobiome variation, supporting the persistence of life-stage–associated differences beyond disease severity (**Supplementary Table 2**).

### 3.4 Integrated analysis of bacterial and fungal communities differentiates paediatric and adult UC cohorts

Machine learning was used to investigate whether models based on microbiota, mycobiota, or both could distinguish between paediatric and adult cohorts. Model performance was evaluated based on the area under the ROC curve (AUROC), which indicates the probability of correctly classifying a randomly selected patient to the correct study group.

**Figure 2:**
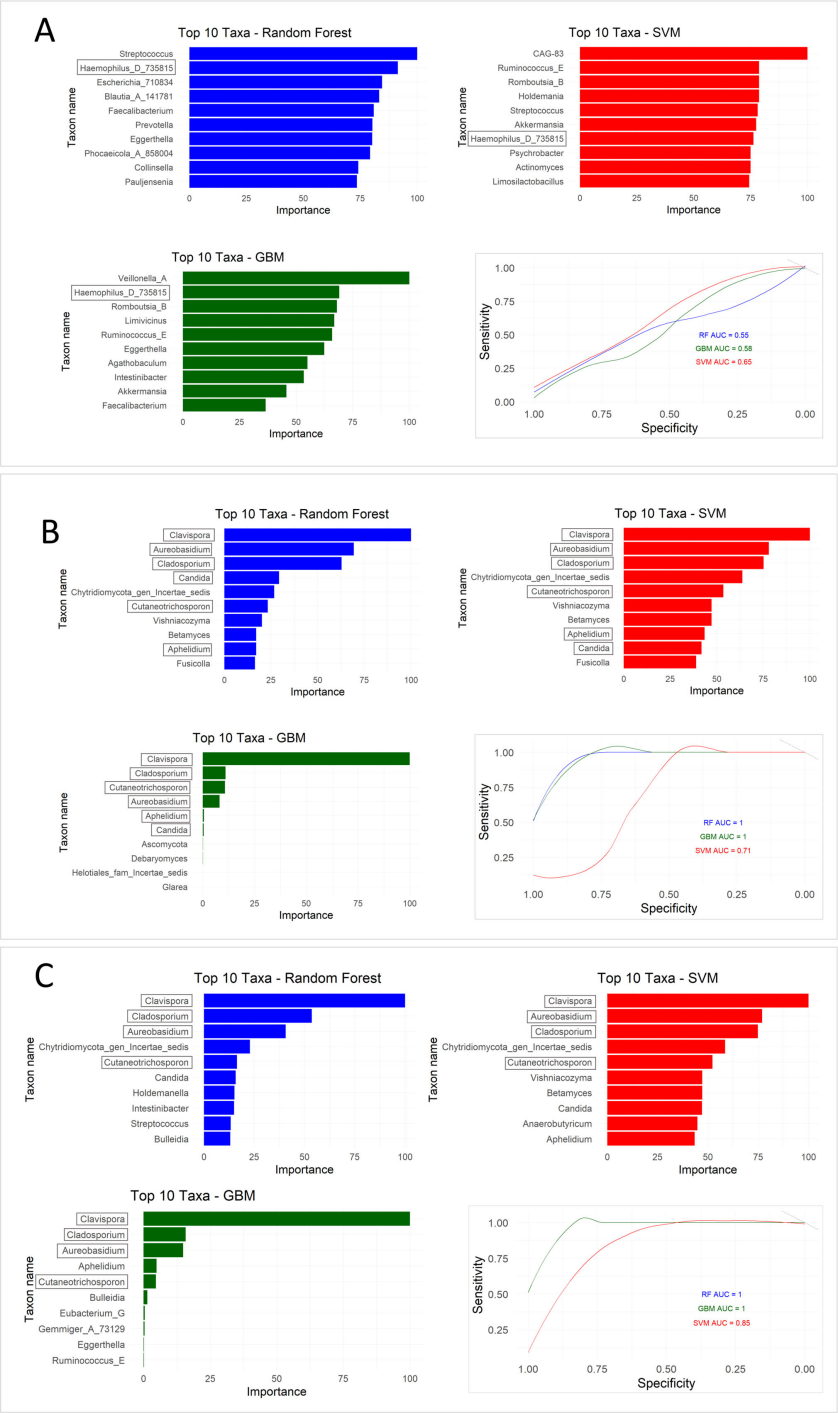
Machine learning classification performance based on bacterial microbiota, fungal mycobiota, and combined datasets in paediatric and adult ulcerative colitis (UC) patients. **(A)** Models trained on bacterial microbiota profiles showed limited discriminatory ability between paediatric and adult UC patients, with low classification performance across algorithms (Random Forest [RF] AUC = 0.55; Gradient Boosting Machine [GBM] AUC = 0.58; Support Vector Machine [SVM] AUC = 0.65). **(B)** Models based on mycobiota profiles demonstrated improved classification performance, particularly for RF and GBM models (RF AUC = 1.0; GBM AUC = 1.0; SVM AUC = 0.71). **(C)** Integration of bacterial and fungal datasets further enhanced model performance across all algorithms (RF AUC = 1.0; GBM AUC = 1.0; SVM AUC = 0.85). Model performance was evaluated using the area under the receiver operating characteristic curve (AUROC).

First, the AUROC for the microbiota data showed low performance values for all models (RF AUC = 0.55; GBM AUC = 0.58; SVM AUC = 0.65) (**Figure 2A**). This analysis revealed that *Haemophilus* D 735815 was the only shared classifier in all models.

Applying AUROC to mycobiota data showed a high value for RF and GBM (AUC = 1), and a low value for SVM (AUC = 0.71) (**Figure 2B**). *Clavispora*, *Aureobasidium*, *Cladosporium*, *Candida*, *Cutaneotrichosporon* and *Aphelidium* were shared classifiers in all three models.

Finally, applying the models to joint data (*i.e.*, mycobiota and microbiota) increased the performance of all models (RF AUC = 1; GBM AUC = 1; SVM AUC = 0.85) (**Figure 2C**).

These models identified *Clavispora*, *Cladosporium*, *Aureobasidium* and *Cutaneotrichosporon* as classifiers.

Overall, these results suggest that the fungal component of the microbiota is most effective at discriminating between paediatric and adult UC patients.

The cross-validation procedures confirmed the robustness of the machine-learning models, showing consistent performance across resampling iterations. Model validation metrics supported the reliability of the classification results obtained from microbial abundance profiles (**Supplementary Table 3**).

### 3.5 Differences in bacterial–fungal interaction networks across life stages in UC

**Figure 3:**
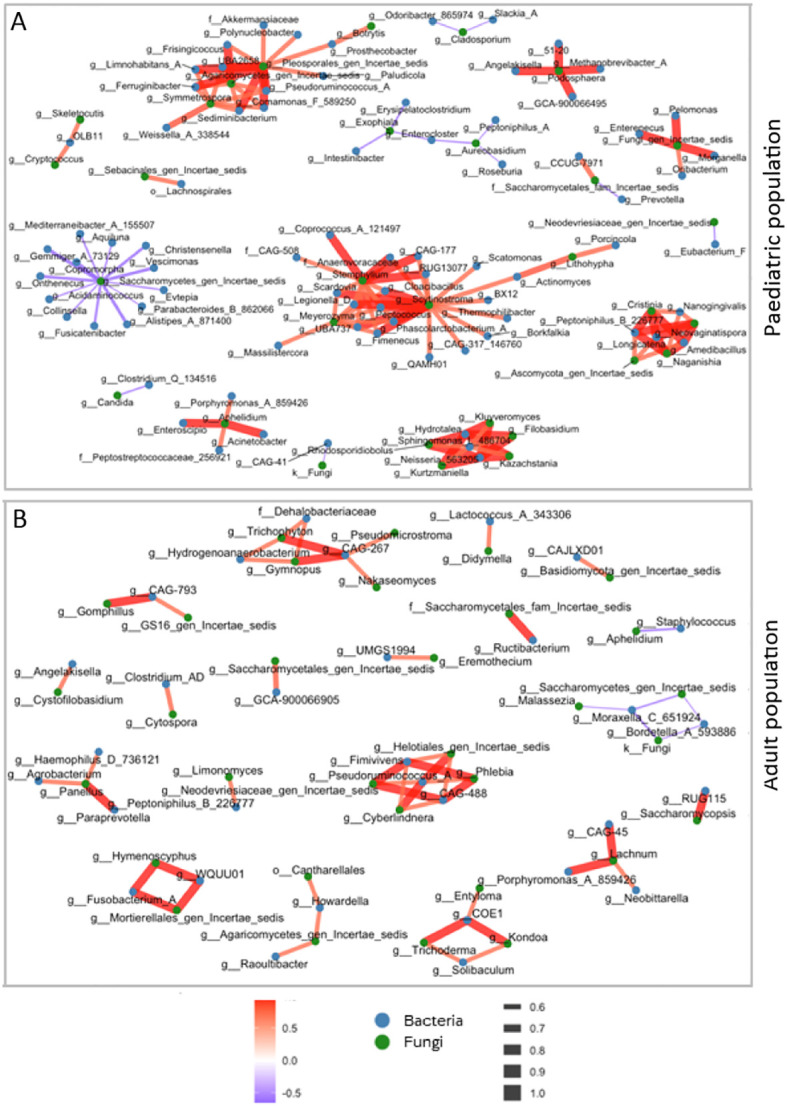
Inter-kingdom correlation networks in paediatric and adult ulcerative colitis (UC) patients. Bacterial–fungal inter-kingdom association networks reconstructed for the paediatric cohort **(A)** and the adult cohort **(B)**. Nodes represent bacterial taxa (blue circles) and fungal taxa (green circles). Edges indicate statistically significant pairwise associations identified using Spearman’s rank correlation analysis (p < 0.05). Red edges denote positive correlations, whereas violet edges denote negative correlations. Edge thickness is proportional to the absolute correlation coefficient (ρ), reflecting the strength of the association.

Spearman’s correlation analysis revealed a positive correlation between age and *Vishniacozyma* (ρ=0.626), *Cutaneotrichosporon* (ρ=0.588) and *Clavispora* (ρ=0.69). Conversely, a negative correlation was observed between age and *Cladosporium* (ρ=-0.516), *Aureobasidium* (ρ=-0.538) and Saccharomycetes (ρ=-0.394) (**Figure 3A**). Spearman’s correlation analysis confirmed the absence of any correlation between age and bacterial taxa.

Bacterial-fungal network analysis was performed in the paediatric and adult cohorts (**Figure 3B**). In the paediatric cohort, 16 networks were observed between fungi and bacteria, six of which were characterised by negative correlations. *Candida* was negatively correlated with *Clostridium* and Saccharomycetes_gen_incertae_sedis was negatively correlated with *Parabacteroides* B862066, *Alistipes*, *Fusicatenibacter*, *Collinsella*, *Acidaminococcus*, *Gemmiger*, and *Christensenella*. Furthermore, *Cladosporium* exhibited negative correlations with *Odoribacter* 865974 and *Slackia* A, and *Aureobasidium* showed negative correlations with *Peptoniphilus* A, *Roseburia* and *Enterocloster*. In the adult cohort, 18 networks were observed between fungi and bacteria, most of which were characterised by positive correlations (**Figure 3C**). For example, *Clostridium* was positively correlated with *Cytospora*, while *Staphylococcus* was negatively correlated with *Aphelidium*. Finally, *Moraxella* was negatively correlated with *Malassezia*, and both undefined fungi and Saccharomycetes, which were in turn negatively correlated with *Bordetella*.

### 3.6 Life-stage–associated variation in predicted functional pathways of the gut microbiota

To gain insight into the direct relationship between changes in the intestinal microbiota and changes in the metabolic profile in children and adults with UC, PICRUSt was used to predict potential functional pathways that could be affected by the gut microbiota. The following pathways were found to be increased in UC children: Lipopolysaccharide biosynthesis, Nitrogen metabolism, Ubiquinone and other terpenoid-quinone biosynthesis, Ascorbate and aldarate metabolism, Toluene degradation, Peroxisome Sulfur metabolism, Tropane, piperidine and pyridine alkaloid biosynthesis, Novobiocin biosynthesis, Phosphatidylinositol signaling system and Flavone and flavonol biosynthesis (**Supplementary Figure 2**).

The following pathways were found to be increased in UC adults: Epithelial cell signaling in Helicobacter pylori infection, Base excision repair, D-Alanine metabolism, Tetracycline biosynthesis, Staphylococcus aureus infection, Terpenoid backbone biosynthesis, Aminoacyl-tRNA biosynthesis, Peptidoglycan biosynthesis, RNA polymerase (**Supplementary Figure 2**).

### 3.7 Associations between life stage, disease activity, mucosal immunity, and gut dysbiosis

**Figure 4:**
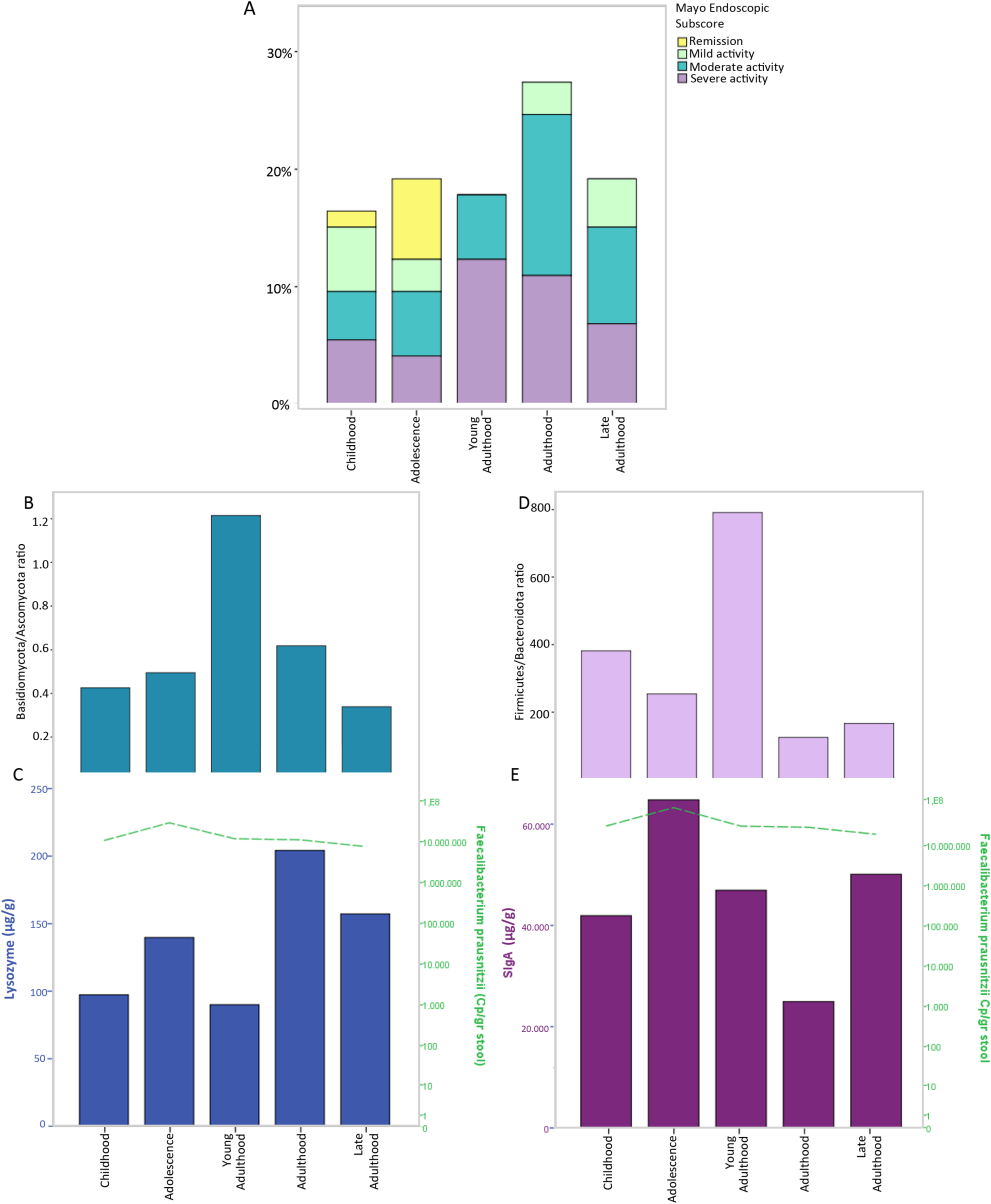
Distribution of disease activity, microbial ecological indices, and immune markers across age-defined subgroups in ulcerative colitis (UC) patients. Patients were stratified into five age-defined subgroups: Childhood (2–12 years), Adolescence (13–18 years), Young Adulthood (19–30 years), Adulthood (31–50 years), and Late Adulthood (51–70 years). Bar plots illustrate the distribution of patients according to Mayo endoscopic subscore **(A)**, together with Basidiomycota/Ascomycota **(B)** and Firmicutes/Bacteroidota **(C)** ratios. **(D, E)** report faecal levels of lysozyme and secretory IgA (sIgA), respectively and the linear trend of *Faecalibacterium prausnitzii* abundance.

To explore age-related patterns across the disease spectrum, we stratified the cohort into age-defined groups: childhood (n. 12, 2–12 years), adolescence (n. 14, 13–18 years), young adulthood (n. 13, 19–30 years), adulthood (n. 20, 31–50 years), late adulthood (n. 14, 51–70 years) (Erikson, 1993; Arnett, 2000). Descriptive analysis indicated that 69% of patients in the young adulthood group presented severe UC activity (**Figure 4A**). In addition, this group showed higher Basidiomycota/Ascomycota and Firmicutes/Bacteroidota ratios compared to the other age groups (**Figures 4B, D**, respectively). To further characterize the inflammatory profile across age groups, we measured faecal lysozyme and sIgA levels together with the absolute abundance of *F. prausnitzii* (**Figures 4C, E**, **Supplementary Figure 3A**).

Lysozyme levels showed an increasing trend with age (**Supplementary Figure 3A**), whereas sIgA levels remained relatively elevated from childhood through young adulthood, followed by a decline in adulthood (**Supplementary Figure 3B**). The abundance of *F. prausnitzii* exhibited a peak during adolescence and a progressive decrease in older age groups, with the lowest levels observed in late adulthood. When comparing paediatric and adult populations, we observed a statistically significant increase in sIgA levels in subjects and a decrease in *F. prausnitzii* abundance in adults (**Table 2**). However, comparisons across all age subgroups did not reveal statistically significant differences for most markers. Pairwise analyses identified a significant increase in IgA levels in adolescents compared with the adulthood group, as well as higher lysozyme levels in the adulthood group compared with childhood and young adulthood.

**Table 2:** Lysozyme, sIgA and *F. prausnitzii* faecal levels in paediatric and adult UC patients.

Biomarkers	Paediatric population (n=26)	Adult population (n=46)	p value	Childhood (n=12)	Adolescence (n=14)	Young Adulthood (n=13)	Adulthood (n=20)	Late Adulthood (n=14)	p value
Lysozyme (μg/g)	119.6± 11.7	155.7± 29.1	0.236	97.3± 18.7	139.7± 12.0	90.0± 21.2	204.1± 58.8	157.2± 45.3	0.208
sIgA (μg/g)	5.4*10^4^± 1.2*10^3^	3.8*10^4^± 6.0*10^3^	0.020	4.1*10^4^± 1.1*10^4^	6.4*10^4^± 2.2*10^4^	4.6*10^4^± 1.4*10^4^	2.4*10^4^± 6.2*10^3^	5.0*10^4^± 1.0*10^5^	0.288
*F. prausnitzii* (Cp/gr stool)	6.1*10^7^± 3.4*10^7^	2.4*10^7^± 8.5*10^6^	0.020	3.1*10^7^± 2.8*10^7^	8.8*10^7^± 5.9*10^7^	3.2*10^7^± 2.2*10^7^	2.6*10^7^± 1.3*10^7^	1.4*10^7^± 8.9*10^6^	0.470

Correlation analysis revealed positive association between lysozyme levels and *F. prausnitzii*, while *F. prausnitzii* was negatively correlated with the Mayo score. Additionally, sIgA levels were positively correlated with *Bacteroides* H (ρ=0.38, p value=0.01), CAG508 (ρ=0.32, p value=0.04), *Romboutsia* (ρ=0.31, p value=0.04) and *Aureobasidium* (ρ=0.32, p value=0.03). Lysozyme levels showed positive correlations with *Agathobacter* 164117 (ρ=0.33, p value=0.02), *Alistipes* A871400 (ρ=0.30, p value=0.04), *Bacteroides* H (ρ=0.32, p value=0.03), *Collinsella* (ρ=0.47, p value=0.00), *Phocaeicola* A858004 (ρ=0.44, p value=0.00) and *Roseburia* (ρ=0.32, p value=0.03).

Overall, these analyses provide an exploratory framework for understanding age-associated patterns across the disease spectrum, informing future hypothesis-driven studies in larger cohorts.

## 4 Discussion

It has been proposed that the phenotype and natural history of IBD vary according to age at disease onset (Gonçalves et al., 2015). This heterogeneity suggests that the respective contributions of genetics, the host immune system and environmental factors to the aetiology and phenotype of CD and UC differ across the lifespan (Ruel et al., 2014). Paediatric IBD, in particular, is typically characterised by a more aggressive disease course, extensive involvement and a greater overall burden than adult-onset disease (Kim and Ye, 2019), whereas adult IBD tends to show a more stable pattern over time. These differences indicate that IBD represents a spectrum of age-dependent conditions rather than a single, uniform disease. The observed therapeutic differences between paediatric and adult patients likely reflect age-specific management strategies in UC. Paediatric-onset disease is commonly managed with early steroid-sparing approaches aimed at preserving growth and preventing cumulative inflammatory burden, explaining the higher use of immunomodulators and 5-ASA therapy in children (Turner et al., 2018). Conversely, adult patients demonstrated greater corticosteroid and biologic exposure, which may be related to longer disease duration and treatment escalation over time (Harbord et al., 2017).

Although intestinal dysbiosis in UC has been well documented in cross-sectional comparisons between patients and healthy controls, the influence of age-related microbial changes remains largely unexplored (Putignani et al., 2016, Putignani et al., 2021; Del Chierico et al., 2024; Scanu et al., 2024b; Toto et al., 2024; Aldrian et al., 2025). Understanding how the gut microbiota and mycobiota evolve across ages may have important implications for disease pathogenesis and management. Identifying age-specific microbial signatures could in fact support the development of personalised therapeutic strategies.

In this study, one of the first to characterise both the intestinal mycobiome and microbiome of UC patients from childhood to adulthood, we observed distinct fungal profiles between paediatric and adult populations. The fungal community exhibited clear age-associated restructuring, with both alpha and beta diversity analyses indicating distinct ecological configurations between paediatric and adult patients. These findings suggest that the mycobiome may be particularly sensitive to age-related factors in UC, potentially reflecting differences in immune maturation, environmental exposures, or disease history. In particular, the paediatric UC group was notably enriched in Saccharomycetes, *Aureobasidium* and *Cladosporium*, and depleted in *Clavispora*, Chytridiomycota, *Vishniacozyma*, *Cutaneotrichosporon*, *Aphelidium*, *Fusicolla*, *Betamyces* and *Wickerhamomyces*.

Increased Saccharomycetes have previously been linked to greater UC severity, inflammation and heightened immune activation (Qiu et al., 2017; Hsia et al., 2023). *Aureobasidium* and *Cladosporium* are recognised opportunistic pathogens in immunocompromised individuals (Hawkes et al., 2005; Mise et al., 2008; Ibrahim et al., 2022), whereas *Wickerhamomyces* is an emerging pathogen associated with infections in vulnerable hosts (Ioannou et al., 2024).

Conversely, paediatric patients showed reduced levels of *Clavispora* and *Vishniacozyma*, fungi that our previous work found decreased in adults with UC compared with healthy controls (Scanu et al., 2024b), suggesting a potential protective role in gut homeostasis. *Betamyces*, belonging to the Chytridiomycota phylum, was also reduced in paediatric patients and has been reported as increased in individuals with neurodevelopmental disorders (Laswi et al., 2021). *Fusicolla* and *Aphelidium*, known from environmental samples and pig intestines respectively (Luo et al., 2021; Liu et al., 2022), are described here for the first time in the human gut. Correlation analyses further confirmed age-dependent trends: *Vishniacozyma, Cutaneotrichosporon*, and *Clavispora* positively correlated with age, whereas *Cladosporium, Aureobasidium*, and Saccharomycetes showed negative correlations.

Several IBD-risk genes are directly involved in antifungal immune responses, supporting a plausible role for fungi in disease development (Richard et al., 2015). Experimental evidence strengthens this hypothesis: in mice, fluconazole-induced fungal dysbiosis exacerbates colitis (Wheeler et al., 2016), and fungi are known to modulate pathobiont expansion, immune pathways, and host metabolism in UC (Ni et al., 2017).

In contrast to the clear age-related differences observed in the mycobiome, bacterial diversity remained largely stable across age groups, with minimal biologically meaningful differences observed. No bacterial taxa differed between groups, no age-related correlations emerged, and machine-learning models failed to classify patients by age based on bacteria alone. This pattern highlights a kingdom-specific response in the gut ecosystem, where age-related shifts predominantly involve fungi while the bacterial microbiota remains comparatively conserved. Network analyses further underscored the divergent ecosystem structures. The paediatric cohort exhibited fewer fungus–bacterium interactions, predominantly negative, suggesting a more disrupted microbial environment. Notably, *Candida* showed a negative correlation with Clostridium, consistent with an antagonistic relationship in which fungal overgrowth coincides with the loss of beneficial SCFA-producing bacteria, impairing epithelial barrier integrity and amplifying inflammation.

In adults, networks were more numerous and largely positive. Notably, the observed negative correlation between *Moraxella* and *Malassezia* may suggest a potential interaction, although its functional significance remains unclear and requires further investigation.

Analysis of the predicted functional role of the gut bacterial microbiota suggested inflammatory-associated metabolic potentials in both paediatric and adult UC cohorts. In children, predicted enrichment of pathways related to lipopolysaccharides and activation of pathways such as nitrogen metabolism, ubiquinone and terpenoid-quinone biosynthesis, ascorbate and aldarate metabolism, and peroxisome function may indicate microbial functions potentially linked to oxidative stress and mucosal inflammation. In adults, predicted enrichment of pathways associated with bacterial pathogenicity, cell wall synthesis, host–microbe interactions, and upregulation of transcriptional and DNA repair mechanisms, reflecting microbial adaptation to chronic inflammatory and oxidative stress. These findings should be interpreted cautiously, as pathway inference was based on phylogenetic prediction rather than direct metagenomic measurement.

To further explore how clinical and microbial features vary across the disease spectrum, patients were stratified according to life stage: childhood, adolescence, young adulthood, adulthood, and late adulthood. Age-stratified analyses showed that young adults displayed higher disease severity, both clinically and endoscopically, together with increased Basidiomycota/Ascomycota and Firmicutes/Bacteroidota ratios, suggesting a pattern of more pronounced bacterial and fungal dysbiosis within this subgroup. Across life stages, the transition from childhood to adulthood was accompanied by reduced *F. prausnitzii* abundance and higher lysozyme levels, indicating concurrent changes in microbial composition and mucosal immune responses. Previous studies have reported increased Basidiomycota/Ascomycota ratios in IBD, particularly during active disease (Catalán-Serra et al., 2024), while elevated Firmicutes/Bacteroidota ratios have been associated with UC occurrence and progression (Yin et al., 2025). In this context, the observed patterns may reflect the combined influence of life stage and disease-related factors rather than age alone. Consistently, *F. prausnitzii*, a major butyrate producer and marker of intestinal homeostasis (Miquel et al., 2013), showed a negative correlation with the Mayo score, supporting its potential protective role within the inflammatory environment. This study represents, to our knowledge, the first simultaneous evaluation faecal secretory IgA (sIgA) and lysozyme in UC patients. sIgA, the predominant immunoglobulin in the intestinal lumen, promotes immune tolerance and limits pathogen adhesion without inducing inflammation (Kaetzel, 2014), while also contributing to the maintenance of fungal commensalism (Sun et al., 2023). Consistent with previous reports, increased faecal sIgA levels in UC likely reflect enhanced mucosal immune activation during inflammatory states (Lin et al., 2018; Masu et al., 2021).

Lysozyme, an antimicrobial enzyme released by activated epithelial and immune cells, is commonly elevated in UC and correlates with disease activity, reflecting ongoing mucosal inflammation and barrier disruption (Veer et al., 1998; Lloyd-Price et al., 2019).

The positive correlation observed between *F. prausnitzii* and lysozyme may appear counterintuitive the widely recognised anti-inflammatory role of *F. prausnitzii*, and the association of lysozyme with inflammatory responses. However, most evidence describing *F. prausnitzii* depletion derives from comparisons between IBD patients and healthy controls, whereas the present cohort includes only UC patients. Within a disease-only context, variability in *F. prausnitzii* abundance may reflect adaptive or compensatory microbial responses rather than simple loss of anti-inflammatory taxa.

Moreover, lysozyme should not be interpreted solely as a marker of inflammation, as it also participates in mucosal defence and microbiota modulation. The observed association may therefore indicate a context-dependent response, potentially reflecting a parallel upregulation of host defence mechanisms and beneficial microbial populations during active disease, or a distinctive feature of UC-associated microbial–immune dynamics.

Overall, these findings emphasise the complexity of host–microbiome interactions in UC and highlight the complementary roles of sIgA and lysozyme in shaping mucosal homeostasis. The concurrent variation of these immune markers across life stages may reflect interactions between host maturation, immune adaptation, and microbial configuration, rather than disease severity alone.

Both sIgA and lysozyme correlated with gut microbial composition, with sIgA positively linked to selected bacteria (*Bacteroides* H, CAG-508, *Romboutsia*) and fungi (*Aureobasidium*), and lysozyme positively associated with SCFA-producing bacteria (e.g., *Alistipes A871400*, *Bacteroides H*, *Collinsella*, *Phocaeicola* A858004, *Agathobacter* 164117, and *Roseburia*) while negatively correlating with opportunistic fungi (*Aureobasidium*), indicating coordinated regulation of bacterial–fungal balance and support of anti-inflammatory taxa.

These results highlight distinct patterns of gut dysbiosis across life stages in UC. Paediatric patients exhibited a more unstable microbial configuration characterized by pro-inflammatory signatures, loss of butyrate-producing bacteria, and expansion of opportunistic fungi. Adults, in contrast, showed a more chronic and resilient dysbiotic state consistent with long-standing inflammation. The transition from childhood to adulthood appeared accompanied by ecological shifts involving both microbial communities and mucosal immune responses, suggesting an interaction between host maturation and microbial adaptation.

This study has several limitations. The cohort size was moderate, and patients were recruited at different stages of disease while receiving ongoing treatments that may influence microbial composition. Detailed dietary and lifestyle data were unavailable, although participants shared broadly similar Mediterranean dietary habits. Functional predictions based on targeted metagenomics require validation through shotgun sequencing.

An additional limitation is the imbalance in disease severity between paediatric and adult cohorts, which represents a potential confounder in age-related comparisons. However, analyses accounting for disease activity suggested that the observed mycobiome variation cannot be attributed solely to inflammatory status, supporting the interpretation of age as an ecological factor acting within the clinical context of UC. Future longitudinal studies in clinically balanced populations will be necessary to confirm these findings.

## 5 Conclusions

Both paediatric and adult UC patients exhibit dysbiosis, but with distinct ecological characteristics. Fungal communities appear more sensitive to life stage than bacterial communities, suggesting a stronger interaction between host development, immunity, and mycobiome structure. Considering life stage alongside clinical context may improve interpretation of microbiome alterations and support the development of age-informed microbiome-based strategies in UC.

## Data Availability

The original contributions presented in the study are included in the article/supplementary material. Further inquiries can be directed to the corresponding author.
