# Supplementary material for: Ecological patterns of the gut mycobiome and microbiome in ulcerative colitis across life stages
Source: Front Cell Infect Microbiol. 2026 Apr 28;16:1769892. doi: 10.3389/fcimb.2026.1769892 (PMC13161133; doi:10.3389/fcimb.2026.1769892)
Supplement: Supplementary file 1 [file DataSheet1.pdf]

Supplementary Material

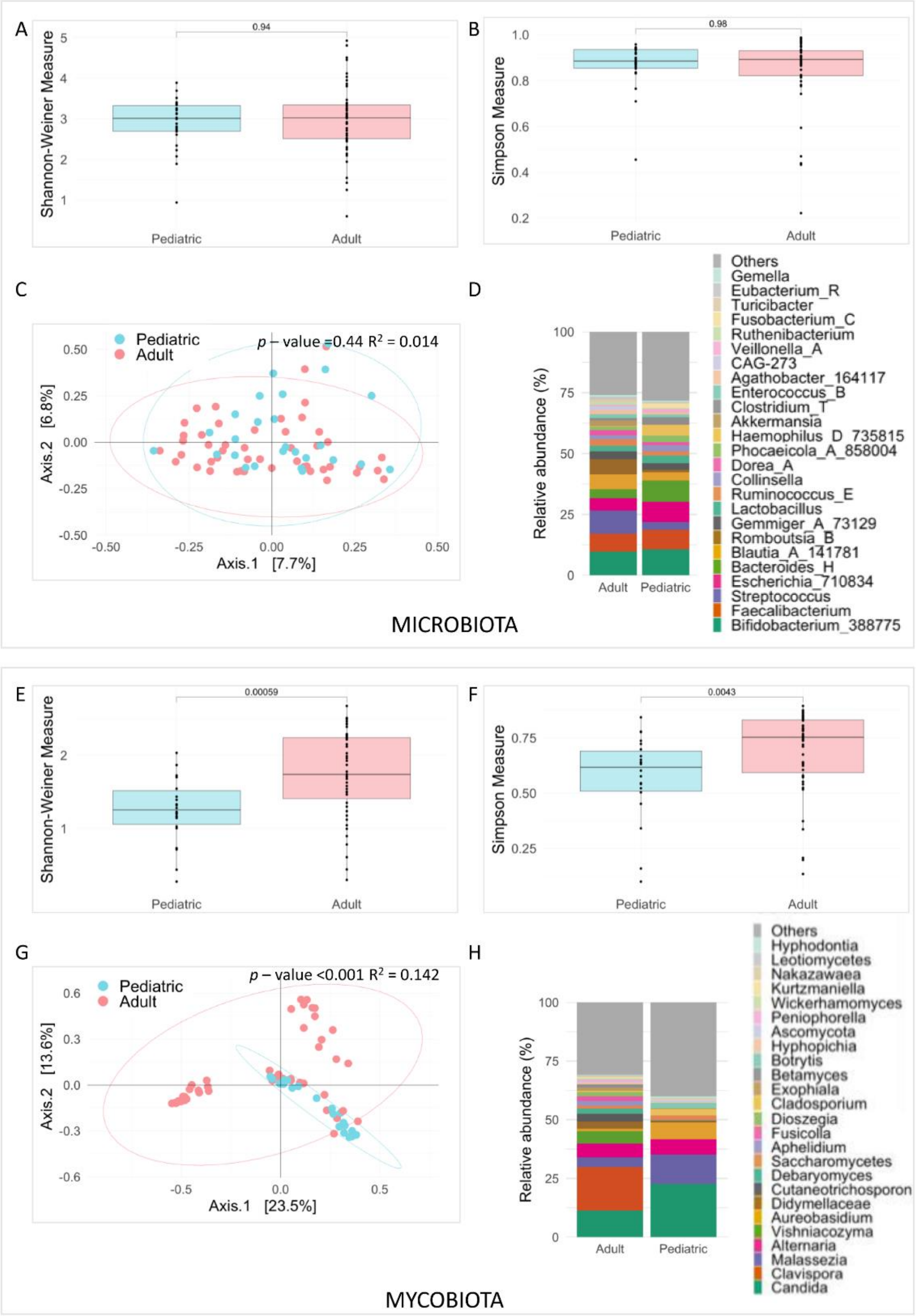

Figure S1. Diversity and taxonomic comparison of bacterial and fungal gut microbiota in paediatric and adult ulcerative colitis (UC) patients. (A–B) Alpha-diversity indices showed no significant differences in bacterial community diversity between paediatric and adult patients. (C)

Beta-diversity analysis based on Bray–Curtis dissimilarity revealed no significant separation between groups (PERMANOVA $p = 0.44$; $R^2 = 0.014$). (D) Genus-level bacterial composition displayed only minor variations between cohorts. (E–F) In contrast, fungal alpha-diversity was higher in paediatric patients compared with adults. (G) Bray–Curtis beta-diversity analysis demonstrated significant differences in fungal community structure between groups (PERMANOVA $p = 0.001$; $R^2 = 0.142$), with paediatric samples forming a distinct cluster and adult samples distributed across multiple subclusters. (H) Genus-level fungal composition differed between paediatric and adult UC patients.

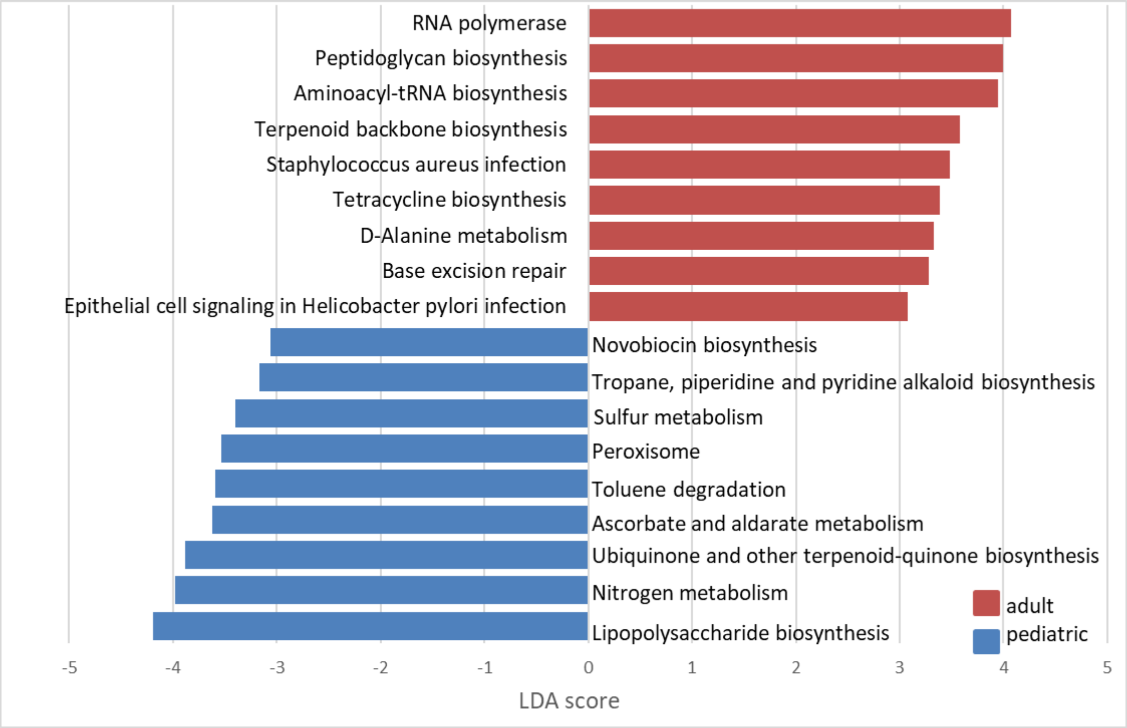

Figure S2. Predicted functional profiles of the gut bacterial microbiota inferred by PICRUSt analysis. Functional metagenomic prediction was performed using PICRUSt (Phylogenetic Investigation of Communities by Reconstruction of Unobserved States) based on 16S rRNA gene sequencing data. Relative abundances of major KEGG metabolic pathways are shown for paediatric and adult UC patients. Differentially enriched functional categories were identified using LEfSe analysis (LDA score > 3.0; $p < 0.05$).

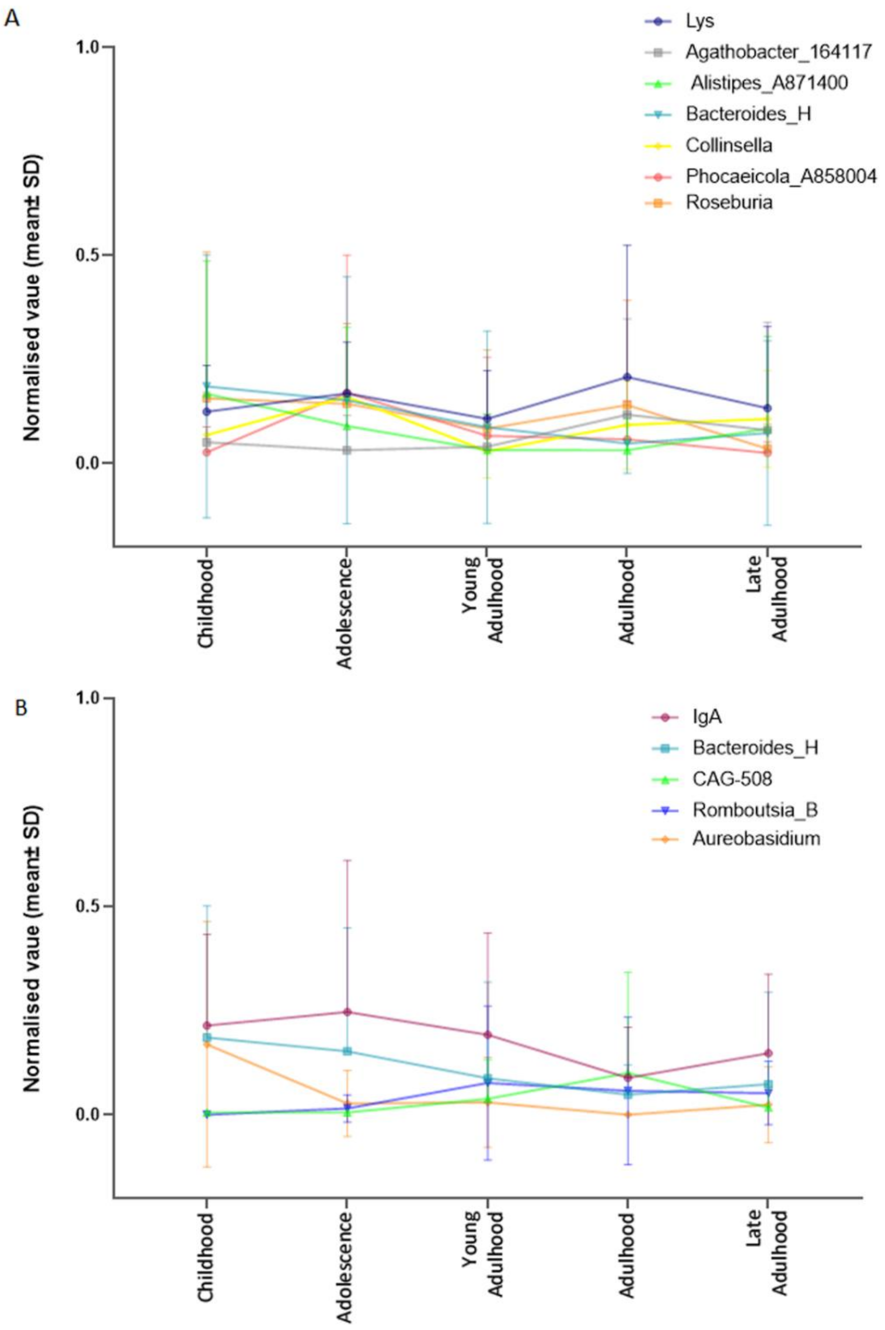

Figure S3. Age-associated trends of microbial ASVs correlated with fecal immune markers in ulcerative colitis (UC) patients. (A) Mean (±SD) abundance of selected microorganisms significantly associated with faecal lysozyme levels across five age-defined stages (childhood, adolescence, young adulthood, adulthood, and late adulthood). (B) Mean abundance of microorganisms correlated with secretory IgA (sIgA) levels across the same age-defined stages. Each line represents an individual microorganism, illustrating temporal patterns consistent with potential microbiota–immune interactions. Values were min–max normalized to allow comparison across taxa with different abundance scales.
