# Supplementary material for: Ecological patterns of the gut mycobiome and microbiome in ulcerative colitis across life stages
Source: Front Cell Infect Microbiol. 2026 Apr 28;16:1769892. doi: 10.3389/fcimb.2026.1769892 (PMC13161133; doi:10.3389/fcimb.2026.1769892)
Supplement: Supplementary file 2 [file DataSheet2.pdf]

NestedCV_PerFold										
Outer Resample	Outer Repeat	Outer Fold	Method	Train N	Test N	Accuracy	Recall	Precision	F1 Score	AUC
Fold1.Rep01	Rep01	Fold1	RM	55	14	1.00	1.00	1.00	1.00	1.00
Fold1.Rep01	Rep01	Fold1	GBM	55	14	1.00	1.00	1.00	1.00	1.00
Fold1.Rep01	Rep01	Fold1	SVM	55	14	0.93	1.00	0.80	0.89	0.90
Fold2.Rep01	Rep01	Fold2	RM	56	13	1.00	1.00	1.00	1.00	1.00
Fold2.Rep01	Rep01	Fold2	GBM	56	13	1.00	1.00	1.00	1.00	1.00
Fold2.Rep01	Rep01	Fold2	SVM	56	13	0.85	0.75	0.75	0.75	0.83
Fold3.Rep01	Rep01	Fold3	RM	55	14	1.00	1.00	1.00	1.00	1.00
Fold3.Rep01	Rep01	Fold3	GBM	55	14	1.00	1.00	1.00	1.00	1.00
Fold3.Rep01	Rep01	Fold3	SVM	55	14	0.93	0.80	1.00	0.89	1.00
Fold4.Rep01	Rep01	Fold4	RM	55	14	1.00	1.00	1.00	1.00	1.00
Fold4.Rep01	Rep01	Fold4	GBM	55	14	1.00	1.00	1.00	1.00	1.00
Fold4.Rep01	Rep01	Fold4	SVM	55	14	0.86	0.60	1.00	0.75	0.96
Fold5.Rep01	Rep01	Fold5	RM	55	14	1.00	1.00	1.00	1.00	1.00
Fold5.Rep01	Rep01	Fold5	GBM	55	14	1.00	1.00	1.00	1.00	1.00
Fold5.Rep01	Rep01	Fold5	SVM	55	14	0.79	0.50	0.67	0.57	0.73
Fold1.Rep02	Rep02	Fold1	RM	55	14	1.00	1.00	1.00	1.00	1.00
Fold1.Rep02	Rep02	Fold1	GBM	55	14	1.00	1.00	1.00	1.00	1.00
Fold1.Rep02	Rep02	Fold1	SVM	55	14	0.79	0.60	0.75	0.67	0.64
Fold2.Rep02	Rep02	Fold2	RM	55	14	1.00	1.00	1.00	1.00	1.00
Fold2.Rep02	Rep02	Fold2	GBM	55	14	1.00	1.00	1.00	1.00	1.00
Fold2.Rep02	Rep02	Fold2	SVM	55	14	0.79	0.60	0.75	0.67	0.89
Fold3.Rep02	Rep02	Fold3	RM	56	13	1.00	1.00	1.00	1.00	1.00
Fold3.Rep02	Rep02	Fold3	GBM	56	13	0.92	1.00	0.80	0.89	1.00
Fold3.Rep02	Rep02	Fold3	SVM	56	13	0.92	1.00	0.80	0.89	0.94
Fold4.Rep02	Rep02	Fold4	RM	55	14	1.00	1.00	1.00	1.00	1.00
Fold4.Rep02	Rep02	Fold4	GBM	55	14	1.00	1.00	1.00	1.00	1.00
Fold4.Rep02	Rep02	Fold4	SVM	55	14	0.93	0.75	1.00	0.86	0.95
Fold5.Rep02	Rep02	Fold5	RM	55	14	1.00	1.00	1.00	1.00	1.00
Fold5.Rep02	Rep02	Fold5	GBM	55	14	1.00	1.00	1.00	1.00	1.00
Fold5.Rep02	Rep02	Fold5	SVM	55	14	0.71	0.50	0.50	0.50	0.80
Fold1.Rep03	Rep03	Fold1	RM	56	13	1.00	1.00	1.00	1.00	1.00
Fold1.Rep03	Rep03	Fold1	GBM	56	13	1.00	1.00	1.00	1.00	1.00
Fold1.Rep03	Rep03	Fold1	SVM	56	13	0.77	0.50	0.67	0.57	0.83
Fold2.Rep03	Rep03	Fold2	RM	55	14	1.00	1.00	1.00	1.00	1.00
Fold2.Rep03	Rep03	Fold2	GBM	55	14	1.00	1.00	1.00	1.00	1.00
Fold2.Rep03	Rep03	Fold2	SVM	55	14	0.64	0.25	0.33	0.29	0.80
Fold3.Rep03	Rep03	Fold3	RM	55	14	1.00	1.00	1.00	1.00	1.00
Fold3.Rep03	Rep03	Fold3	GBM	55	14	1.00	1.00	1.00	1.00	1.00
Fold3.Rep03	Rep03	Fold3	SVM	55	14	1.00	1.00	1.00	1.00	1.00
Fold4.Rep03	Rep03	Fold4	RM	54	15	1.00	1.00	1.00	1.00	1.00
Fold4.Rep03	Rep03	Fold4	GBM	54	15	1.00	1.00	1.00	1.00	1.00
Fold4.Rep03	Rep03	Fold4	SVM	54	15	0.80	0.80	0.67	0.73	0.90
Fold5.Rep03	Rep03	Fold5	RM	56	13	1.00	1.00	1.00	1.00	1.00
Fold5.Rep03	Rep03	Fold5	GBM	56	13	1.00	1.00	1.00	1.00	1.00
Fold5.Rep03	Rep03	Fold5	SVM	56	13	0.62	0.25	0.33	0.29	0.75
Fold1.Rep04	Rep04	Fold1	RM	56	13	1.00	1.00	1.00	1.00	1.00
Fold1.Rep04	Rep04	Fold1	GBM	56	13	1.00	1.00	1.00	1.00	1.00
Fold1.Rep04	Rep04	Fold1	SVM	56	13	0.85	0.75	0.75	0.75	0.89
Fold2.Rep04	Rep04	Fold2	RM	56	13	1.00	1.00	1.00	1.00	1.00
Fold2.Rep04	Rep04	Fold2	GBM	56	13	1.00	1.00	1.00	1.00	1.00
Fold2.Rep04	Rep04	Fold2	SVM	56	13	0.62	0.50	0.40	0.44	0.75
Fold3.Rep04	Rep04	Fold3	RM	55	14	1.00	1.00	1.00	1.00	1.00
Fold3.Rep04	Rep04	Fold3	GBM	55	14	1.00	1.00	1.00	1.00	1.00
Fold3.Rep04	Rep04	Fold3	SVM	55	14	0.86	0.50	1.00	0.67	0.90
Fold4.Rep04	Rep04	Fold4	RM	55	14	1.00	1.00	1.00	1.00	1.00
Fold4.Rep04	Rep04	Fold4	GBM	55	14	1.00	1.00	1.00	1.00	1.00

Fold4.Rep04	Rep04	Fold4	SVM	55	14	0.93	0.80	1.00	0.89	1.00
Fold5.Rep04	Rep04	Fold5	RM	54	15	1.00	1.00	1.00	1.00	1.00
Fold5.Rep04	Rep04	Fold5	GBM	54	15	1.00	1.00	1.00	1.00	1.00
Fold5.Rep04	Rep04	Fold5	SVM	54	15	0.93	1.00	0.83	0.91	0.91
Fold1.Rep05	Rep05	Fold1	RM	55	14	1.00	1.00	1.00	1.00	1.00
Fold1.Rep05	Rep05	Fold1	GBM	55	14	1.00	1.00	1.00	1.00	1.00
Fold1.Rep05	Rep05	Fold1	SVM	55	14	0.79	0.80	0.67	0.73	0.82
Fold2.Rep05	Rep05	Fold2	RM	55	14	1.00	1.00	1.00	1.00	1.00
Fold2.Rep05	Rep05	Fold2	GBM	55	14	1.00	1.00	1.00	1.00	1.00
Fold2.Rep05	Rep05	Fold2	SVM	55	14	0.79	0.80	0.67	0.73	0.89
Fold3.Rep05	Rep05	Fold3	RM	55	14	1.00	1.00	1.00	1.00	1.00
Fold3.Rep05	Rep05	Fold3	GBM	55	14	1.00	1.00	1.00	1.00	1.00
Fold3.Rep05	Rep05	Fold3	SVM	55	14	0.79	0.50	0.67	0.57	0.80
Fold4.Rep05	Rep05	Fold4	RM	55	14	1.00	1.00	1.00	1.00	1.00
Fold4.Rep05	Rep05	Fold4	GBM	55	14	1.00	1.00	1.00	1.00	1.00
Fold4.Rep05	Rep05	Fold4	SVM	55	14	0.86	0.75	0.75	0.75	0.88
Fold5.Rep05	Rep05	Fold5	RM	56	13	1.00	1.00	1.00	1.00	1.00
Fold5.Rep05	Rep05	Fold5	GBM	56	13	1.00	1.00	1.00	1.00	1.00
Fold5.Rep05	Rep05	Fold5	SVM	56	13	0.85	0.50	1.00	0.67	1.00
Fold1.Rep06	Rep06	Fold1	RM	55	14	1.00	1.00	1.00	1.00	1.00
Fold1.Rep06	Rep06	Fold1	GBM	55	14	1.00	1.00	1.00	1.00	1.00
Fold1.Rep06	Rep06	Fold1	SVM	55	14	0.71	0.50	0.50	0.50	0.83
Fold2.Rep06	Rep06	Fold2	RM	55	14	1.00	1.00	1.00	1.00	1.00
Fold2.Rep06	Rep06	Fold2	GBM	55	14	1.00	1.00	1.00	1.00	1.00
Fold2.Rep06	Rep06	Fold2	SVM	55	14	0.86	0.80	0.80	0.80	0.93
Fold3.Rep06	Rep06	Fold3	RM	56	13	1.00	1.00	1.00	1.00	1.00
Fold3.Rep06	Rep06	Fold3	GBM	56	13	1.00	1.00	1.00	1.00	1.00
Fold3.Rep06	Rep06	Fold3	SVM	56	13	0.77	0.75	0.60	0.67	0.72
Fold4.Rep06	Rep06	Fold4	RM	54	15	1.00	1.00	1.00	1.00	1.00
Fold4.Rep06	Rep06	Fold4	GBM	54	15	1.00	1.00	1.00	1.00	1.00
Fold4.Rep06	Rep06	Fold4	SVM	54	15	0.87	0.60	1.00	0.75	0.96
Fold5.Rep06	Rep06	Fold5	RM	56	13	1.00	1.00	1.00	1.00	1.00
Fold5.Rep06	Rep06	Fold5	GBM	56	13	1.00	1.00	1.00	1.00	1.00
Fold5.Rep06	Rep06	Fold5	SVM	56	13	0.77	0.75	0.60	0.67	0.75
Fold1.Rep07	Rep07	Fold1	RM	55	14	1.00	1.00	1.00	1.00	1.00
Fold1.Rep07	Rep07	Fold1	GBM	55	14	1.00	1.00	1.00	1.00	1.00
Fold1.Rep07	Rep07	Fold1	SVM	55	14	0.93	1.00	0.80	0.89	0.93
Fold2.Rep07	Rep07	Fold2	RM	56	13	1.00	1.00	1.00	1.00	1.00
Fold2.Rep07	Rep07	Fold2	GBM	56	13	1.00	1.00	1.00	1.00	1.00
Fold2.Rep07	Rep07	Fold2	SVM	56	13	0.92	0.75	1.00	0.86	1.00
Fold3.Rep07	Rep07	Fold3	RM	55	14	1.00	1.00	1.00	1.00	1.00
Fold3.Rep07	Rep07	Fold3	GBM	55	14	1.00	1.00	1.00	1.00	1.00
Fold3.Rep07	Rep07	Fold3	SVM	55	14	0.71	0.25	0.50	0.33	0.93
Fold4.Rep07	Rep07	Fold4	RM	55	14	1.00	1.00	1.00	1.00	1.00
Fold4.Rep07	Rep07	Fold4	GBM	55	14	1.00	1.00	1.00	1.00	1.00
Fold4.Rep07	Rep07	Fold4	SVM	55	14	0.64	0.60	0.50	0.55	0.69
Fold5.Rep07	Rep07	Fold5	RM	55	14	1.00	1.00	1.00	1.00	1.00
Fold5.Rep07	Rep07	Fold5	GBM	55	14	1.00	1.00	1.00	1.00	1.00
Fold5.Rep07	Rep07	Fold5	SVM	55	14	0.79	0.80	0.67	0.73	0.87
Fold1.Rep08	Rep08	Fold1	RM	55	14	1.00	1.00	1.00	1.00	1.00
Fold1.Rep08	Rep08	Fold1	GBM	55	14	1.00	1.00	1.00	1.00	1.00
Fold1.Rep08	Rep08	Fold1	SVM	55	14	0.93	0.75	1.00	0.86	0.90
Fold2.Rep08	Rep08	Fold2	RM	54	15	1.00	1.00	1.00	1.00	1.00
Fold2.Rep08	Rep08	Fold2	GBM	54	15	1.00	1.00	1.00	1.00	1.00
Fold2.Rep08	Rep08	Fold2	SVM	54	15	0.93	0.80	1.00	0.89	1.00
Fold3.Rep08	Rep08	Fold3	RM	56	13	1.00	1.00	1.00	1.00	1.00
Fold3.Rep08	Rep08	Fold3	GBM	56	13	1.00	1.00	1.00	1.00	1.00
Fold3.Rep08	Rep08	Fold3	SVM	56	13	0.69	0.75	0.50	0.60	0.69
Fold4.Rep08	Rep08	Fold4	RM	56	13	1.00	1.00	1.00	1.00	1.00

Fold4.Rep08	Rep08	Fold4	GBM	56	13	1.00	1.00	1.00	1.00	1.00
Fold4.Rep08	Rep08	Fold4	SVM	56	13	0.92	1.00	0.80	0.89	0.89
Fold5.Rep08	Rep08	Fold5	RM	55	14	1.00	1.00	1.00	1.00	1.00
Fold5.Rep08	Rep08	Fold5	GBM	55	14	1.00	1.00	1.00	1.00	1.00
Fold5.Rep08	Rep08	Fold5	SVM	55	14	0.79	0.60	0.75	0.67	0.89
Fold1.Rep09	Rep09	Fold1	RM	56	13	1.00	1.00	1.00	1.00	1.00
Fold1.Rep09	Rep09	Fold1	GBM	56	13	1.00	1.00	1.00	1.00	1.00
Fold1.Rep09	Rep09	Fold1	SVM	56	13	0.77	0.50	0.67	0.57	0.89
Fold2.Rep09	Rep09	Fold2	RM	54	15	1.00	1.00	1.00	1.00	1.00
Fold2.Rep09	Rep09	Fold2	GBM	54	15	1.00	1.00	1.00	1.00	1.00
Fold2.Rep09	Rep09	Fold2	SVM	54	15	0.73	0.40	0.67	0.50	0.63
Fold3.Rep09	Rep09	Fold3	RM	56	13	1.00	1.00	1.00	1.00	1.00
Fold3.Rep09	Rep09	Fold3	GBM	56	13	1.00	1.00	1.00	1.00	1.00
Fold3.Rep09	Rep09	Fold3	SVM	56	13	0.85	0.75	0.75	0.75	0.94
Fold4.Rep09	Rep09	Fold4	RM	55	14	1.00	1.00	1.00	1.00	1.00
Fold4.Rep09	Rep09	Fold4	GBM	55	14	0.93	1.00	0.80	0.89	1.00
Fold4.Rep09	Rep09	Fold4	SVM	55	14	0.79	1.00	0.57	0.73	0.88
Fold5.Rep09	Rep09	Fold5	RM	55	14	1.00	1.00	1.00	1.00	1.00
Fold5.Rep09	Rep09	Fold5	GBM	55	14	1.00	1.00	1.00	1.00	1.00
Fold5.Rep09	Rep09	Fold5	SVM	55	14	0.79	0.40	1.00	0.57	0.96
Fold1.Rep10	Rep10	Fold1	RM	55	14	1.00	1.00	1.00	1.00	1.00
Fold1.Rep10	Rep10	Fold1	GBM	55	14	1.00	1.00	1.00	1.00	1.00
Fold1.Rep10	Rep10	Fold1	SVM	55	14	0.86	0.75	0.75	0.75	0.98
Fold2.Rep10	Rep10	Fold2	RM	55	14	1.00	1.00	1.00	1.00	1.00
Fold2.Rep10	Rep10	Fold2	GBM	55	14	1.00	1.00	1.00	1.00	1.00
Fold2.Rep10	Rep10	Fold2	SVM	55	14	0.86	0.60	1.00	0.75	0.93
Fold3.Rep10	Rep10	Fold3	RM	56	13	1.00	1.00	1.00	1.00	1.00
Fold3.Rep10	Rep10	Fold3	GBM	56	13	1.00	1.00	1.00	1.00	1.00
Fold3.Rep10	Rep10	Fold3	SVM	56	13	0.69	0.25	0.50	0.33	0.81
Fold4.Rep10	Rep10	Fold4	RM	56	13	1.00	1.00	1.00	1.00	1.00
Fold4.Rep10	Rep10	Fold4	GBM	56	13	1.00	1.00	1.00	1.00	1.00
Fold4.Rep10	Rep10	Fold4	SVM	56	13	0.85	0.75	0.75	0.75	0.89
Fold5.Rep10	Rep10	Fold5	RM	54	15	1.00	1.00	1.00	1.00	1.00
Fold5.Rep10	Rep10	Fold5	GBM	54	15	1.00	1.00	1.00	1.00	1.00
Fold5.Rep10	Rep10	Fold5	SVM	54	15	0.80	0.80	0.67	0.73	0.90

NestedCV_Summary			
**Method**	**GBM**	**Random Forest**	**SVM**
_Outer_Evaluatioı	50.00	50.00	50.00
Accuracy_Mean	1.00	1.00	0.82
Accuracy_SD	0.01	0.00	0.09
Recall_Mean	1.00	1.00	0.67
Recall_SD	0.00	0.00	0.21
Precision_Mean	0.99	1.00	0.74
Precision_SD	0.04	0.00	0.19
F1_Mean	1.00	1.00	0.69
F1_SD	0.02	0.00	0.17
AUC_Mean	1.00	1.00	0.87
AUC_SD	0.00	0.00	0.10
AUC_Median	1.00	1.00	0.89
AUC_IQR	0.00	0.00	0.13
AUC_Min	1.00	1.00	0.63
AUC_Max	1.00	1.00	1.00
Prop_AUC_1	1.00	1.00	0.12

NestedCV_Summary						
Outer Resample	Outer Repeat	Outer Fold	Method	Truth	Predicted Class	Prob Pediatric
Fold1.Rep01	Rep01	Fold1	RM	Adult	Adult	0.028
Fold1.Rep01	Rep01	Fold1	RM	Adult	Adult	0.134
Fold1.Rep01	Rep01	Fold1	RM	Adult	Adult	0.070
Fold1.Rep01	Rep01	Fold1	RM	Adult	Adult	0.092
Fold1.Rep01	Rep01	Fold1	RM	Adult	Adult	0.092
Fold1.Rep01	Rep01	Fold1	RM	Adult	Adult	0.234
Fold1.Rep01	Rep01	Fold1	RM	Pediatric	Pediatric	0.812
Fold1.Rep01	Rep01	Fold1	RM	Pediatric	Pediatric	0.908
Fold1.Rep01	Rep01	Fold1	RM	Pediatric	Pediatric	0.862
Fold1.Rep01	Rep01	Fold1	RM	Pediatric	Pediatric	0.580
Fold1.Rep01	Rep01	Fold1	RM	Adult	Adult	0.042
Fold1.Rep01	Rep01	Fold1	RM	Adult	Adult	0.102
Fold1.Rep01	Rep01	Fold1	RM	Adult	Adult	0.038
Fold1.Rep01	Rep01	Fold1	RM	Adult	Adult	0.080
Fold1.Rep01	Rep01	Fold1	GBM	Adult	Adult	0.006
Fold1.Rep01	Rep01	Fold1	GBM	Adult	Adult	0.006
Fold1.Rep01	Rep01	Fold1	GBM	Adult	Adult	0.006
Fold1.Rep01	Rep01	Fold1	GBM	Adult	Adult	0.004
Fold1.Rep01	Rep01	Fold1	GBM	Adult	Adult	0.003
Fold1.Rep01	Rep01	Fold1	GBM	Adult	Adult	0.010
Fold1.Rep01	Rep01	Fold1	GBM	Pediatric	Pediatric	0.972
Fold1.Rep01	Rep01	Fold1	GBM	Pediatric	Pediatric	0.987
Fold1.Rep01	Rep01	Fold1	GBM	Pediatric	Pediatric	0.987
Fold1.Rep01	Rep01	Fold1	GBM	Pediatric	Pediatric	0.942
Fold1.Rep01	Rep01	Fold1	GBM	Adult	Adult	0.002
Fold1.Rep01	Rep01	Fold1	GBM	Adult	Adult	0.006
Fold1.Rep01	Rep01	Fold1	GBM	Adult	Adult	0.002
Fold1.Rep01	Rep01	Fold1	GBM	Adult	Adult	0.005
Fold1.Rep01	Rep01	Fold1	SVM	Adult	Adult	0.007
Fold1.Rep01	Rep01	Fold1	SVM	Adult	Pediatric	1.000
Fold1.Rep01	Rep01	Fold1	SVM	Adult	Adult	0.118
Fold1.Rep01	Rep01	Fold1	SVM	Adult	Adult	0.048
Fold1.Rep01	Rep01	Fold1	SVM	Adult	Adult	0.000
Fold1.Rep01	Rep01	Fold1	SVM	Adult	Adult	0.000
Fold1.Rep01	Rep01	Fold1	SVM	Pediatric	Pediatric	0.719
Fold1.Rep01	Rep01	Fold1	SVM	Pediatric	Pediatric	0.675
Fold1.Rep01	Rep01	Fold1	SVM	Pediatric	Pediatric	0.726
Fold1.Rep01	Rep01	Fold1	SVM	Pediatric	Pediatric	0.738
Fold1.Rep01	Rep01	Fold1	SVM	Adult	Adult	0.006
Fold1.Rep01	Rep01	Fold1	SVM	Adult	Adult	0.073
Fold1.Rep01	Rep01	Fold1	SVM	Adult	Adult	0.022
Fold1.Rep01	Rep01	Fold1	SVM	Adult	Adult	0.045
Fold2.Rep01	Rep01	Fold2	RM	Adult	Adult	0.022
Fold2.Rep01	Rep01	Fold2	RM	Adult	Adult	0.032
Fold2.Rep01	Rep01	Fold2	RM	Adult	Adult	0.012
Fold2.Rep01	Rep01	Fold2	RM	Adult	Adult	0.156
Fold2.Rep01	Rep01	Fold2	RM	Adult	Adult	0.222
Fold2.Rep01	Rep01	Fold2	RM	Pediatric	Pediatric	0.856
Fold2.Rep01	Rep01	Fold2	RM	Pediatric	Pediatric	0.764
Fold2.Rep01	Rep01	Fold2	RM	Pediatric	Pediatric	0.852
Fold2.Rep01	Rep01	Fold2	RM	Pediatric	Pediatric	0.860
Fold2.Rep01	Rep01	Fold2	RM	Adult	Adult	0.112
Fold2.Rep01	Rep01	Fold2	RM	Adult	Adult	0.072
Fold2.Rep01	Rep01	Fold2	RM	Adult	Adult	0.218
Fold2.Rep01	Rep01	Fold2	RM	Adult	Adult	0.154

Fold2.Rep01	Rep01	Fold2	GBM	Adult	Adult	0.011
Fold2.Rep01	Rep01	Fold2	GBM	Adult	Adult	0.002
Fold2.Rep01	Rep01	Fold2	GBM	Adult	Adult	0.002
Fold2.Rep01	Rep01	Fold2	GBM	Adult	Adult	0.006
Fold2.Rep01	Rep01	Fold2	GBM	Adult	Adult	0.011
Fold2.Rep01	Rep01	Fold2	GBM	Pediatric	Pediatric	0.986
Fold2.Rep01	Rep01	Fold2	GBM	Pediatric	Pediatric	0.986
Fold2.Rep01	Rep01	Fold2	GBM	Pediatric	Pediatric	0.986
Fold2.Rep01	Rep01	Fold2	GBM	Pediatric	Pediatric	0.960
Fold2.Rep01	Rep01	Fold2	GBM	Adult	Adult	0.011
Fold2.Rep01	Rep01	Fold2	GBM	Adult	Adult	0.009
Fold2.Rep01	Rep01	Fold2	GBM	Adult	Adult	0.027
Fold2.Rep01	Rep01	Fold2	GBM	Adult	Adult	0.005
Fold2.Rep01	Rep01	Fold2	SVM	Adult	Adult	0.186
Fold2.Rep01	Rep01	Fold2	SVM	Adult	Adult	0.001
Fold2.Rep01	Rep01	Fold2	SVM	Adult	Adult	0.148
Fold2.Rep01	Rep01	Fold2	SVM	Adult	Adult	0.400
Fold2.Rep01	Rep01	Fold2	SVM	Adult	Pediatric	1.000
Fold2.Rep01	Rep01	Fold2	SVM	Pediatric	Pediatric	0.653
Fold2.Rep01	Rep01	Fold2	SVM	Pediatric	Adult	0.299
Fold2.Rep01	Rep01	Fold2	SVM	Pediatric	Pediatric	0.664
Fold2.Rep01	Rep01	Fold2	SVM	Pediatric	Pediatric	0.800
Fold2.Rep01	Rep01	Fold2	SVM	Adult	Adult	0.370
Fold2.Rep01	Rep01	Fold2	SVM	Adult	Adult	0.282
Fold2.Rep01	Rep01	Fold2	SVM	Adult	Adult	0.244
Fold2.Rep01	Rep01	Fold2	SVM	Adult	Adult	0.015
Fold3.Rep01	Rep01	Fold3	RM	Adult	Adult	0.030
Fold3.Rep01	Rep01	Fold3	RM	Adult	Adult	0.016
Fold3.Rep01	Rep01	Fold3	RM	Adult	Adult	0.028
Fold3.Rep01	Rep01	Fold3	RM	Adult	Adult	0.324
Fold3.Rep01	Rep01	Fold3	RM	Adult	Adult	0.124
Fold3.Rep01	Rep01	Fold3	RM	Pediatric	Pediatric	0.768
Fold3.Rep01	Rep01	Fold3	RM	Pediatric	Pediatric	0.860
Fold3.Rep01	Rep01	Fold3	RM	Pediatric	Pediatric	0.660
Fold3.Rep01	Rep01	Fold3	RM	Pediatric	Pediatric	0.646
Fold3.Rep01	Rep01	Fold3	RM	Pediatric	Pediatric	0.846
Fold3.Rep01	Rep01	Fold3	RM	Adult	Adult	0.174
Fold3.Rep01	Rep01	Fold3	RM	Adult	Adult	0.038
Fold3.Rep01	Rep01	Fold3	RM	Adult	Adult	0.064
Fold3.Rep01	Rep01	Fold3	RM	Adult	Adult	0.092
Fold3.Rep01	Rep01	Fold3	GBM	Adult	Adult	0.011
Fold3.Rep01	Rep01	Fold3	GBM	Adult	Adult	0.002
Fold3.Rep01	Rep01	Fold3	GBM	Adult	Adult	0.002
Fold3.Rep01	Rep01	Fold3	GBM	Adult	Adult	0.028
Fold3.Rep01	Rep01	Fold3	GBM	Adult	Adult	0.002
Fold3.Rep01	Rep01	Fold3	GBM	Pediatric	Pediatric	0.979
Fold3.Rep01	Rep01	Fold3	GBM	Pediatric	Pediatric	0.975
Fold3.Rep01	Rep01	Fold3	GBM	Pediatric	Pediatric	0.926
Fold3.Rep01	Rep01	Fold3	GBM	Pediatric	Pediatric	0.790
Fold3.Rep01	Rep01	Fold3	GBM	Pediatric	Pediatric	0.978
Fold3.Rep01	Rep01	Fold3	GBM	Adult	Adult	0.004
Fold3.Rep01	Rep01	Fold3	GBM	Adult	Adult	0.002
Fold3.Rep01	Rep01	Fold3	GBM	Adult	Adult	0.005
Fold3.Rep01	Rep01	Fold3	GBM	Adult	Adult	0.025
Fold3.Rep01	Rep01	Fold3	SVM	Adult	Adult	0.258
Fold3.Rep01	Rep01	Fold3	SVM	Adult	Adult	0.215
Fold3.Rep01	Rep01	Fold3	SVM	Adult	Adult	0.070
Fold3.Rep01	Rep01	Fold3	SVM	Adult	Adult	0.004
Fold3.Rep01	Rep01	Fold3	SVM	Adult	Adult	0.212

Fold3.Rep01	Rep01	Fold3	SVM	Pediatric	Pediatric	0.541
Fold3.Rep01	Rep01	Fold3	SVM	Pediatric	Pediatric	0.545
Fold3.Rep01	Rep01	Fold3	SVM	Pediatric	Pediatric	0.535
Fold3.Rep01	Rep01	Fold3	SVM	Pediatric	Adult	0.428
Fold3.Rep01	Rep01	Fold3	SVM	Pediatric	Pediatric	0.554
Fold3.Rep01	Rep01	Fold3	SVM	Adult	Adult	0.121
Fold3.Rep01	Rep01	Fold3	SVM	Adult	Adult	0.298
Fold3.Rep01	Rep01	Fold3	SVM	Adult	Adult	0.268
Fold3.Rep01	Rep01	Fold3	SVM	Adult	Adult	0.331
Fold4.Rep01	Rep01	Fold4	RM	Adult	Adult	0.030
Fold4.Rep01	Rep01	Fold4	RM	Adult	Adult	0.124
Fold4.Rep01	Rep01	Fold4	RM	Adult	Adult	0.048
Fold4.Rep01	Rep01	Fold4	RM	Adult	Adult	0.032
Fold4.Rep01	Rep01	Fold4	RM	Adult	Adult	0.080
Fold4.Rep01	Rep01	Fold4	RM	Pediatric	Pediatric	0.816
Fold4.Rep01	Rep01	Fold4	RM	Pediatric	Pediatric	0.756
Fold4.Rep01	Rep01	Fold4	RM	Pediatric	Pediatric	0.826
Fold4.Rep01	Rep01	Fold4	RM	Pediatric	Pediatric	0.784
Fold4.Rep01	Rep01	Fold4	RM	Pediatric	Pediatric	0.756
Fold4.Rep01	Rep01	Fold4	RM	Adult	Adult	0.270
Fold4.Rep01	Rep01	Fold4	RM	Adult	Adult	0.482
Fold4.Rep01	Rep01	Fold4	RM	Adult	Adult	0.118
Fold4.Rep01	Rep01	Fold4	RM	Adult	Adult	0.246
Fold4.Rep01	Rep01	Fold4	GBM	Adult	Adult	0.000
Fold4.Rep01	Rep01	Fold4	GBM	Adult	Adult	0.001
Fold4.Rep01	Rep01	Fold4	GBM	Adult	Adult	0.000
Fold4.Rep01	Rep01	Fold4	GBM	Adult	Adult	0.000
Fold4.Rep01	Rep01	Fold4	GBM	Adult	Adult	0.000
Fold4.Rep01	Rep01	Fold4	GBM	Pediatric	Pediatric	0.999
Fold4.Rep01	Rep01	Fold4	GBM	Pediatric	Pediatric	0.997
Fold4.Rep01	Rep01	Fold4	GBM	Pediatric	Pediatric	0.993
Fold4.Rep01	Rep01	Fold4	GBM	Pediatric	Pediatric	0.999
Fold4.Rep01	Rep01	Fold4	GBM	Pediatric	Pediatric	0.992
Fold4.Rep01	Rep01	Fold4	GBM	Adult	Adult	0.001
Fold4.Rep01	Rep01	Fold4	GBM	Adult	Adult	0.331
Fold4.Rep01	Rep01	Fold4	GBM	Adult	Adult	0.000
Fold4.Rep01	Rep01	Fold4	GBM	Adult	Adult	0.003
Fold4.Rep01	Rep01	Fold4	SVM	Adult	Adult	0.022
Fold4.Rep01	Rep01	Fold4	SVM	Adult	Adult	0.029
Fold4.Rep01	Rep01	Fold4	SVM	Adult	Adult	0.005
Fold4.Rep01	Rep01	Fold4	SVM	Adult	Adult	0.045
Fold4.Rep01	Rep01	Fold4	SVM	Adult	Adult	0.000
Fold4.Rep01	Rep01	Fold4	SVM	Pediatric	Adult	0.360
Fold4.Rep01	Rep01	Fold4	SVM	Pediatric	Pediatric	0.942
Fold4.Rep01	Rep01	Fold4	SVM	Pediatric	Pediatric	0.592
Fold4.Rep01	Rep01	Fold4	SVM	Pediatric	Pediatric	0.854
Fold4.Rep01	Rep01	Fold4	SVM	Pediatric	Adult	0.356
Fold4.Rep01	Rep01	Fold4	SVM	Adult	Adult	0.000
Fold4.Rep01	Rep01	Fold4	SVM	Adult	Adult	0.468
Fold4.Rep01	Rep01	Fold4	SVM	Adult	Adult	0.045
Fold4.Rep01	Rep01	Fold4	SVM	Adult	Adult	0.008
Fold5.Rep01	Rep01	Fold5	RM	Adult	Adult	0.020
Fold5.Rep01	Rep01	Fold5	RM	Adult	Adult	0.042
Fold5.Rep01	Rep01	Fold5	RM	Adult	Adult	0.104
Fold5.Rep01	Rep01	Fold5	RM	Adult	Adult	0.046
Fold5.Rep01	Rep01	Fold5	RM	Adult	Adult	0.210
Fold5.Rep01	Rep01	Fold5	RM	Adult	Adult	0.282
Fold5.Rep01	Rep01	Fold5	RM	Adult	Adult	0.174
Fold5.Rep01	Rep01	Fold5	RM	Adult	Adult	0.234

Fold5.Rep01	Rep01	Fold5	RM	Adult	Adult	0.238
Fold5.Rep01	Rep01	Fold5	RM	Pediatric	Pediatric	0.750
Fold5.Rep01	Rep01	Fold5	RM	Pediatric	Pediatric	0.656
Fold5.Rep01	Rep01	Fold5	RM	Pediatric	Pediatric	0.686
Fold5.Rep01	Rep01	Fold5	RM	Pediatric	Pediatric	0.736
Fold5.Rep01	Rep01	Fold5	RM	Adult	Adult	0.160
Fold5.Rep01	Rep01	Fold5	GBM	Adult	Adult	0.010
Fold5.Rep01	Rep01	Fold5	GBM	Adult	Adult	0.010
Fold5.Rep01	Rep01	Fold5	GBM	Adult	Adult	0.004
Fold5.Rep01	Rep01	Fold5	GBM	Adult	Adult	0.010
Fold5.Rep01	Rep01	Fold5	GBM	Adult	Adult	0.066
Fold5.Rep01	Rep01	Fold5	GBM	Adult	Adult	0.041
Fold5.Rep01	Rep01	Fold5	GBM	Adult	Adult	0.013
Fold5.Rep01	Rep01	Fold5	GBM	Adult	Adult	0.004
Fold5.Rep01	Rep01	Fold5	GBM	Adult	Adult	0.009
Fold5.Rep01	Rep01	Fold5	GBM	Pediatric	Pediatric	0.956
Fold5.Rep01	Rep01	Fold5	GBM	Pediatric	Pediatric	0.965
Fold5.Rep01	Rep01	Fold5	GBM	Pediatric	Pediatric	0.839
Fold5.Rep01	Rep01	Fold5	GBM	Pediatric	Pediatric	0.962
Fold5.Rep01	Rep01	Fold5	GBM	Adult	Adult	0.042
Fold5.Rep01	Rep01	Fold5	SVM	Adult	Adult	0.024
Fold5.Rep01	Rep01	Fold5	SVM	Adult	Adult	0.017
Fold5.Rep01	Rep01	Fold5	SVM	Adult	Adult	0.031
Fold5.Rep01	Rep01	Fold5	SVM	Adult	Adult	0.062
Fold5.Rep01	Rep01	Fold5	SVM	Adult	Pediatric	0.523
Fold5.Rep01	Rep01	Fold5	SVM	Adult	Adult	0.170
Fold5.Rep01	Rep01	Fold5	SVM	Adult	Adult	0.000
Fold5.Rep01	Rep01	Fold5	SVM	Adult	Adult	0.193
Fold5.Rep01	Rep01	Fold5	SVM	Adult	Adult	0.346
Fold5.Rep01	Rep01	Fold5	SVM	Pediatric	Adult	0.000
Fold5.Rep01	Rep01	Fold5	SVM	Pediatric	Adult	0.369
Fold5.Rep01	Rep01	Fold5	SVM	Pediatric	Pediatric	0.807
Fold5.Rep01	Rep01	Fold5	SVM	Pediatric	Pediatric	0.920
Fold5.Rep01	Rep01	Fold5	SVM	Adult	Adult	0.000
Fold1.Rep02	Rep02	Fold1	RM	Adult	Adult	0.174
Fold1.Rep02	Rep02	Fold1	RM	Adult	Adult	0.070
Fold1.Rep02	Rep02	Fold1	RM	Adult	Adult	0.020
Fold1.Rep02	Rep02	Fold1	RM	Adult	Adult	0.364
Fold1.Rep02	Rep02	Fold1	RM	Adult	Adult	0.180
Fold1.Rep02	Rep02	Fold1	RM	Adult	Adult	0.334
Fold1.Rep02	Rep02	Fold1	RM	Adult	Adult	0.228
Fold1.Rep02	Rep02	Fold1	RM	Pediatric	Pediatric	0.874
Fold1.Rep02	Rep02	Fold1	RM	Pediatric	Pediatric	0.880
Fold1.Rep02	Rep02	Fold1	RM	Pediatric	Pediatric	0.844
Fold1.Rep02	Rep02	Fold1	RM	Pediatric	Pediatric	0.720
Fold1.Rep02	Rep02	Fold1	RM	Pediatric	Pediatric	0.884
Fold1.Rep02	Rep02	Fold1	RM	Adult	Adult	0.206
Fold1.Rep02	Rep02	Fold1	RM	Adult	Adult	0.190
Fold1.Rep02	Rep02	Fold1	GBM	Adult	Adult	0.001
Fold1.Rep02	Rep02	Fold1	GBM	Adult	Adult	0.001
Fold1.Rep02	Rep02	Fold1	GBM	Adult	Adult	0.001
Fold1.Rep02	Rep02	Fold1	GBM	Adult	Adult	0.007
Fold1.Rep02	Rep02	Fold1	GBM	Adult	Adult	0.000
Fold1.Rep02	Rep02	Fold1	GBM	Adult	Adult	0.001
Fold1.Rep02	Rep02	Fold1	GBM	Adult	Adult	0.000
Fold1.Rep02	Rep02	Fold1	GBM	Pediatric	Pediatric	0.999
Fold1.Rep02	Rep02	Fold1	GBM	Pediatric	Pediatric	0.998
Fold1.Rep02	Rep02	Fold1	GBM	Pediatric	Pediatric	0.999
Fold1.Rep02	Rep02	Fold1	GBM	Pediatric	Pediatric	0.997

Fold1.Rep02	Rep02	Fold1	GBM	Pediatric	Pediatric	0.999
Fold1.Rep02	Rep02	Fold1	GBM	Adult	Adult	0.000
Fold1.Rep02	Rep02	Fold1	GBM	Adult	Adult	0.000
Fold1.Rep02	Rep02	Fold1	SVM	Adult	Adult	0.000
Fold1.Rep02	Rep02	Fold1	SVM	Adult	Adult	0.090
Fold1.Rep02	Rep02	Fold1	SVM	Adult	Adult	0.075
Fold1.Rep02	Rep02	Fold1	SVM	Adult	Adult	0.285
Fold1.Rep02	Rep02	Fold1	SVM	Adult	Adult	0.000
Fold1.Rep02	Rep02	Fold1	SVM	Adult	Adult	0.001
Fold1.Rep02	Rep02	Fold1	SVM	Adult	Pediatric	1.000
Fold1.Rep02	Rep02	Fold1	SVM	Pediatric	Pediatric	0.934
Fold1.Rep02	Rep02	Fold1	SVM	Pediatric	Pediatric	0.682
Fold1.Rep02	Rep02	Fold1	SVM	Pediatric	Adult	0.000
Fold1.Rep02	Rep02	Fold1	SVM	Pediatric	Adult	0.022
Fold1.Rep02	Rep02	Fold1	SVM	Pediatric	Pediatric	0.805
Fold1.Rep02	Rep02	Fold1	SVM	Adult	Adult	0.000
Fold1.Rep02	Rep02	Fold1	SVM	Adult	Adult	0.024
Fold2.Rep02	Rep02	Fold2	RM	Adult	Adult	0.038
Fold2.Rep02	Rep02	Fold2	RM	Adult	Adult	0.038
Fold2.Rep02	Rep02	Fold2	RM	Adult	Adult	0.082
Fold2.Rep02	Rep02	Fold2	RM	Adult	Adult	0.028
Fold2.Rep02	Rep02	Fold2	RM	Adult	Adult	0.070
Fold2.Rep02	Rep02	Fold2	RM	Adult	Adult	0.292
Fold2.Rep02	Rep02	Fold2	RM	Adult	Adult	0.114
Fold2.Rep02	Rep02	Fold2	RM	Pediatric	Pediatric	0.694
Fold2.Rep02	Rep02	Fold2	RM	Pediatric	Pediatric	0.618
Fold2.Rep02	Rep02	Fold2	RM	Pediatric	Pediatric	0.794
Fold2.Rep02	Rep02	Fold2	RM	Pediatric	Pediatric	0.576
Fold2.Rep02	Rep02	Fold2	RM	Pediatric	Pediatric	0.544
Fold2.Rep02	Rep02	Fold2	RM	Adult	Adult	0.178
Fold2.Rep02	Rep02	Fold2	RM	Adult	Adult	0.062
Fold2.Rep02	Rep02	Fold2	GBM	Adult	Adult	0.014
Fold2.Rep02	Rep02	Fold2	GBM	Adult	Adult	0.014
Fold2.Rep02	Rep02	Fold2	GBM	Adult	Adult	0.006
Fold2.Rep02	Rep02	Fold2	GBM	Adult	Adult	0.003
Fold2.Rep02	Rep02	Fold2	GBM	Adult	Adult	0.017
Fold2.Rep02	Rep02	Fold2	GBM	Adult	Adult	0.036
Fold2.Rep02	Rep02	Fold2	GBM	Adult	Adult	0.011
Fold2.Rep02	Rep02	Fold2	GBM	Pediatric	Pediatric	0.966
Fold2.Rep02	Rep02	Fold2	GBM	Pediatric	Pediatric	0.938
Fold2.Rep02	Rep02	Fold2	GBM	Pediatric	Pediatric	0.963
Fold2.Rep02	Rep02	Fold2	GBM	Pediatric	Pediatric	0.892
Fold2.Rep02	Rep02	Fold2	GBM	Pediatric	Pediatric	0.791
Fold2.Rep02	Rep02	Fold2	GBM	Adult	Adult	0.002
Fold2.Rep02	Rep02	Fold2	GBM	Adult	Adult	0.007
Fold2.Rep02	Rep02	Fold2	SVM	Adult	Adult	0.018
Fold2.Rep02	Rep02	Fold2	SVM	Adult	Adult	0.103
Fold2.Rep02	Rep02	Fold2	SVM	Adult	Adult	0.145
Fold2.Rep02	Rep02	Fold2	SVM	Adult	Adult	0.073
Fold2.Rep02	Rep02	Fold2	SVM	Adult	Adult	0.158
Fold2.Rep02	Rep02	Fold2	SVM	Adult	Adult	0.001
Fold2.Rep02	Rep02	Fold2	SVM	Adult	Pediatric	0.668
Fold2.Rep02	Rep02	Fold2	SVM	Pediatric	Pediatric	0.564
Fold2.Rep02	Rep02	Fold2	SVM	Pediatric	Pediatric	0.556
Fold2.Rep02	Rep02	Fold2	SVM	Pediatric	Pediatric	0.567
Fold2.Rep02	Rep02	Fold2	SVM	Pediatric	Adult	0.426
Fold2.Rep02	Rep02	Fold2	SVM	Pediatric	Adult	0.221
Fold2.Rep02	Rep02	Fold2	SVM	Adult	Adult	0.077
Fold2.Rep02	Rep02	Fold2	SVM	Adult	Adult	0.086

Fold3.Rep02	Rep02	Fold3	RM	Adult	Adult	0.014
Fold3.Rep02	Rep02	Fold3	RM	Adult	Adult	0.022
Fold3.Rep02	Rep02	Fold3	RM	Adult	Adult	0.022
Fold3.Rep02	Rep02	Fold3	RM	Adult	Adult	0.276
Fold3.Rep02	Rep02	Fold3	RM	Adult	Adult	0.180
Fold3.Rep02	Rep02	Fold3	RM	Pediatric	Pediatric	0.878
Fold3.Rep02	Rep02	Fold3	RM	Pediatric	Pediatric	0.816
Fold3.Rep02	Rep02	Fold3	RM	Pediatric	Pediatric	0.742
Fold3.Rep02	Rep02	Fold3	RM	Pediatric	Pediatric	0.672
Fold3.Rep02	Rep02	Fold3	RM	Adult	Adult	0.124
Fold3.Rep02	Rep02	Fold3	RM	Adult	Adult	0.082
Fold3.Rep02	Rep02	Fold3	RM	Adult	Adult	0.404
Fold3.Rep02	Rep02	Fold3	RM	Adult	Adult	0.244
Fold3.Rep02	Rep02	Fold3	GBM	Adult	Adult	0.003
Fold3.Rep02	Rep02	Fold3	GBM	Adult	Adult	0.002
Fold3.Rep02	Rep02	Fold3	GBM	Adult	Adult	0.002
Fold3.Rep02	Rep02	Fold3	GBM	Adult	Adult	0.051
Fold3.Rep02	Rep02	Fold3	GBM	Adult	Adult	0.006
Fold3.Rep02	Rep02	Fold3	GBM	Pediatric	Pediatric	0.980
Fold3.Rep02	Rep02	Fold3	GBM	Pediatric	Pediatric	0.906
Fold3.Rep02	Rep02	Fold3	GBM	Pediatric	Pediatric	0.950
Fold3.Rep02	Rep02	Fold3	GBM	Pediatric	Pediatric	0.953
Fold3.Rep02	Rep02	Fold3	GBM	Adult	Adult	0.004
Fold3.Rep02	Rep02	Fold3	GBM	Adult	Adult	0.003
Fold3.Rep02	Rep02	Fold3	GBM	Adult	Pediatric	0.671
Fold3.Rep02	Rep02	Fold3	GBM	Adult	Adult	0.088
Fold3.Rep02	Rep02	Fold3	SVM	Adult	Adult	0.095
Fold3.Rep02	Rep02	Fold3	SVM	Adult	Adult	0.184
Fold3.Rep02	Rep02	Fold3	SVM	Adult	Adult	0.096
Fold3.Rep02	Rep02	Fold3	SVM	Adult	Adult	0.000
Fold3.Rep02	Rep02	Fold3	SVM	Adult	Adult	0.000
Fold3.Rep02	Rep02	Fold3	SVM	Pediatric	Pediatric	0.547
Fold3.Rep02	Rep02	Fold3	SVM	Pediatric	Pediatric	0.706
Fold3.Rep02	Rep02	Fold3	SVM	Pediatric	Pediatric	0.804
Fold3.Rep02	Rep02	Fold3	SVM	Pediatric	Pediatric	0.548
Fold3.Rep02	Rep02	Fold3	SVM	Adult	Adult	0.329
Fold3.Rep02	Rep02	Fold3	SVM	Adult	Adult	0.306
Fold3.Rep02	Rep02	Fold3	SVM	Adult	Pediatric	0.664
Fold3.Rep02	Rep02	Fold3	SVM	Adult	Adult	0.319
Fold4.Rep02	Rep02	Fold4	RM	Adult	Adult	0.030
Fold4.Rep02	Rep02	Fold4	RM	Adult	Adult	0.038
Fold4.Rep02	Rep02	Fold4	RM	Adult	Adult	0.084
Fold4.Rep02	Rep02	Fold4	RM	Adult	Adult	0.010
Fold4.Rep02	Rep02	Fold4	RM	Adult	Adult	0.118
Fold4.Rep02	Rep02	Fold4	RM	Adult	Adult	0.132
Fold4.Rep02	Rep02	Fold4	RM	Pediatric	Pediatric	0.816
Fold4.Rep02	Rep02	Fold4	RM	Pediatric	Pediatric	0.734
Fold4.Rep02	Rep02	Fold4	RM	Pediatric	Pediatric	0.766
Fold4.Rep02	Rep02	Fold4	RM	Pediatric	Pediatric	0.832
Fold4.Rep02	Rep02	Fold4	RM	Adult	Adult	0.174
Fold4.Rep02	Rep02	Fold4	RM	Adult	Adult	0.114
Fold4.Rep02	Rep02	Fold4	RM	Adult	Adult	0.140
Fold4.Rep02	Rep02	Fold4	RM	Adult	Adult	0.188
Fold4.Rep02	Rep02	Fold4	GBM	Adult	Adult	0.004
Fold4.Rep02	Rep02	Fold4	GBM	Adult	Adult	0.002
Fold4.Rep02	Rep02	Fold4	GBM	Adult	Adult	0.005
Fold4.Rep02	Rep02	Fold4	GBM	Adult	Adult	0.002
Fold4.Rep02	Rep02	Fold4	GBM	Adult	Adult	0.006
Fold4.Rep02	Rep02	Fold4	GBM	Adult	Adult	0.001

Fold4.Rep02	Rep02	Fold4	GBM	Pediatric	Pediatric	0.989
Fold4.Rep02	Rep02	Fold4	GBM	Pediatric	Pediatric	0.988
Fold4.Rep02	Rep02	Fold4	GBM	Pediatric	Pediatric	0.984
Fold4.Rep02	Rep02	Fold4	GBM	Pediatric	Pediatric	0.989
Fold4.Rep02	Rep02	Fold4	GBM	Adult	Adult	0.022
Fold4.Rep02	Rep02	Fold4	GBM	Adult	Adult	0.007
Fold4.Rep02	Rep02	Fold4	GBM	Adult	Adult	0.007
Fold4.Rep02	Rep02	Fold4	GBM	Adult	Adult	0.012
Fold4.Rep02	Rep02	Fold4	SVM	Adult	Adult	0.085
Fold4.Rep02	Rep02	Fold4	SVM	Adult	Adult	0.000
Fold4.Rep02	Rep02	Fold4	SVM	Adult	Adult	0.155
Fold4.Rep02	Rep02	Fold4	SVM	Adult	Adult	0.001
Fold4.Rep02	Rep02	Fold4	SVM	Adult	Adult	0.296
Fold4.Rep02	Rep02	Fold4	SVM	Adult	Adult	0.185
Fold4.Rep02	Rep02	Fold4	SVM	Pediatric	Pediatric	0.738
Fold4.Rep02	Rep02	Fold4	SVM	Pediatric	Adult	0.217
Fold4.Rep02	Rep02	Fold4	SVM	Pediatric	Pediatric	0.632
Fold4.Rep02	Rep02	Fold4	SVM	Pediatric	Pediatric	0.697
Fold4.Rep02	Rep02	Fold4	SVM	Adult	Adult	0.000
Fold4.Rep02	Rep02	Fold4	SVM	Adult	Adult	0.059
Fold4.Rep02	Rep02	Fold4	SVM	Adult	Adult	0.375
Fold4.Rep02	Rep02	Fold4	SVM	Adult	Adult	0.009
Fold5.Rep02	Rep02	Fold5	RM	Adult	Adult	0.168
Fold5.Rep02	Rep02	Fold5	RM	Adult	Adult	0.046
Fold5.Rep02	Rep02	Fold5	RM	Adult	Adult	0.024
Fold5.Rep02	Rep02	Fold5	RM	Adult	Adult	0.170
Fold5.Rep02	Rep02	Fold5	RM	Adult	Adult	0.130
Fold5.Rep02	Rep02	Fold5	RM	Pediatric	Pediatric	0.802
Fold5.Rep02	Rep02	Fold5	RM	Pediatric	Pediatric	0.868
Fold5.Rep02	Rep02	Fold5	RM	Pediatric	Pediatric	0.712
Fold5.Rep02	Rep02	Fold5	RM	Pediatric	Pediatric	0.836
Fold5.Rep02	Rep02	Fold5	RM	Adult	Adult	0.070
Fold5.Rep02	Rep02	Fold5	RM	Adult	Adult	0.060
Fold5.Rep02	Rep02	Fold5	RM	Adult	Adult	0.054
Fold5.Rep02	Rep02	Fold5	RM	Adult	Adult	0.086
Fold5.Rep02	Rep02	Fold5	RM	Adult	Adult	0.110
Fold5.Rep02	Rep02	Fold5	GBM	Adult	Adult	0.009
Fold5.Rep02	Rep02	Fold5	GBM	Adult	Adult	0.009
Fold5.Rep02	Rep02	Fold5	GBM	Adult	Adult	0.009
Fold5.Rep02	Rep02	Fold5	GBM	Adult	Adult	0.088
Fold5.Rep02	Rep02	Fold5	GBM	Adult	Adult	0.008
Fold5.Rep02	Rep02	Fold5	GBM	Pediatric	Pediatric	0.978
Fold5.Rep02	Rep02	Fold5	GBM	Pediatric	Pediatric	0.981
Fold5.Rep02	Rep02	Fold5	GBM	Pediatric	Pediatric	0.932
Fold5.Rep02	Rep02	Fold5	GBM	Pediatric	Pediatric	0.978
Fold5.Rep02	Rep02	Fold5	GBM	Adult	Adult	0.002
Fold5.Rep02	Rep02	Fold5	GBM	Adult	Adult	0.002
Fold5.Rep02	Rep02	Fold5	GBM	Adult	Adult	0.002
Fold5.Rep02	Rep02	Fold5	GBM	Adult	Adult	0.007
Fold5.Rep02	Rep02	Fold5	GBM	Adult	Adult	0.002
Fold5.Rep02	Rep02	Fold5	SVM	Adult	Pediatric	1.000
Fold5.Rep02	Rep02	Fold5	SVM	Adult	Adult	0.178
Fold5.Rep02	Rep02	Fold5	SVM	Adult	Adult	0.060
Fold5.Rep02	Rep02	Fold5	SVM	Adult	Pediatric	0.897
Fold5.Rep02	Rep02	Fold5	SVM	Adult	Adult	0.000
Fold5.Rep02	Rep02	Fold5	SVM	Pediatric	Pediatric	0.905
Fold5.Rep02	Rep02	Fold5	SVM	Pediatric	Pediatric	0.535
Fold5.Rep02	Rep02	Fold5	SVM	Pediatric	Adult	0.097
Fold5.Rep02	Rep02	Fold5	SVM	Pediatric	Adult	0.469

Fold5.Rep02	Rep02	Fold5	SVM	Adult	Adult	0.000
Fold5.Rep02	Rep02	Fold5	SVM	Adult	Adult	0.005
Fold5.Rep02	Rep02	Fold5	SVM	Adult	Adult	0.014
Fold5.Rep02	Rep02	Fold5	SVM	Adult	Adult	0.006
Fold5.Rep02	Rep02	Fold5	SVM	Adult	Adult	0.016
Fold1.Rep03	Rep03	Fold1	RM	Adult	Adult	0.026
Fold1.Rep03	Rep03	Fold1	RM	Adult	Adult	0.048
Fold1.Rep03	Rep03	Fold1	RM	Adult	Adult	0.166
Fold1.Rep03	Rep03	Fold1	RM	Adult	Adult	0.018
Fold1.Rep03	Rep03	Fold1	RM	Pediatric	Pediatric	0.832
Fold1.Rep03	Rep03	Fold1	RM	Pediatric	Pediatric	0.616
Fold1.Rep03	Rep03	Fold1	RM	Pediatric	Pediatric	0.884
Fold1.Rep03	Rep03	Fold1	RM	Pediatric	Pediatric	0.720
Fold1.Rep03	Rep03	Fold1	RM	Adult	Adult	0.128
Fold1.Rep03	Rep03	Fold1	RM	Adult	Adult	0.048
Fold1.Rep03	Rep03	Fold1	RM	Adult	Adult	0.028
Fold1.Rep03	Rep03	Fold1	RM	Adult	Adult	0.096
Fold1.Rep03	Rep03	Fold1	RM	Adult	Adult	0.124
Fold1.Rep03	Rep03	Fold1	GBM	Adult	Adult	0.007
Fold1.Rep03	Rep03	Fold1	GBM	Adult	Adult	0.007
Fold1.Rep03	Rep03	Fold1	GBM	Adult	Adult	0.007
Fold1.Rep03	Rep03	Fold1	GBM	Adult	Adult	0.002
Fold1.Rep03	Rep03	Fold1	GBM	Pediatric	Pediatric	0.986
Fold1.Rep03	Rep03	Fold1	GBM	Pediatric	Pediatric	0.931
Fold1.Rep03	Rep03	Fold1	GBM	Pediatric	Pediatric	0.986
Fold1.Rep03	Rep03	Fold1	GBM	Pediatric	Pediatric	0.974
Fold1.Rep03	Rep03	Fold1	GBM	Adult	Adult	0.006
Fold1.Rep03	Rep03	Fold1	GBM	Adult	Adult	0.002
Fold1.Rep03	Rep03	Fold1	GBM	Adult	Adult	0.002
Fold1.Rep03	Rep03	Fold1	GBM	Adult	Adult	0.002
Fold1.Rep03	Rep03	Fold1	GBM	Adult	Adult	0.002
Fold1.Rep03	Rep03	Fold1	SVM	Adult	Adult	0.084
Fold1.Rep03	Rep03	Fold1	SVM	Adult	Adult	0.066
Fold1.Rep03	Rep03	Fold1	SVM	Adult	Pediatric	1.000
Fold1.Rep03	Rep03	Fold1	SVM	Adult	Adult	0.172
Fold1.Rep03	Rep03	Fold1	SVM	Pediatric	Pediatric	0.725
Fold1.Rep03	Rep03	Fold1	SVM	Pediatric	Adult	0.445
Fold1.Rep03	Rep03	Fold1	SVM	Pediatric	Pediatric	0.677
Fold1.Rep03	Rep03	Fold1	SVM	Pediatric	Adult	0.351
Fold1.Rep03	Rep03	Fold1	SVM	Adult	Adult	0.482
Fold1.Rep03	Rep03	Fold1	SVM	Adult	Adult	0.321
Fold1.Rep03	Rep03	Fold1	SVM	Adult	Adult	0.032
Fold1.Rep03	Rep03	Fold1	SVM	Adult	Adult	0.003
Fold1.Rep03	Rep03	Fold1	SVM	Adult	Adult	0.021
Fold2.Rep03	Rep03	Fold2	RM	Adult	Adult	0.022
Fold2.Rep03	Rep03	Fold2	RM	Adult	Adult	0.038
Fold2.Rep03	Rep03	Fold2	RM	Adult	Adult	0.022
Fold2.Rep03	Rep03	Fold2	RM	Adult	Adult	0.006
Fold2.Rep03	Rep03	Fold2	RM	Adult	Adult	0.142
Fold2.Rep03	Rep03	Fold2	RM	Adult	Adult	0.168
Fold2.Rep03	Rep03	Fold2	RM	Adult	Adult	0.164
Fold2.Rep03	Rep03	Fold2	RM	Adult	Adult	0.186
Fold2.Rep03	Rep03	Fold2	RM	Pediatric	Pediatric	0.866
Fold2.Rep03	Rep03	Fold2	RM	Pediatric	Pediatric	0.854
Fold2.Rep03	Rep03	Fold2	RM	Pediatric	Pediatric	0.732
Fold2.Rep03	Rep03	Fold2	RM	Pediatric	Pediatric	0.800
Fold2.Rep03	Rep03	Fold2	RM	Adult	Adult	0.052
Fold2.Rep03	Rep03	Fold2	RM	Adult	Adult	0.156
Fold2.Rep03	Rep03	Fold2	GBM	Adult	Adult	0.008

Fold2.Rep03	Rep03	Fold2	GBM	Adult	Adult	0.008
Fold2.Rep03	Rep03	Fold2	GBM	Adult	Adult	0.003
Fold2.Rep03	Rep03	Fold2	GBM	Adult	Adult	0.002
Fold2.Rep03	Rep03	Fold2	GBM	Adult	Adult	0.005
Fold2.Rep03	Rep03	Fold2	GBM	Adult	Adult	0.008
Fold2.Rep03	Rep03	Fold2	GBM	Adult	Adult	0.001
Fold2.Rep03	Rep03	Fold2	GBM	Adult	Adult	0.009
Fold2.Rep03	Rep03	Fold2	GBM	Pediatric	Pediatric	0.980
Fold2.Rep03	Rep03	Fold2	GBM	Pediatric	Pediatric	0.985
Fold2.Rep03	Rep03	Fold2	GBM	Pediatric	Pediatric	0.972
Fold2.Rep03	Rep03	Fold2	GBM	Pediatric	Pediatric	0.976
Fold2.Rep03	Rep03	Fold2	GBM	Adult	Adult	0.005
Fold2.Rep03	Rep03	Fold2	GBM	Adult	Adult	0.007
Fold2.Rep03	Rep03	Fold2	SVM	Adult	Adult	0.059
Fold2.Rep03	Rep03	Fold2	SVM	Adult	Adult	0.279
Fold2.Rep03	Rep03	Fold2	SVM	Adult	Adult	0.161
Fold2.Rep03	Rep03	Fold2	SVM	Adult	Adult	0.150
Fold2.Rep03	Rep03	Fold2	SVM	Adult	Adult	0.228
Fold2.Rep03	Rep03	Fold2	SVM	Adult	Pediatric	0.568
Fold2.Rep03	Rep03	Fold2	SVM	Adult	Adult	0.241
Fold2.Rep03	Rep03	Fold2	SVM	Adult	Pediatric	1.000
Fold2.Rep03	Rep03	Fold2	SVM	Pediatric	Adult	0.396
Fold2.Rep03	Rep03	Fold2	SVM	Pediatric	Pediatric	0.515
Fold2.Rep03	Rep03	Fold2	SVM	Pediatric	Adult	0.432
Fold2.Rep03	Rep03	Fold2	SVM	Pediatric	Adult	0.470
Fold2.Rep03	Rep03	Fold2	SVM	Adult	Adult	0.157
Fold2.Rep03	Rep03	Fold2	SVM	Adult	Adult	0.088
Fold3.Rep03	Rep03	Fold3	RM	Adult	Adult	0.058
Fold3.Rep03	Rep03	Fold3	RM	Adult	Adult	0.080
Fold3.Rep03	Rep03	Fold3	RM	Adult	Adult	0.190
Fold3.Rep03	Rep03	Fold3	RM	Adult	Adult	0.258
Fold3.Rep03	Rep03	Fold3	RM	Adult	Adult	0.144
Fold3.Rep03	Rep03	Fold3	RM	Pediatric	Pediatric	0.768
Fold3.Rep03	Rep03	Fold3	RM	Pediatric	Pediatric	0.916
Fold3.Rep03	Rep03	Fold3	RM	Pediatric	Pediatric	0.662
Fold3.Rep03	Rep03	Fold3	RM	Pediatric	Pediatric	0.674
Fold3.Rep03	Rep03	Fold3	RM	Pediatric	Pediatric	0.888
Fold3.Rep03	Rep03	Fold3	RM	Adult	Adult	0.240
Fold3.Rep03	Rep03	Fold3	RM	Adult	Adult	0.234
Fold3.Rep03	Rep03	Fold3	RM	Adult	Adult	0.332
Fold3.Rep03	Rep03	Fold3	RM	Adult	Adult	0.128
Fold3.Rep03	Rep03	Fold3	GBM	Adult	Adult	0.000
Fold3.Rep03	Rep03	Fold3	GBM	Adult	Adult	0.000
Fold3.Rep03	Rep03	Fold3	GBM	Adult	Adult	0.001
Fold3.Rep03	Rep03	Fold3	GBM	Adult	Adult	0.005
Fold3.Rep03	Rep03	Fold3	GBM	Adult	Adult	0.000
Fold3.Rep03	Rep03	Fold3	GBM	Pediatric	Pediatric	0.996
Fold3.Rep03	Rep03	Fold3	GBM	Pediatric	Pediatric	0.999
Fold3.Rep03	Rep03	Fold3	GBM	Pediatric	Pediatric	0.948
Fold3.Rep03	Rep03	Fold3	GBM	Pediatric	Pediatric	0.991
Fold3.Rep03	Rep03	Fold3	GBM	Pediatric	Pediatric	0.999
Fold3.Rep03	Rep03	Fold3	GBM	Adult	Adult	0.002
Fold3.Rep03	Rep03	Fold3	GBM	Adult	Adult	0.000
Fold3.Rep03	Rep03	Fold3	GBM	Adult	Adult	0.010
Fold3.Rep03	Rep03	Fold3	GBM	Adult	Adult	0.002
Fold3.Rep03	Rep03	Fold3	SVM	Adult	Adult	0.068
Fold3.Rep03	Rep03	Fold3	SVM	Adult	Adult	0.093
Fold3.Rep03	Rep03	Fold3	SVM	Adult	Adult	0.000
Fold3.Rep03	Rep03	Fold3	SVM	Adult	Adult	0.000

Fold3.Rep03	Rep03	Fold3	SVM	Adult	Adult	0.000
Fold3.Rep03	Rep03	Fold3	SVM	Pediatric	Pediatric	0.774
Fold3.Rep03	Rep03	Fold3	SVM	Pediatric	Pediatric	0.653
Fold3.Rep03	Rep03	Fold3	SVM	Pediatric	Pediatric	0.571
Fold3.Rep03	Rep03	Fold3	SVM	Pediatric	Pediatric	0.746
Fold3.Rep03	Rep03	Fold3	SVM	Pediatric	Pediatric	0.738
Fold3.Rep03	Rep03	Fold3	SVM	Adult	Adult	0.000
Fold3.Rep03	Rep03	Fold3	SVM	Adult	Adult	0.011
Fold3.Rep03	Rep03	Fold3	SVM	Adult	Adult	0.053
Fold3.Rep03	Rep03	Fold3	SVM	Adult	Adult	0.295
Fold4.Rep03	Rep03	Fold4	RM	Adult	Adult	0.018
Fold4.Rep03	Rep03	Fold4	RM	Adult	Adult	0.018
Fold4.Rep03	Rep03	Fold4	RM	Adult	Adult	0.036
Fold4.Rep03	Rep03	Fold4	RM	Adult	Adult	0.030
Fold4.Rep03	Rep03	Fold4	RM	Adult	Adult	0.206
Fold4.Rep03	Rep03	Fold4	RM	Adult	Adult	0.192
Fold4.Rep03	Rep03	Fold4	RM	Adult	Adult	0.274
Fold4.Rep03	Rep03	Fold4	RM	Pediatric	Pediatric	0.810
Fold4.Rep03	Rep03	Fold4	RM	Pediatric	Pediatric	0.806
Fold4.Rep03	Rep03	Fold4	RM	Pediatric	Pediatric	0.898
Fold4.Rep03	Rep03	Fold4	RM	Pediatric	Pediatric	0.650
Fold4.Rep03	Rep03	Fold4	RM	Pediatric	Pediatric	0.796
Fold4.Rep03	Rep03	Fold4	RM	Adult	Adult	0.226
Fold4.Rep03	Rep03	Fold4	RM	Adult	Adult	0.134
Fold4.Rep03	Rep03	Fold4	RM	Adult	Adult	0.112
Fold4.Rep03	Rep03	Fold4	GBM	Adult	Adult	0.015
Fold4.Rep03	Rep03	Fold4	GBM	Adult	Adult	0.015
Fold4.Rep03	Rep03	Fold4	GBM	Adult	Adult	0.004
Fold4.Rep03	Rep03	Fold4	GBM	Adult	Adult	0.002
Fold4.Rep03	Rep03	Fold4	GBM	Adult	Adult	0.091
Fold4.Rep03	Rep03	Fold4	GBM	Adult	Adult	0.009
Fold4.Rep03	Rep03	Fold4	GBM	Adult	Adult	0.014
Fold4.Rep03	Rep03	Fold4	GBM	Pediatric	Pediatric	0.976
Fold4.Rep03	Rep03	Fold4	GBM	Pediatric	Pediatric	0.954
Fold4.Rep03	Rep03	Fold4	GBM	Pediatric	Pediatric	0.978
Fold4.Rep03	Rep03	Fold4	GBM	Pediatric	Pediatric	0.849
Fold4.Rep03	Rep03	Fold4	GBM	Pediatric	Pediatric	0.933
Fold4.Rep03	Rep03	Fold4	GBM	Adult	Adult	0.012
Fold4.Rep03	Rep03	Fold4	GBM	Adult	Adult	0.008
Fold4.Rep03	Rep03	Fold4	GBM	Adult	Adult	0.007
Fold4.Rep03	Rep03	Fold4	SVM	Adult	Adult	0.004
Fold4.Rep03	Rep03	Fold4	SVM	Adult	Adult	0.008
Fold4.Rep03	Rep03	Fold4	SVM	Adult	Adult	0.012
Fold4.Rep03	Rep03	Fold4	SVM	Adult	Adult	0.004
Fold4.Rep03	Rep03	Fold4	SVM	Adult	Pediatric	0.597
Fold4.Rep03	Rep03	Fold4	SVM	Adult	Adult	0.001
Fold4.Rep03	Rep03	Fold4	SVM	Adult	Pediatric	0.878
Fold4.Rep03	Rep03	Fold4	SVM	Pediatric	Pediatric	1.000
Fold4.Rep03	Rep03	Fold4	SVM	Pediatric	Pediatric	0.867
Fold4.Rep03	Rep03	Fold4	SVM	Pediatric	Pediatric	0.917
Fold4.Rep03	Rep03	Fold4	SVM	Pediatric	Adult	0.031
Fold4.Rep03	Rep03	Fold4	SVM	Pediatric	Pediatric	0.928
Fold4.Rep03	Rep03	Fold4	SVM	Adult	Adult	0.000
Fold4.Rep03	Rep03	Fold4	SVM	Adult	Adult	0.074
Fold4.Rep03	Rep03	Fold4	SVM	Adult	Adult	0.196
Fold5.Rep03	Rep03	Fold5	RM	Adult	Adult	0.190
Fold5.Rep03	Rep03	Fold5	RM	Adult	Adult	0.152
Fold5.Rep03	Rep03	Fold5	RM	Adult	Adult	0.036
Fold5.Rep03	Rep03	Fold5	RM	Adult	Adult	0.046

Fold5.Rep03	Rep03	Fold5	RM	Adult	Adult	0.358
Fold5.Rep03	Rep03	Fold5	RM	Adult	Adult	0.286
Fold5.Rep03	Rep03	Fold5	RM	Pediatric	Pediatric	0.800
Fold5.Rep03	Rep03	Fold5	RM	Pediatric	Pediatric	0.730
Fold5.Rep03	Rep03	Fold5	RM	Pediatric	Pediatric	0.768
Fold5.Rep03	Rep03	Fold5	RM	Pediatric	Pediatric	0.720
Fold5.Rep03	Rep03	Fold5	RM	Adult	Adult	0.048
Fold5.Rep03	Rep03	Fold5	RM	Adult	Adult	0.412
Fold5.Rep03	Rep03	Fold5	RM	Adult	Adult	0.028
Fold5.Rep03	Rep03	Fold5	GBM	Adult	Adult	0.005
Fold5.Rep03	Rep03	Fold5	GBM	Adult	Adult	0.003
Fold5.Rep03	Rep03	Fold5	GBM	Adult	Adult	0.004
Fold5.Rep03	Rep03	Fold5	GBM	Adult	Adult	0.004
Fold5.Rep03	Rep03	Fold5	GBM	Adult	Adult	0.010
Fold5.Rep03	Rep03	Fold5	GBM	Adult	Adult	0.006
Fold5.Rep03	Rep03	Fold5	GBM	Pediatric	Pediatric	0.992
Fold5.Rep03	Rep03	Fold5	GBM	Pediatric	Pediatric	0.982
Fold5.Rep03	Rep03	Fold5	GBM	Pediatric	Pediatric	0.992
Fold5.Rep03	Rep03	Fold5	GBM	Pediatric	Pediatric	0.990
Fold5.Rep03	Rep03	Fold5	GBM	Adult	Adult	0.002
Fold5.Rep03	Rep03	Fold5	GBM	Adult	Adult	0.494
Fold5.Rep03	Rep03	Fold5	GBM	Adult	Adult	0.002
Fold5.Rep03	Rep03	Fold5	SVM	Adult	Adult	0.095
Fold5.Rep03	Rep03	Fold5	SVM	Adult	Adult	0.059
Fold5.Rep03	Rep03	Fold5	SVM	Adult	Adult	0.060
Fold5.Rep03	Rep03	Fold5	SVM	Adult	Adult	0.073
Fold5.Rep03	Rep03	Fold5	SVM	Adult	Pediatric	0.883
Fold5.Rep03	Rep03	Fold5	SVM	Adult	Pediatric	0.977
Fold5.Rep03	Rep03	Fold5	SVM	Pediatric	Adult	0.291
Fold5.Rep03	Rep03	Fold5	SVM	Pediatric	Adult	0.300
Fold5.Rep03	Rep03	Fold5	SVM	Pediatric	Adult	0.338
Fold5.Rep03	Rep03	Fold5	SVM	Pediatric	Pediatric	0.989
Fold5.Rep03	Rep03	Fold5	SVM	Adult	Adult	0.000
Fold5.Rep03	Rep03	Fold5	SVM	Adult	Adult	0.348
Fold5.Rep03	Rep03	Fold5	SVM	Adult	Adult	0.002
Fold1.Rep04	Rep04	Fold1	RM	Adult	Adult	0.026
Fold1.Rep04	Rep04	Fold1	RM	Adult	Adult	0.116
Fold1.Rep04	Rep04	Fold1	RM	Adult	Adult	0.014
Fold1.Rep04	Rep04	Fold1	RM	Adult	Adult	0.026
Fold1.Rep04	Rep04	Fold1	RM	Adult	Adult	0.064
Fold1.Rep04	Rep04	Fold1	RM	Adult	Adult	0.132
Fold1.Rep04	Rep04	Fold1	RM	Adult	Adult	0.210
Fold1.Rep04	Rep04	Fold1	RM	Pediatric	Pediatric	0.840
Fold1.Rep04	Rep04	Fold1	RM	Pediatric	Pediatric	0.782
Fold1.Rep04	Rep04	Fold1	RM	Pediatric	Pediatric	0.632
Fold1.Rep04	Rep04	Fold1	RM	Pediatric	Pediatric	0.894
Fold1.Rep04	Rep04	Fold1	RM	Adult	Adult	0.154
Fold1.Rep04	Rep04	Fold1	RM	Adult	Adult	0.082
Fold1.Rep04	Rep04	Fold1	GBM	Adult	Adult	0.008
Fold1.Rep04	Rep04	Fold1	GBM	Adult	Adult	0.004
Fold1.Rep04	Rep04	Fold1	GBM	Adult	Adult	0.002
Fold1.Rep04	Rep04	Fold1	GBM	Adult	Adult	0.008
Fold1.Rep04	Rep04	Fold1	GBM	Adult	Adult	0.008
Fold1.Rep04	Rep04	Fold1	GBM	Adult	Adult	0.003
Fold1.Rep04	Rep04	Fold1	GBM	Adult	Adult	0.004
Fold1.Rep04	Rep04	Fold1	GBM	Pediatric	Pediatric	0.975
Fold1.Rep04	Rep04	Fold1	GBM	Pediatric	Pediatric	0.983
Fold1.Rep04	Rep04	Fold1	GBM	Pediatric	Pediatric	0.918
Fold1.Rep04	Rep04	Fold1	GBM	Pediatric	Pediatric	0.988

Fold1.Rep04	Rep04	Fold1	GBM	Adult	Adult	0.002
Fold1.Rep04	Rep04	Fold1	GBM	Adult	Adult	0.009
Fold1.Rep04	Rep04	Fold1	SVM	Adult	Adult	0.051
Fold1.Rep04	Rep04	Fold1	SVM	Adult	Adult	0.158
Fold1.Rep04	Rep04	Fold1	SVM	Adult	Adult	0.049
Fold1.Rep04	Rep04	Fold1	SVM	Adult	Adult	0.100
Fold1.Rep04	Rep04	Fold1	SVM	Adult	Adult	0.125
Fold1.Rep04	Rep04	Fold1	SVM	Adult	Pediatric	0.750
Fold1.Rep04	Rep04	Fold1	SVM	Adult	Adult	0.000
Fold1.Rep04	Rep04	Fold1	SVM	Pediatric	Pediatric	0.671
Fold1.Rep04	Rep04	Fold1	SVM	Pediatric	Pediatric	0.601
Fold1.Rep04	Rep04	Fold1	SVM	Pediatric	Adult	0.455
Fold1.Rep04	Rep04	Fold1	SVM	Pediatric	Pediatric	0.630
Fold1.Rep04	Rep04	Fold1	SVM	Adult	Adult	0.034
Fold1.Rep04	Rep04	Fold1	SVM	Adult	Adult	0.034
Fold2.Rep04	Rep04	Fold2	RM	Adult	Adult	0.030
Fold2.Rep04	Rep04	Fold2	RM	Adult	Adult	0.130
Fold2.Rep04	Rep04	Fold2	RM	Adult	Adult	0.336
Fold2.Rep04	Rep04	Fold2	RM	Adult	Adult	0.272
Fold2.Rep04	Rep04	Fold2	RM	Adult	Adult	0.170
Fold2.Rep04	Rep04	Fold2	RM	Pediatric	Pediatric	0.810
Fold2.Rep04	Rep04	Fold2	RM	Pediatric	Pediatric	0.826
Fold2.Rep04	Rep04	Fold2	RM	Pediatric	Pediatric	0.648
Fold2.Rep04	Rep04	Fold2	RM	Pediatric	Pediatric	0.712
Fold2.Rep04	Rep04	Fold2	RM	Adult	Adult	0.126
Fold2.Rep04	Rep04	Fold2	RM	Adult	Adult	0.080
Fold2.Rep04	Rep04	Fold2	RM	Adult	Adult	0.056
Fold2.Rep04	Rep04	Fold2	RM	Adult	Adult	0.092
Fold2.Rep04	Rep04	Fold2	GBM	Adult	Adult	0.002
Fold2.Rep04	Rep04	Fold2	GBM	Adult	Adult	0.004
Fold2.Rep04	Rep04	Fold2	GBM	Adult	Adult	0.006
Fold2.Rep04	Rep04	Fold2	GBM	Adult	Adult	0.008
Fold2.Rep04	Rep04	Fold2	GBM	Adult	Adult	0.004
Fold2.Rep04	Rep04	Fold2	GBM	Pediatric	Pediatric	0.987
Fold2.Rep04	Rep04	Fold2	GBM	Pediatric	Pediatric	0.988
Fold2.Rep04	Rep04	Fold2	GBM	Pediatric	Pediatric	0.960
Fold2.Rep04	Rep04	Fold2	GBM	Pediatric	Pediatric	0.988
Fold2.Rep04	Rep04	Fold2	GBM	Adult	Adult	0.004
Fold2.Rep04	Rep04	Fold2	GBM	Adult	Adult	0.004
Fold2.Rep04	Rep04	Fold2	GBM	Adult	Adult	0.001
Fold2.Rep04	Rep04	Fold2	GBM	Adult	Adult	0.002
Fold2.Rep04	Rep04	Fold2	SVM	Adult	Adult	0.000
Fold2.Rep04	Rep04	Fold2	SVM	Adult	Pediatric	0.556
Fold2.Rep04	Rep04	Fold2	SVM	Adult	Adult	0.409
Fold2.Rep04	Rep04	Fold2	SVM	Adult	Pediatric	0.580
Fold2.Rep04	Rep04	Fold2	SVM	Adult	Adult	0.000
Fold2.Rep04	Rep04	Fold2	SVM	Pediatric	Adult	0.374
Fold2.Rep04	Rep04	Fold2	SVM	Pediatric	Pediatric	0.636
Fold2.Rep04	Rep04	Fold2	SVM	Pediatric	Pediatric	0.734
Fold2.Rep04	Rep04	Fold2	SVM	Pediatric	Adult	0.259
Fold2.Rep04	Rep04	Fold2	SVM	Adult	Pediatric	0.517
Fold2.Rep04	Rep04	Fold2	SVM	Adult	Adult	0.274
Fold2.Rep04	Rep04	Fold2	SVM	Adult	Adult	0.153
Fold2.Rep04	Rep04	Fold2	SVM	Adult	Adult	0.198
Fold3.Rep04	Rep04	Fold3	RM	Adult	Adult	0.014
Fold3.Rep04	Rep04	Fold3	RM	Adult	Adult	0.166
Fold3.Rep04	Rep04	Fold3	RM	Adult	Adult	0.054
Fold3.Rep04	Rep04	Fold3	RM	Adult	Adult	0.020
Fold3.Rep04	Rep04	Fold3	RM	Adult	Adult	0.156

Fold3.Rep04	Rep04	Fold3	RM	Adult	Adult	0.228
Fold3.Rep04	Rep04	Fold3	RM	Adult	Adult	0.128
Fold3.Rep04	Rep04	Fold3	RM	Pediatric	Pediatric	0.864
Fold3.Rep04	Rep04	Fold3	RM	Pediatric	Pediatric	0.662
Fold3.Rep04	Rep04	Fold3	RM	Pediatric	Pediatric	0.844
Fold3.Rep04	Rep04	Fold3	RM	Pediatric	Pediatric	0.724
Fold3.Rep04	Rep04	Fold3	RM	Adult	Adult	0.284
Fold3.Rep04	Rep04	Fold3	RM	Adult	Adult	0.200
Fold3.Rep04	Rep04	Fold3	RM	Adult	Adult	0.154
Fold3.Rep04	Rep04	Fold3	GBM	Adult	Adult	0.006
Fold3.Rep04	Rep04	Fold3	GBM	Adult	Adult	0.013
Fold3.Rep04	Rep04	Fold3	GBM	Adult	Adult	0.006
Fold3.Rep04	Rep04	Fold3	GBM	Adult	Adult	0.003
Fold3.Rep04	Rep04	Fold3	GBM	Adult	Adult	0.002
Fold3.Rep04	Rep04	Fold3	GBM	Adult	Adult	0.149
Fold3.Rep04	Rep04	Fold3	GBM	Adult	Adult	0.008
Fold3.Rep04	Rep04	Fold3	GBM	Pediatric	Pediatric	0.980
Fold3.Rep04	Rep04	Fold3	GBM	Pediatric	Pediatric	0.975
Fold3.Rep04	Rep04	Fold3	GBM	Pediatric	Pediatric	0.954
Fold3.Rep04	Rep04	Fold3	GBM	Pediatric	Pediatric	0.954
Fold3.Rep04	Rep04	Fold3	GBM	Adult	Adult	0.063
Fold3.Rep04	Rep04	Fold3	GBM	Adult	Adult	0.005
Fold3.Rep04	Rep04	Fold3	GBM	Adult	Adult	0.025
Fold3.Rep04	Rep04	Fold3	SVM	Adult	Adult	0.011
Fold3.Rep04	Rep04	Fold3	SVM	Adult	Adult	0.079
Fold3.Rep04	Rep04	Fold3	SVM	Adult	Adult	0.114
Fold3.Rep04	Rep04	Fold3	SVM	Adult	Adult	0.065
Fold3.Rep04	Rep04	Fold3	SVM	Adult	Adult	0.149
Fold3.Rep04	Rep04	Fold3	SVM	Adult	Adult	0.443
Fold3.Rep04	Rep04	Fold3	SVM	Adult	Adult	0.000
Fold3.Rep04	Rep04	Fold3	SVM	Pediatric	Pediatric	0.530
Fold3.Rep04	Rep04	Fold3	SVM	Pediatric	Pediatric	0.625
Fold3.Rep04	Rep04	Fold3	SVM	Pediatric	Adult	0.297
Fold3.Rep04	Rep04	Fold3	SVM	Pediatric	Adult	0.080
Fold3.Rep04	Rep04	Fold3	SVM	Adult	Adult	0.060
Fold3.Rep04	Rep04	Fold3	SVM	Adult	Adult	0.003
Fold3.Rep04	Rep04	Fold3	SVM	Adult	Adult	0.009
Fold4.Rep04	Rep04	Fold4	RM	Adult	Adult	0.052
Fold4.Rep04	Rep04	Fold4	RM	Adult	Adult	0.044
Fold4.Rep04	Rep04	Fold4	RM	Adult	Adult	0.104
Fold4.Rep04	Rep04	Fold4	RM	Adult	Adult	0.036
Fold4.Rep04	Rep04	Fold4	RM	Adult	Adult	0.224
Fold4.Rep04	Rep04	Fold4	RM	Pediatric	Pediatric	0.828
Fold4.Rep04	Rep04	Fold4	RM	Pediatric	Pediatric	0.682
Fold4.Rep04	Rep04	Fold4	RM	Pediatric	Pediatric	0.852
Fold4.Rep04	Rep04	Fold4	RM	Pediatric	Pediatric	0.658
Fold4.Rep04	Rep04	Fold4	RM	Pediatric	Pediatric	0.690
Fold4.Rep04	Rep04	Fold4	RM	Adult	Adult	0.246
Fold4.Rep04	Rep04	Fold4	RM	Adult	Adult	0.094
Fold4.Rep04	Rep04	Fold4	RM	Adult	Adult	0.160
Fold4.Rep04	Rep04	Fold4	RM	Adult	Adult	0.146
Fold4.Rep04	Rep04	Fold4	GBM	Adult	Adult	0.001
Fold4.Rep04	Rep04	Fold4	GBM	Adult	Adult	0.001
Fold4.Rep04	Rep04	Fold4	GBM	Adult	Adult	0.000
Fold4.Rep04	Rep04	Fold4	GBM	Adult	Adult	0.000
Fold4.Rep04	Rep04	Fold4	GBM	Adult	Adult	0.014
Fold4.Rep04	Rep04	Fold4	GBM	Pediatric	Pediatric	0.999
Fold4.Rep04	Rep04	Fold4	GBM	Pediatric	Pediatric	0.999
Fold4.Rep04	Rep04	Fold4	GBM	Pediatric	Pediatric	0.999

Fold4.Rep04	Rep04	Fold4	GBM	Pediatric	Pediatric	0.971
Fold4.Rep04	Rep04	Fold4	GBM	Pediatric	Pediatric	0.999
Fold4.Rep04	Rep04	Fold4	GBM	Adult	Adult	0.001
Fold4.Rep04	Rep04	Fold4	GBM	Adult	Adult	0.000
Fold4.Rep04	Rep04	Fold4	GBM	Adult	Adult	0.001
Fold4.Rep04	Rep04	Fold4	GBM	Adult	Adult	0.001
Fold4.Rep04	Rep04	Fold4	SVM	Adult	Adult	0.040
Fold4.Rep04	Rep04	Fold4	SVM	Adult	Adult	0.061
Fold4.Rep04	Rep04	Fold4	SVM	Adult	Adult	0.097
Fold4.Rep04	Rep04	Fold4	SVM	Adult	Adult	0.006
Fold4.Rep04	Rep04	Fold4	SVM	Adult	Adult	0.000
Fold4.Rep04	Rep04	Fold4	SVM	Pediatric	Pediatric	0.736
Fold4.Rep04	Rep04	Fold4	SVM	Pediatric	Adult	0.497
Fold4.Rep04	Rep04	Fold4	SVM	Pediatric	Pediatric	0.766
Fold4.Rep04	Rep04	Fold4	SVM	Pediatric	Pediatric	0.862
Fold4.Rep04	Rep04	Fold4	SVM	Pediatric	Pediatric	0.904
Fold4.Rep04	Rep04	Fold4	SVM	Adult	Adult	0.000
Fold4.Rep04	Rep04	Fold4	SVM	Adult	Adult	0.068
Fold4.Rep04	Rep04	Fold4	SVM	Adult	Adult	0.037
Fold4.Rep04	Rep04	Fold4	SVM	Adult	Adult	0.190
Fold5.Rep04	Rep04	Fold5	RM	Adult	Adult	0.034
Fold5.Rep04	Rep04	Fold5	RM	Adult	Adult	0.104
Fold5.Rep04	Rep04	Fold5	RM	Adult	Adult	0.052
Fold5.Rep04	Rep04	Fold5	RM	Adult	Adult	0.022
Fold5.Rep04	Rep04	Fold5	RM	Adult	Adult	0.114
Fold5.Rep04	Rep04	Fold5	RM	Adult	Adult	0.238
Fold5.Rep04	Rep04	Fold5	RM	Pediatric	Pediatric	0.782
Fold5.Rep04	Rep04	Fold5	RM	Pediatric	Pediatric	0.810
Fold5.Rep04	Rep04	Fold5	RM	Pediatric	Pediatric	0.696
Fold5.Rep04	Rep04	Fold5	RM	Pediatric	Pediatric	0.872
Fold5.Rep04	Rep04	Fold5	RM	Pediatric	Pediatric	0.836
Fold5.Rep04	Rep04	Fold5	RM	Adult	Adult	0.186
Fold5.Rep04	Rep04	Fold5	RM	Adult	Adult	0.060
Fold5.Rep04	Rep04	Fold5	RM	Adult	Adult	0.454
Fold5.Rep04	Rep04	Fold5	RM	Adult	Adult	0.040
Fold5.Rep04	Rep04	Fold5	GBM	Adult	Adult	0.004
Fold5.Rep04	Rep04	Fold5	GBM	Adult	Adult	0.004
Fold5.Rep04	Rep04	Fold5	GBM	Adult	Adult	0.004
Fold5.Rep04	Rep04	Fold5	GBM	Adult	Adult	0.002
Fold5.Rep04	Rep04	Fold5	GBM	Adult	Adult	0.003
Fold5.Rep04	Rep04	Fold5	GBM	Adult	Adult	0.007
Fold5.Rep04	Rep04	Fold5	GBM	Pediatric	Pediatric	0.985
Fold5.Rep04	Rep04	Fold5	GBM	Pediatric	Pediatric	0.979
Fold5.Rep04	Rep04	Fold5	GBM	Pediatric	Pediatric	0.968
Fold5.Rep04	Rep04	Fold5	GBM	Pediatric	Pediatric	0.988
Fold5.Rep04	Rep04	Fold5	GBM	Pediatric	Pediatric	0.963
Fold5.Rep04	Rep04	Fold5	GBM	Adult	Adult	0.030
Fold5.Rep04	Rep04	Fold5	GBM	Adult	Adult	0.002
Fold5.Rep04	Rep04	Fold5	GBM	Adult	Adult	0.264
Fold5.Rep04	Rep04	Fold5	GBM	Adult	Adult	0.002
Fold5.Rep04	Rep04	Fold5	SVM	Adult	Adult	0.082
Fold5.Rep04	Rep04	Fold5	SVM	Adult	Pediatric	1.000
Fold5.Rep04	Rep04	Fold5	SVM	Adult	Adult	0.231
Fold5.Rep04	Rep04	Fold5	SVM	Adult	Adult	0.097
Fold5.Rep04	Rep04	Fold5	SVM	Adult	Adult	0.000
Fold5.Rep04	Rep04	Fold5	SVM	Adult	Adult	0.000
Fold5.Rep04	Rep04	Fold5	SVM	Pediatric	Pediatric	0.974
Fold5.Rep04	Rep04	Fold5	SVM	Pediatric	Pediatric	1.000
Fold5.Rep04	Rep04	Fold5	SVM	Pediatric	Pediatric	0.651

Fold5.Rep04	Rep04	Fold5	SVM	Pediatric	Pediatric	0.589
Fold5.Rep04	Rep04	Fold5	SVM	Pediatric	Pediatric	0.992
Fold5.Rep04	Rep04	Fold5	SVM	Adult	Adult	0.000
Fold5.Rep04	Rep04	Fold5	SVM	Adult	Adult	0.000
Fold5.Rep04	Rep04	Fold5	SVM	Adult	Adult	0.380
Fold5.Rep04	Rep04	Fold5	SVM	Adult	Adult	0.018
Fold1.Rep05	Rep05	Fold1	RM	Adult	Adult	0.026
Fold1.Rep05	Rep05	Fold1	RM	Adult	Adult	0.134
Fold1.Rep05	Rep05	Fold1	RM	Adult	Adult	0.026
Fold1.Rep05	Rep05	Fold1	RM	Adult	Adult	0.060
Fold1.Rep05	Rep05	Fold1	RM	Adult	Adult	0.156
Fold1.Rep05	Rep05	Fold1	RM	Adult	Adult	0.124
Fold1.Rep05	Rep05	Fold1	RM	Pediatric	Pediatric	0.714
Fold1.Rep05	Rep05	Fold1	RM	Pediatric	Pediatric	0.836
Fold1.Rep05	Rep05	Fold1	RM	Pediatric	Pediatric	0.786
Fold1.Rep05	Rep05	Fold1	RM	Pediatric	Pediatric	0.824
Fold1.Rep05	Rep05	Fold1	RM	Pediatric	Pediatric	0.688
Fold1.Rep05	Rep05	Fold1	RM	Adult	Adult	0.038
Fold1.Rep05	Rep05	Fold1	RM	Adult	Adult	0.260
Fold1.Rep05	Rep05	Fold1	RM	Adult	Adult	0.166
Fold1.Rep05	Rep05	Fold1	GBM	Adult	Adult	0.007
Fold1.Rep05	Rep05	Fold1	GBM	Adult	Adult	0.007
Fold1.Rep05	Rep05	Fold1	GBM	Adult	Adult	0.007
Fold1.Rep05	Rep05	Fold1	GBM	Adult	Adult	0.004
Fold1.Rep05	Rep05	Fold1	GBM	Adult	Adult	0.014
Fold1.Rep05	Rep05	Fold1	GBM	Adult	Adult	0.008
Fold1.Rep05	Rep05	Fold1	GBM	Pediatric	Pediatric	0.951
Fold1.Rep05	Rep05	Fold1	GBM	Pediatric	Pediatric	0.983
Fold1.Rep05	Rep05	Fold1	GBM	Pediatric	Pediatric	0.986
Fold1.Rep05	Rep05	Fold1	GBM	Pediatric	Pediatric	0.930
Fold1.Rep05	Rep05	Fold1	GBM	Pediatric	Pediatric	0.910
Fold1.Rep05	Rep05	Fold1	GBM	Adult	Adult	0.002
Fold1.Rep05	Rep05	Fold1	GBM	Adult	Adult	0.108
Fold1.Rep05	Rep05	Fold1	GBM	Adult	Adult	0.006
Fold1.Rep05	Rep05	Fold1	SVM	Adult	Adult	0.007
Fold1.Rep05	Rep05	Fold1	SVM	Adult	Pediatric	1.000
Fold1.Rep05	Rep05	Fold1	SVM	Adult	Adult	0.050
Fold1.Rep05	Rep05	Fold1	SVM	Adult	Adult	0.040
Fold1.Rep05	Rep05	Fold1	SVM	Adult	Pediatric	0.599
Fold1.Rep05	Rep05	Fold1	SVM	Adult	Adult	0.000
Fold1.Rep05	Rep05	Fold1	SVM	Pediatric	Pediatric	0.523
Fold1.Rep05	Rep05	Fold1	SVM	Pediatric	Pediatric	0.563
Fold1.Rep05	Rep05	Fold1	SVM	Pediatric	Pediatric	0.809
Fold1.Rep05	Rep05	Fold1	SVM	Pediatric	Pediatric	0.997
Fold1.Rep05	Rep05	Fold1	SVM	Pediatric	Adult	0.459
Fold1.Rep05	Rep05	Fold1	SVM	Adult	Adult	0.007
Fold1.Rep05	Rep05	Fold1	SVM	Adult	Adult	0.150
Fold1.Rep05	Rep05	Fold1	SVM	Adult	Adult	0.011
Fold2.Rep05	Rep05	Fold2	RM	Adult	Adult	0.020
Fold2.Rep05	Rep05	Fold2	RM	Adult	Adult	0.026
Fold2.Rep05	Rep05	Fold2	RM	Adult	Adult	0.022
Fold2.Rep05	Rep05	Fold2	RM	Adult	Adult	0.018
Fold2.Rep05	Rep05	Fold2	RM	Adult	Adult	0.198
Fold2.Rep05	Rep05	Fold2	RM	Adult	Adult	0.240
Fold2.Rep05	Rep05	Fold2	RM	Pediatric	Pediatric	0.842
Fold2.Rep05	Rep05	Fold2	RM	Pediatric	Pediatric	0.700
Fold2.Rep05	Rep05	Fold2	RM	Pediatric	Pediatric	0.776
Fold2.Rep05	Rep05	Fold2	RM	Pediatric	Pediatric	0.792
Fold2.Rep05	Rep05	Fold2	RM	Pediatric	Pediatric	0.758

Fold2.Rep05	Rep05	Fold2	RM	Adult	Adult	0.172
Fold2.Rep05	Rep05	Fold2	RM	Adult	Adult	0.082
Fold2.Rep05	Rep05	Fold2	RM	Adult	Adult	0.104
Fold2.Rep05	Rep05	Fold2	GBM	Adult	Adult	0.005
Fold2.Rep05	Rep05	Fold2	GBM	Adult	Adult	0.005
Fold2.Rep05	Rep05	Fold2	GBM	Adult	Adult	0.006
Fold2.Rep05	Rep05	Fold2	GBM	Adult	Adult	0.010
Fold2.Rep05	Rep05	Fold2	GBM	Adult	Adult	0.059
Fold2.Rep05	Rep05	Fold2	GBM	Adult	Adult	0.011
Fold2.Rep05	Rep05	Fold2	GBM	Pediatric	Pediatric	0.980
Fold2.Rep05	Rep05	Fold2	GBM	Pediatric	Pediatric	0.957
Fold2.Rep05	Rep05	Fold2	GBM	Pediatric	Pediatric	0.983
Fold2.Rep05	Rep05	Fold2	GBM	Pediatric	Pediatric	0.950
Fold2.Rep05	Rep05	Fold2	GBM	Pediatric	Pediatric	0.972
Fold2.Rep05	Rep05	Fold2	GBM	Adult	Adult	0.031
Fold2.Rep05	Rep05	Fold2	GBM	Adult	Adult	0.003
Fold2.Rep05	Rep05	Fold2	GBM	Adult	Adult	0.009
Fold2.Rep05	Rep05	Fold2	SVM	Adult	Adult	0.092
Fold2.Rep05	Rep05	Fold2	SVM	Adult	Adult	0.047
Fold2.Rep05	Rep05	Fold2	SVM	Adult	Adult	0.086
Fold2.Rep05	Rep05	Fold2	SVM	Adult	Adult	0.102
Fold2.Rep05	Rep05	Fold2	SVM	Adult	Pediatric	0.693
Fold2.Rep05	Rep05	Fold2	SVM	Adult	Pediatric	0.765
Fold2.Rep05	Rep05	Fold2	SVM	Pediatric	Pediatric	0.962
Fold2.Rep05	Rep05	Fold2	SVM	Pediatric	Adult	0.462
Fold2.Rep05	Rep05	Fold2	SVM	Pediatric	Pediatric	0.757
Fold2.Rep05	Rep05	Fold2	SVM	Pediatric	Pediatric	0.644
Fold2.Rep05	Rep05	Fold2	SVM	Pediatric	Pediatric	0.897
Fold2.Rep05	Rep05	Fold2	SVM	Adult	Adult	0.000
Fold2.Rep05	Rep05	Fold2	SVM	Adult	Adult	0.240
Fold2.Rep05	Rep05	Fold2	SVM	Adult	Adult	0.124
Fold3.Rep05	Rep05	Fold3	RM	Adult	Adult	0.052
Fold3.Rep05	Rep05	Fold3	RM	Adult	Adult	0.028
Fold3.Rep05	Rep05	Fold3	RM	Adult	Adult	0.072
Fold3.Rep05	Rep05	Fold3	RM	Adult	Adult	0.038
Fold3.Rep05	Rep05	Fold3	RM	Adult	Adult	0.328
Fold3.Rep05	Rep05	Fold3	RM	Adult	Adult	0.276
Fold3.Rep05	Rep05	Fold3	RM	Adult	Adult	0.212
Fold3.Rep05	Rep05	Fold3	RM	Pediatric	Pediatric	0.858
Fold3.Rep05	Rep05	Fold3	RM	Pediatric	Pediatric	0.888
Fold3.Rep05	Rep05	Fold3	RM	Pediatric	Pediatric	0.648
Fold3.Rep05	Rep05	Fold3	RM	Pediatric	Pediatric	0.618
Fold3.Rep05	Rep05	Fold3	RM	Adult	Adult	0.158
Fold3.Rep05	Rep05	Fold3	RM	Adult	Adult	0.166
Fold3.Rep05	Rep05	Fold3	RM	Adult	Adult	0.160
Fold3.Rep05	Rep05	Fold3	GBM	Adult	Adult	0.008
Fold3.Rep05	Rep05	Fold3	GBM	Adult	Adult	0.008
Fold3.Rep05	Rep05	Fold3	GBM	Adult	Adult	0.008
Fold3.Rep05	Rep05	Fold3	GBM	Adult	Adult	0.002
Fold3.Rep05	Rep05	Fold3	GBM	Adult	Adult	0.015
Fold3.Rep05	Rep05	Fold3	GBM	Adult	Adult	0.006
Fold3.Rep05	Rep05	Fold3	GBM	Adult	Adult	0.004
Fold3.Rep05	Rep05	Fold3	GBM	Pediatric	Pediatric	0.988
Fold3.Rep05	Rep05	Fold3	GBM	Pediatric	Pediatric	0.988
Fold3.Rep05	Rep05	Fold3	GBM	Pediatric	Pediatric	0.931
Fold3.Rep05	Rep05	Fold3	GBM	Pediatric	Pediatric	0.945
Fold3.Rep05	Rep05	Fold3	GBM	Adult	Adult	0.010
Fold3.Rep05	Rep05	Fold3	GBM	Adult	Adult	0.010
Fold3.Rep05	Rep05	Fold3	GBM	Adult	Adult	0.019

Fold3.Rep05	Rep05	Fold3	SVM	Adult	Adult	0.146
Fold3.Rep05	Rep05	Fold3	SVM	Adult	Adult	0.150
Fold3.Rep05	Rep05	Fold3	SVM	Adult	Adult	0.248
Fold3.Rep05	Rep05	Fold3	SVM	Adult	Adult	0.033
Fold3.Rep05	Rep05	Fold3	SVM	Adult	Adult	0.014
Fold3.Rep05	Rep05	Fold3	SVM	Adult	Pediatric	0.621
Fold3.Rep05	Rep05	Fold3	SVM	Adult	Adult	0.000
Fold3.Rep05	Rep05	Fold3	SVM	Pediatric	Pediatric	0.622
Fold3.Rep05	Rep05	Fold3	SVM	Pediatric	Adult	0.491
Fold3.Rep05	Rep05	Fold3	SVM	Pediatric	Pediatric	0.503
Fold3.Rep05	Rep05	Fold3	SVM	Pediatric	Adult	0.108
Fold3.Rep05	Rep05	Fold3	SVM	Adult	Adult	0.388
Fold3.Rep05	Rep05	Fold3	SVM	Adult	Adult	0.158
Fold3.Rep05	Rep05	Fold3	SVM	Adult	Adult	0.082
Fold4.Rep05	Rep05	Fold4	RM	Adult	Adult	0.018
Fold4.Rep05	Rep05	Fold4	RM	Adult	Adult	0.130
Fold4.Rep05	Rep05	Fold4	RM	Adult	Adult	0.136
Fold4.Rep05	Rep05	Fold4	RM	Adult	Adult	0.092
Fold4.Rep05	Rep05	Fold4	RM	Adult	Adult	0.148
Fold4.Rep05	Rep05	Fold4	RM	Adult	Adult	0.206
Fold4.Rep05	Rep05	Fold4	RM	Pediatric	Pediatric	0.836
Fold4.Rep05	Rep05	Fold4	RM	Pediatric	Pediatric	0.822
Fold4.Rep05	Rep05	Fold4	RM	Pediatric	Pediatric	0.852
Fold4.Rep05	Rep05	Fold4	RM	Pediatric	Pediatric	0.858
Fold4.Rep05	Rep05	Fold4	RM	Adult	Adult	0.066
Fold4.Rep05	Rep05	Fold4	RM	Adult	Adult	0.410
Fold4.Rep05	Rep05	Fold4	RM	Adult	Adult	0.030
Fold4.Rep05	Rep05	Fold4	RM	Adult	Adult	0.108
Fold4.Rep05	Rep05	Fold4	GBM	Adult	Adult	0.007
Fold4.Rep05	Rep05	Fold4	GBM	Adult	Adult	0.003
Fold4.Rep05	Rep05	Fold4	GBM	Adult	Adult	0.003
Fold4.Rep05	Rep05	Fold4	GBM	Adult	Adult	0.003
Fold4.Rep05	Rep05	Fold4	GBM	Adult	Adult	0.002
Fold4.Rep05	Rep05	Fold4	GBM	Adult	Adult	0.007
Fold4.Rep05	Rep05	Fold4	GBM	Pediatric	Pediatric	0.983
Fold4.Rep05	Rep05	Fold4	GBM	Pediatric	Pediatric	0.987
Fold4.Rep05	Rep05	Fold4	GBM	Pediatric	Pediatric	0.979
Fold4.Rep05	Rep05	Fold4	GBM	Pediatric	Pediatric	0.987
Fold4.Rep05	Rep05	Fold4	GBM	Adult	Adult	0.002
Fold4.Rep05	Rep05	Fold4	GBM	Adult	Adult	0.161
Fold4.Rep05	Rep05	Fold4	GBM	Adult	Adult	0.002
Fold4.Rep05	Rep05	Fold4	GBM	Adult	Adult	0.008
Fold4.Rep05	Rep05	Fold4	SVM	Adult	Adult	0.171
Fold4.Rep05	Rep05	Fold4	SVM	Adult	Adult	0.179
Fold4.Rep05	Rep05	Fold4	SVM	Adult	Adult	0.162
Fold4.Rep05	Rep05	Fold4	SVM	Adult	Adult	0.065
Fold4.Rep05	Rep05	Fold4	SVM	Adult	Adult	0.232
Fold4.Rep05	Rep05	Fold4	SVM	Adult	Pediatric	0.959
Fold4.Rep05	Rep05	Fold4	SVM	Pediatric	Pediatric	0.870
Fold4.Rep05	Rep05	Fold4	SVM	Pediatric	Adult	0.349
Fold4.Rep05	Rep05	Fold4	SVM	Pediatric	Pediatric	0.575
Fold4.Rep05	Rep05	Fold4	SVM	Pediatric	Pediatric	0.606
Fold4.Rep05	Rep05	Fold4	SVM	Adult	Adult	0.000
Fold4.Rep05	Rep05	Fold4	SVM	Adult	Adult	0.438
Fold4.Rep05	Rep05	Fold4	SVM	Adult	Adult	0.059
Fold4.Rep05	Rep05	Fold4	SVM	Adult	Adult	0.109
Fold5.Rep05	Rep05	Fold5	RM	Adult	Adult	0.036
Fold5.Rep05	Rep05	Fold5	RM	Adult	Adult	0.048
Fold5.Rep05	Rep05	Fold5	RM	Adult	Adult	0.120

Fold5.Rep05	Rep05	Fold5	RM	Adult	Adult	0.228
Fold5.Rep05	Rep05	Fold5	RM	Adult	Adult	0.150
Fold5.Rep05	Rep05	Fold5	RM	Pediatric	Pediatric	0.816
Fold5.Rep05	Rep05	Fold5	RM	Pediatric	Pediatric	0.810
Fold5.Rep05	Rep05	Fold5	RM	Pediatric	Pediatric	0.678
Fold5.Rep05	Rep05	Fold5	RM	Pediatric	Pediatric	0.734
Fold5.Rep05	Rep05	Fold5	RM	Adult	Adult	0.228
Fold5.Rep05	Rep05	Fold5	RM	Adult	Adult	0.190
Fold5.Rep05	Rep05	Fold5	RM	Adult	Adult	0.074
Fold5.Rep05	Rep05	Fold5	RM	Adult	Adult	0.102
Fold5.Rep05	Rep05	Fold5	GBM	Adult	Adult	0.004
Fold5.Rep05	Rep05	Fold5	GBM	Adult	Adult	0.004
Fold5.Rep05	Rep05	Fold5	GBM	Adult	Adult	0.003
Fold5.Rep05	Rep05	Fold5	GBM	Adult	Adult	0.006
Fold5.Rep05	Rep05	Fold5	GBM	Adult	Adult	0.003
Fold5.Rep05	Rep05	Fold5	GBM	Pediatric	Pediatric	0.987
Fold5.Rep05	Rep05	Fold5	GBM	Pediatric	Pediatric	0.970
Fold5.Rep05	Rep05	Fold5	GBM	Pediatric	Pediatric	0.939
Fold5.Rep05	Rep05	Fold5	GBM	Pediatric	Pediatric	0.978
Fold5.Rep05	Rep05	Fold5	GBM	Adult	Adult	0.014
Fold5.Rep05	Rep05	Fold5	GBM	Adult	Adult	0.007
Fold5.Rep05	Rep05	Fold5	GBM	Adult	Adult	0.004
Fold5.Rep05	Rep05	Fold5	GBM	Adult	Adult	0.002
Fold5.Rep05	Rep05	Fold5	SVM	Adult	Adult	0.091
Fold5.Rep05	Rep05	Fold5	SVM	Adult	Adult	0.089
Fold5.Rep05	Rep05	Fold5	SVM	Adult	Adult	0.000
Fold5.Rep05	Rep05	Fold5	SVM	Adult	Adult	0.000
Fold5.Rep05	Rep05	Fold5	SVM	Adult	Adult	0.000
Fold5.Rep05	Rep05	Fold5	SVM	Pediatric	Adult	0.296
Fold5.Rep05	Rep05	Fold5	SVM	Pediatric	Pediatric	1.000
Fold5.Rep05	Rep05	Fold5	SVM	Pediatric	Pediatric	0.948
Fold5.Rep05	Rep05	Fold5	SVM	Pediatric	Adult	0.386
Fold5.Rep05	Rep05	Fold5	SVM	Adult	Adult	0.000
Fold5.Rep05	Rep05	Fold5	SVM	Adult	Adult	0.058
Fold5.Rep05	Rep05	Fold5	SVM	Adult	Adult	0.006
Fold5.Rep05	Rep05	Fold5	SVM	Adult	Adult	0.019
Fold1.Rep06	Rep06	Fold1	RM	Adult	Adult	0.144
Fold1.Rep06	Rep06	Fold1	RM	Adult	Adult	0.056
Fold1.Rep06	Rep06	Fold1	RM	Adult	Adult	0.056
Fold1.Rep06	Rep06	Fold1	RM	Adult	Adult	0.046
Fold1.Rep06	Rep06	Fold1	RM	Adult	Adult	0.158
Fold1.Rep06	Rep06	Fold1	RM	Adult	Adult	0.184
Fold1.Rep06	Rep06	Fold1	RM	Adult	Adult	0.122
Fold1.Rep06	Rep06	Fold1	RM	Pediatric	Pediatric	0.820
Fold1.Rep06	Rep06	Fold1	RM	Pediatric	Pediatric	0.836
Fold1.Rep06	Rep06	Fold1	RM	Pediatric	Pediatric	0.680
Fold1.Rep06	Rep06	Fold1	RM	Pediatric	Pediatric	0.784
Fold1.Rep06	Rep06	Fold1	RM	Adult	Adult	0.154
Fold1.Rep06	Rep06	Fold1	RM	Adult	Adult	0.042
Fold1.Rep06	Rep06	Fold1	RM	Adult	Adult	0.088
Fold1.Rep06	Rep06	Fold1	GBM	Adult	Adult	0.006
Fold1.Rep06	Rep06	Fold1	GBM	Adult	Adult	0.007
Fold1.Rep06	Rep06	Fold1	GBM	Adult	Adult	0.002
Fold1.Rep06	Rep06	Fold1	GBM	Adult	Adult	0.002
Fold1.Rep06	Rep06	Fold1	GBM	Adult	Adult	0.002
Fold1.Rep06	Rep06	Fold1	GBM	Adult	Adult	0.006
Fold1.Rep06	Rep06	Fold1	GBM	Adult	Adult	0.003
Fold1.Rep06	Rep06	Fold1	GBM	Pediatric	Pediatric	0.981
Fold1.Rep06	Rep06	Fold1	GBM	Pediatric	Pediatric	0.969

Fold1.Rep06	Rep06	Fold1	GBM	Pediatric	Pediatric	0.899
Fold1.Rep06	Rep06	Fold1	GBM	Pediatric	Pediatric	0.981
Fold1.Rep06	Rep06	Fold1	GBM	Adult	Adult	0.004
Fold1.Rep06	Rep06	Fold1	GBM	Adult	Adult	0.002
Fold1.Rep06	Rep06	Fold1	GBM	Adult	Adult	0.007
Fold1.Rep06	Rep06	Fold1	SVM	Adult	Pediatric	1.000
Fold1.Rep06	Rep06	Fold1	SVM	Adult	Adult	0.166
Fold1.Rep06	Rep06	Fold1	SVM	Adult	Adult	0.000
Fold1.Rep06	Rep06	Fold1	SVM	Adult	Adult	0.182
Fold1.Rep06	Rep06	Fold1	SVM	Adult	Adult	0.276
Fold1.Rep06	Rep06	Fold1	SVM	Adult	Pediatric	1.000
Fold1.Rep06	Rep06	Fold1	SVM	Adult	Adult	0.000
Fold1.Rep06	Rep06	Fold1	SVM	Pediatric	Adult	0.466
Fold1.Rep06	Rep06	Fold1	SVM	Pediatric	Pediatric	1.000
Fold1.Rep06	Rep06	Fold1	SVM	Pediatric	Pediatric	0.607
Fold1.Rep06	Rep06	Fold1	SVM	Pediatric	Adult	0.255
Fold1.Rep06	Rep06	Fold1	SVM	Adult	Adult	0.063
Fold1.Rep06	Rep06	Fold1	SVM	Adult	Adult	0.039
Fold1.Rep06	Rep06	Fold1	SVM	Adult	Adult	0.094
Fold2.Rep06	Rep06	Fold2	RM	Adult	Adult	0.034
Fold2.Rep06	Rep06	Fold2	RM	Adult	Adult	0.058
Fold2.Rep06	Rep06	Fold2	RM	Adult	Adult	0.012
Fold2.Rep06	Rep06	Fold2	RM	Adult	Adult	0.038
Fold2.Rep06	Rep06	Fold2	RM	Adult	Adult	0.288
Fold2.Rep06	Rep06	Fold2	RM	Pediatric	Pediatric	0.832
Fold2.Rep06	Rep06	Fold2	RM	Pediatric	Pediatric	0.864
Fold2.Rep06	Rep06	Fold2	RM	Pediatric	Pediatric	0.840
Fold2.Rep06	Rep06	Fold2	RM	Pediatric	Pediatric	0.804
Fold2.Rep06	Rep06	Fold2	RM	Pediatric	Pediatric	0.782
Fold2.Rep06	Rep06	Fold2	RM	Adult	Adult	0.140
Fold2.Rep06	Rep06	Fold2	RM	Adult	Adult	0.472
Fold2.Rep06	Rep06	Fold2	RM	Adult	Adult	0.142
Fold2.Rep06	Rep06	Fold2	RM	Adult	Adult	0.178
Fold2.Rep06	Rep06	Fold2	GBM	Adult	Adult	0.010
Fold2.Rep06	Rep06	Fold2	GBM	Adult	Adult	0.010
Fold2.Rep06	Rep06	Fold2	GBM	Adult	Adult	0.002
Fold2.Rep06	Rep06	Fold2	GBM	Adult	Adult	0.010
Fold2.Rep06	Rep06	Fold2	GBM	Adult	Adult	0.071
Fold2.Rep06	Rep06	Fold2	GBM	Pediatric	Pediatric	0.937
Fold2.Rep06	Rep06	Fold2	GBM	Pediatric	Pediatric	0.976
Fold2.Rep06	Rep06	Fold2	GBM	Pediatric	Pediatric	0.976
Fold2.Rep06	Rep06	Fold2	GBM	Pediatric	Pediatric	0.970
Fold2.Rep06	Rep06	Fold2	GBM	Pediatric	Pediatric	0.966
Fold2.Rep06	Rep06	Fold2	GBM	Adult	Adult	0.026
Fold2.Rep06	Rep06	Fold2	GBM	Adult	Adult	0.385
Fold2.Rep06	Rep06	Fold2	GBM	Adult	Adult	0.026
Fold2.Rep06	Rep06	Fold2	GBM	Adult	Adult	0.061
Fold2.Rep06	Rep06	Fold2	SVM	Adult	Adult	0.124
Fold2.Rep06	Rep06	Fold2	SVM	Adult	Adult	0.271
Fold2.Rep06	Rep06	Fold2	SVM	Adult	Adult	0.088
Fold2.Rep06	Rep06	Fold2	SVM	Adult	Adult	0.205
Fold2.Rep06	Rep06	Fold2	SVM	Adult	Pediatric	0.623
Fold2.Rep06	Rep06	Fold2	SVM	Pediatric	Pediatric	0.583
Fold2.Rep06	Rep06	Fold2	SVM	Pediatric	Pediatric	0.646
Fold2.Rep06	Rep06	Fold2	SVM	Pediatric	Adult	0.399
Fold2.Rep06	Rep06	Fold2	SVM	Pediatric	Pediatric	0.719
Fold2.Rep06	Rep06	Fold2	SVM	Pediatric	Pediatric	0.558
Fold2.Rep06	Rep06	Fold2	SVM	Adult	Adult	0.340
Fold2.Rep06	Rep06	Fold2	SVM	Adult	Adult	0.399

Fold2.Rep06	Rep06	Fold2	SVM	Adult	Adult	0.075
Fold2.Rep06	Rep06	Fold2	SVM	Adult	Adult	0.072
Fold3.Rep06	Rep06	Fold3	RM	Adult	Adult	0.012
Fold3.Rep06	Rep06	Fold3	RM	Adult	Adult	0.148
Fold3.Rep06	Rep06	Fold3	RM	Adult	Adult	0.194
Fold3.Rep06	Rep06	Fold3	RM	Adult	Adult	0.266
Fold3.Rep06	Rep06	Fold3	RM	Pediatric	Pediatric	0.674
Fold3.Rep06	Rep06	Fold3	RM	Pediatric	Pediatric	0.858
Fold3.Rep06	Rep06	Fold3	RM	Pediatric	Pediatric	0.608
Fold3.Rep06	Rep06	Fold3	RM	Pediatric	Pediatric	0.742
Fold3.Rep06	Rep06	Fold3	RM	Adult	Adult	0.156
Fold3.Rep06	Rep06	Fold3	RM	Adult	Adult	0.074
Fold3.Rep06	Rep06	Fold3	RM	Adult	Adult	0.060
Fold3.Rep06	Rep06	Fold3	RM	Adult	Adult	0.190
Fold3.Rep06	Rep06	Fold3	RM	Adult	Adult	0.134
Fold3.Rep06	Rep06	Fold3	GBM	Adult	Adult	0.000
Fold3.Rep06	Rep06	Fold3	GBM	Adult	Adult	0.000
Fold3.Rep06	Rep06	Fold3	GBM	Adult	Adult	0.000
Fold3.Rep06	Rep06	Fold3	GBM	Adult	Adult	0.000
Fold3.Rep06	Rep06	Fold3	GBM	Pediatric	Pediatric	0.999
Fold3.Rep06	Rep06	Fold3	GBM	Pediatric	Pediatric	1.000
Fold3.Rep06	Rep06	Fold3	GBM	Pediatric	Pediatric	0.997
Fold3.Rep06	Rep06	Fold3	GBM	Pediatric	Pediatric	1.000
Fold3.Rep06	Rep06	Fold3	GBM	Adult	Adult	0.001
Fold3.Rep06	Rep06	Fold3	GBM	Adult	Adult	0.000
Fold3.Rep06	Rep06	Fold3	GBM	Adult	Adult	0.000
Fold3.Rep06	Rep06	Fold3	GBM	Adult	Adult	0.000
Fold3.Rep06	Rep06	Fold3	GBM	Adult	Adult	0.000
Fold3.Rep06	Rep06	Fold3	SVM	Adult	Adult	0.028
Fold3.Rep06	Rep06	Fold3	SVM	Adult	Pediatric	0.880
Fold3.Rep06	Rep06	Fold3	SVM	Adult	Adult	0.000
Fold3.Rep06	Rep06	Fold3	SVM	Adult	Pediatric	0.806
Fold3.Rep06	Rep06	Fold3	SVM	Pediatric	Pediatric	0.755
Fold3.Rep06	Rep06	Fold3	SVM	Pediatric	Pediatric	0.616
Fold3.Rep06	Rep06	Fold3	SVM	Pediatric	Adult	0.003
Fold3.Rep06	Rep06	Fold3	SVM	Pediatric	Pediatric	0.912
Fold3.Rep06	Rep06	Fold3	SVM	Adult	Adult	0.000
Fold3.Rep06	Rep06	Fold3	SVM	Adult	Adult	0.000
Fold3.Rep06	Rep06	Fold3	SVM	Adult	Adult	0.028
Fold3.Rep06	Rep06	Fold3	SVM	Adult	Adult	0.004
Fold3.Rep06	Rep06	Fold3	SVM	Adult	Adult	0.075
Fold4.Rep06	Rep06	Fold4	RM	Adult	Adult	0.032
Fold4.Rep06	Rep06	Fold4	RM	Adult	Adult	0.014
Fold4.Rep06	Rep06	Fold4	RM	Adult	Adult	0.084
Fold4.Rep06	Rep06	Fold4	RM	Adult	Adult	0.174
Fold4.Rep06	Rep06	Fold4	RM	Adult	Adult	0.282
Fold4.Rep06	Rep06	Fold4	RM	Adult	Adult	0.174
Fold4.Rep06	Rep06	Fold4	RM	Pediatric	Pediatric	0.802
Fold4.Rep06	Rep06	Fold4	RM	Pediatric	Pediatric	0.652
Fold4.Rep06	Rep06	Fold4	RM	Pediatric	Pediatric	0.708
Fold4.Rep06	Rep06	Fold4	RM	Pediatric	Pediatric	0.850
Fold4.Rep06	Rep06	Fold4	RM	Pediatric	Pediatric	0.766
Fold4.Rep06	Rep06	Fold4	RM	Adult	Adult	0.060
Fold4.Rep06	Rep06	Fold4	RM	Adult	Adult	0.032
Fold4.Rep06	Rep06	Fold4	RM	Adult	Adult	0.104
Fold4.Rep06	Rep06	Fold4	RM	Adult	Adult	0.234
Fold4.Rep06	Rep06	Fold4	GBM	Adult	Adult	0.007
Fold4.Rep06	Rep06	Fold4	GBM	Adult	Adult	0.007
Fold4.Rep06	Rep06	Fold4	GBM	Adult	Adult	0.012

Fold4.Rep06	Rep06	Fold4	GBM	Adult	Adult	0.005
Fold4.Rep06	Rep06	Fold4	GBM	Adult	Adult	0.012
Fold4.Rep06	Rep06	Fold4	GBM	Adult	Adult	0.005
Fold4.Rep06	Rep06	Fold4	GBM	Pediatric	Pediatric	0.981
Fold4.Rep06	Rep06	Fold4	GBM	Pediatric	Pediatric	0.920
Fold4.Rep06	Rep06	Fold4	GBM	Pediatric	Pediatric	0.911
Fold4.Rep06	Rep06	Fold4	GBM	Pediatric	Pediatric	0.983
Fold4.Rep06	Rep06	Fold4	GBM	Pediatric	Pediatric	0.930
Fold4.Rep06	Rep06	Fold4	GBM	Adult	Adult	0.003
Fold4.Rep06	Rep06	Fold4	GBM	Adult	Adult	0.002
Fold4.Rep06	Rep06	Fold4	GBM	Adult	Adult	0.007
Fold4.Rep06	Rep06	Fold4	GBM	Adult	Adult	0.035
Fold4.Rep06	Rep06	Fold4	SVM	Adult	Adult	0.031
Fold4.Rep06	Rep06	Fold4	SVM	Adult	Adult	0.046
Fold4.Rep06	Rep06	Fold4	SVM	Adult	Adult	0.156
Fold4.Rep06	Rep06	Fold4	SVM	Adult	Adult	0.496
Fold4.Rep06	Rep06	Fold4	SVM	Adult	Adult	0.227
Fold4.Rep06	Rep06	Fold4	SVM	Adult	Adult	0.000
Fold4.Rep06	Rep06	Fold4	SVM	Pediatric	Adult	0.393
Fold4.Rep06	Rep06	Fold4	SVM	Pediatric	Adult	0.455
Fold4.Rep06	Rep06	Fold4	SVM	Pediatric	Pediatric	0.677
Fold4.Rep06	Rep06	Fold4	SVM	Pediatric	Pediatric	0.814
Fold4.Rep06	Rep06	Fold4	SVM	Pediatric	Pediatric	0.956
Fold4.Rep06	Rep06	Fold4	SVM	Adult	Adult	0.242
Fold4.Rep06	Rep06	Fold4	SVM	Adult	Adult	0.001
Fold4.Rep06	Rep06	Fold4	SVM	Adult	Adult	0.207
Fold4.Rep06	Rep06	Fold4	SVM	Adult	Adult	0.067
Fold5.Rep06	Rep06	Fold5	RM	Adult	Adult	0.042
Fold5.Rep06	Rep06	Fold5	RM	Adult	Adult	0.180
Fold5.Rep06	Rep06	Fold5	RM	Adult	Adult	0.094
Fold5.Rep06	Rep06	Fold5	RM	Adult	Adult	0.030
Fold5.Rep06	Rep06	Fold5	RM	Adult	Adult	0.064
Fold5.Rep06	Rep06	Fold5	RM	Adult	Adult	0.024
Fold5.Rep06	Rep06	Fold5	RM	Adult	Adult	0.202
Fold5.Rep06	Rep06	Fold5	RM	Adult	Adult	0.238
Fold5.Rep06	Rep06	Fold5	RM	Pediatric	Pediatric	0.782
Fold5.Rep06	Rep06	Fold5	RM	Pediatric	Pediatric	0.656
Fold5.Rep06	Rep06	Fold5	RM	Pediatric	Pediatric	0.856
Fold5.Rep06	Rep06	Fold5	RM	Pediatric	Pediatric	0.720
Fold5.Rep06	Rep06	Fold5	RM	Adult	Adult	0.200
Fold5.Rep06	Rep06	Fold5	GBM	Adult	Adult	0.012
Fold5.Rep06	Rep06	Fold5	GBM	Adult	Adult	0.014
Fold5.Rep06	Rep06	Fold5	GBM	Adult	Adult	0.005
Fold5.Rep06	Rep06	Fold5	GBM	Adult	Adult	0.003
Fold5.Rep06	Rep06	Fold5	GBM	Adult	Adult	0.014
Fold5.Rep06	Rep06	Fold5	GBM	Adult	Adult	0.002
Fold5.Rep06	Rep06	Fold5	GBM	Adult	Adult	0.129
Fold5.Rep06	Rep06	Fold5	GBM	Adult	Adult	0.027
Fold5.Rep06	Rep06	Fold5	GBM	Pediatric	Pediatric	0.983
Fold5.Rep06	Rep06	Fold5	GBM	Pediatric	Pediatric	0.903
Fold5.Rep06	Rep06	Fold5	GBM	Pediatric	Pediatric	0.983
Fold5.Rep06	Rep06	Fold5	GBM	Pediatric	Pediatric	0.940
Fold5.Rep06	Rep06	Fold5	GBM	Adult	Adult	0.007
Fold5.Rep06	Rep06	Fold5	SVM	Adult	Adult	0.010
Fold5.Rep06	Rep06	Fold5	SVM	Adult	Pediatric	1.000
Fold5.Rep06	Rep06	Fold5	SVM	Adult	Adult	0.114
Fold5.Rep06	Rep06	Fold5	SVM	Adult	Adult	0.029
Fold5.Rep06	Rep06	Fold5	SVM	Adult	Adult	0.143
Fold5.Rep06	Rep06	Fold5	SVM	Adult	Adult	0.340

Fold5.Rep06	Rep06	Fold5	SVM	Adult	Pediatric	0.773
Fold5.Rep06	Rep06	Fold5	SVM	Adult	Adult	0.000
Fold5.Rep06	Rep06	Fold5	SVM	Pediatric	Pediatric	0.525
Fold5.Rep06	Rep06	Fold5	SVM	Pediatric	Pediatric	0.601
Fold5.Rep06	Rep06	Fold5	SVM	Pediatric	Pediatric	0.727
Fold5.Rep06	Rep06	Fold5	SVM	Pediatric	Adult	0.247
Fold5.Rep06	Rep06	Fold5	SVM	Adult	Adult	0.000
Fold1.Rep07	Rep07	Fold1	RM	Adult	Adult	0.022
Fold1.Rep07	Rep07	Fold1	RM	Adult	Adult	0.056
Fold1.Rep07	Rep07	Fold1	RM	Adult	Adult	0.064
Fold1.Rep07	Rep07	Fold1	RM	Adult	Adult	0.018
Fold1.Rep07	Rep07	Fold1	RM	Adult	Adult	0.176
Fold1.Rep07	Rep07	Fold1	RM	Adult	Adult	0.154
Fold1.Rep07	Rep07	Fold1	RM	Pediatric	Pediatric	0.848
Fold1.Rep07	Rep07	Fold1	RM	Pediatric	Pediatric	0.828
Fold1.Rep07	Rep07	Fold1	RM	Pediatric	Pediatric	0.684
Fold1.Rep07	Rep07	Fold1	RM	Pediatric	Pediatric	0.752
Fold1.Rep07	Rep07	Fold1	RM	Adult	Adult	0.064
Fold1.Rep07	Rep07	Fold1	RM	Adult	Adult	0.028
Fold1.Rep07	Rep07	Fold1	RM	Adult	Adult	0.110
Fold1.Rep07	Rep07	Fold1	RM	Adult	Adult	0.124
Fold1.Rep07	Rep07	Fold1	GBM	Adult	Adult	0.008
Fold1.Rep07	Rep07	Fold1	GBM	Adult	Adult	0.008
Fold1.Rep07	Rep07	Fold1	GBM	Adult	Adult	0.008
Fold1.Rep07	Rep07	Fold1	GBM	Adult	Adult	0.003
Fold1.Rep07	Rep07	Fold1	GBM	Adult	Adult	0.006
Fold1.Rep07	Rep07	Fold1	GBM	Adult	Adult	0.010
Fold1.Rep07	Rep07	Fold1	GBM	Pediatric	Pediatric	0.969
Fold1.Rep07	Rep07	Fold1	GBM	Pediatric	Pediatric	0.960
Fold1.Rep07	Rep07	Fold1	GBM	Pediatric	Pediatric	0.981
Fold1.Rep07	Rep07	Fold1	GBM	Pediatric	Pediatric	0.944
Fold1.Rep07	Rep07	Fold1	GBM	Adult	Adult	0.002
Fold1.Rep07	Rep07	Fold1	GBM	Adult	Adult	0.003
Fold1.Rep07	Rep07	Fold1	GBM	Adult	Adult	0.010
Fold1.Rep07	Rep07	Fold1	GBM	Adult	Adult	0.006
Fold1.Rep07	Rep07	Fold1	SVM	Adult	Adult	0.035
Fold1.Rep07	Rep07	Fold1	SVM	Adult	Adult	0.025
Fold1.Rep07	Rep07	Fold1	SVM	Adult	Adult	0.085
Fold1.Rep07	Rep07	Fold1	SVM	Adult	Adult	0.071
Fold1.Rep07	Rep07	Fold1	SVM	Adult	Pediatric	0.894
Fold1.Rep07	Rep07	Fold1	SVM	Adult	Adult	0.000
Fold1.Rep07	Rep07	Fold1	SVM	Pediatric	Pediatric	0.785
Fold1.Rep07	Rep07	Fold1	SVM	Pediatric	Pediatric	1.000
Fold1.Rep07	Rep07	Fold1	SVM	Pediatric	Pediatric	0.791
Fold1.Rep07	Rep07	Fold1	SVM	Pediatric	Pediatric	0.840
Fold1.Rep07	Rep07	Fold1	SVM	Adult	Adult	0.000
Fold1.Rep07	Rep07	Fold1	SVM	Adult	Adult	0.000
Fold1.Rep07	Rep07	Fold1	SVM	Adult	Adult	0.012
Fold1.Rep07	Rep07	Fold1	SVM	Adult	Adult	0.001
Fold2.Rep07	Rep07	Fold2	RM	Adult	Adult	0.034
Fold2.Rep07	Rep07	Fold2	RM	Adult	Adult	0.068
Fold2.Rep07	Rep07	Fold2	RM	Adult	Adult	0.028
Fold2.Rep07	Rep07	Fold2	RM	Adult	Adult	0.032
Fold2.Rep07	Rep07	Fold2	RM	Adult	Adult	0.106
Fold2.Rep07	Rep07	Fold2	RM	Pediatric	Pediatric	0.570
Fold2.Rep07	Rep07	Fold2	RM	Pediatric	Pediatric	0.692
Fold2.Rep07	Rep07	Fold2	RM	Pediatric	Pediatric	0.764
Fold2.Rep07	Rep07	Fold2	RM	Pediatric	Pediatric	0.538
Fold2.Rep07	Rep07	Fold2	RM	Adult	Adult	0.066

Fold2.Rep07	Rep07	Fold2	RM	Adult	Adult	0.084
Fold2.Rep07	Rep07	Fold2	RM	Adult	Adult	0.390
Fold2.Rep07	Rep07	Fold2	RM	Adult	Adult	0.056
Fold2.Rep07	Rep07	Fold2	GBM	Adult	Adult	0.007
Fold2.Rep07	Rep07	Fold2	GBM	Adult	Adult	0.004
Fold2.Rep07	Rep07	Fold2	GBM	Adult	Adult	0.002
Fold2.Rep07	Rep07	Fold2	GBM	Adult	Adult	0.002
Fold2.Rep07	Rep07	Fold2	GBM	Adult	Adult	0.003
Fold2.Rep07	Rep07	Fold2	GBM	Pediatric	Pediatric	0.906
Fold2.Rep07	Rep07	Fold2	GBM	Pediatric	Pediatric	0.949
Fold2.Rep07	Rep07	Fold2	GBM	Pediatric	Pediatric	0.962
Fold2.Rep07	Rep07	Fold2	GBM	Pediatric	Pediatric	0.906
Fold2.Rep07	Rep07	Fold2	GBM	Adult	Adult	0.002
Fold2.Rep07	Rep07	Fold2	GBM	Adult	Adult	0.007
Fold2.Rep07	Rep07	Fold2	GBM	Adult	Adult	0.281
Fold2.Rep07	Rep07	Fold2	GBM	Adult	Adult	0.002
Fold2.Rep07	Rep07	Fold2	SVM	Adult	Adult	0.199
Fold2.Rep07	Rep07	Fold2	SVM	Adult	Adult	0.039
Fold2.Rep07	Rep07	Fold2	SVM	Adult	Adult	0.034
Fold2.Rep07	Rep07	Fold2	SVM	Adult	Adult	0.029
Fold2.Rep07	Rep07	Fold2	SVM	Adult	Adult	0.000
Fold2.Rep07	Rep07	Fold2	SVM	Pediatric	Pediatric	0.537
Fold2.Rep07	Rep07	Fold2	SVM	Pediatric	Pediatric	0.573
Fold2.Rep07	Rep07	Fold2	SVM	Pediatric	Pediatric	0.616
Fold2.Rep07	Rep07	Fold2	SVM	Pediatric	Adult	0.471
Fold2.Rep07	Rep07	Fold2	SVM	Adult	Adult	0.214
Fold2.Rep07	Rep07	Fold2	SVM	Adult	Adult	0.081
Fold2.Rep07	Rep07	Fold2	SVM	Adult	Adult	0.417
Fold2.Rep07	Rep07	Fold2	SVM	Adult	Adult	0.022
Fold3.Rep07	Rep07	Fold3	RM	Adult	Adult	0.022
Fold3.Rep07	Rep07	Fold3	RM	Adult	Adult	0.138
Fold3.Rep07	Rep07	Fold3	RM	Adult	Adult	0.050
Fold3.Rep07	Rep07	Fold3	RM	Adult	Adult	0.378
Fold3.Rep07	Rep07	Fold3	RM	Adult	Adult	0.134
Fold3.Rep07	Rep07	Fold3	RM	Adult	Adult	0.280
Fold3.Rep07	Rep07	Fold3	RM	Adult	Adult	0.236
Fold3.Rep07	Rep07	Fold3	RM	Pediatric	Pediatric	0.850
Fold3.Rep07	Rep07	Fold3	RM	Pediatric	Pediatric	0.840
Fold3.Rep07	Rep07	Fold3	RM	Pediatric	Pediatric	0.872
Fold3.Rep07	Rep07	Fold3	RM	Pediatric	Pediatric	0.788
Fold3.Rep07	Rep07	Fold3	RM	Adult	Adult	0.190
Fold3.Rep07	Rep07	Fold3	RM	Adult	Adult	0.124
Fold3.Rep07	Rep07	Fold3	RM	Adult	Adult	0.176
Fold3.Rep07	Rep07	Fold3	GBM	Adult	Adult	0.005
Fold3.Rep07	Rep07	Fold3	GBM	Adult	Adult	0.005
Fold3.Rep07	Rep07	Fold3	GBM	Adult	Adult	0.005
Fold3.Rep07	Rep07	Fold3	GBM	Adult	Adult	0.015
Fold3.Rep07	Rep07	Fold3	GBM	Adult	Adult	0.002
Fold3.Rep07	Rep07	Fold3	GBM	Adult	Adult	0.013
Fold3.Rep07	Rep07	Fold3	GBM	Adult	Adult	0.007
Fold3.Rep07	Rep07	Fold3	GBM	Pediatric	Pediatric	0.983
Fold3.Rep07	Rep07	Fold3	GBM	Pediatric	Pediatric	0.978
Fold3.Rep07	Rep07	Fold3	GBM	Pediatric	Pediatric	0.980
Fold3.Rep07	Rep07	Fold3	GBM	Pediatric	Pediatric	0.976
Fold3.Rep07	Rep07	Fold3	GBM	Adult	Adult	0.004
Fold3.Rep07	Rep07	Fold3	GBM	Adult	Adult	0.002
Fold3.Rep07	Rep07	Fold3	GBM	Adult	Adult	0.023
Fold3.Rep07	Rep07	Fold3	SVM	Adult	Adult	0.001
Fold3.Rep07	Rep07	Fold3	SVM	Adult	Adult	0.079

Fold3.Rep07	Rep07	Fold3	SVM	Adult	Adult	0.073
Fold3.Rep07	Rep07	Fold3	SVM	Adult	Adult	0.236
Fold3.Rep07	Rep07	Fold3	SVM	Adult	Adult	0.051
Fold3.Rep07	Rep07	Fold3	SVM	Adult	Adult	0.001
Fold3.Rep07	Rep07	Fold3	SVM	Adult	Pediatric	0.722
Fold3.Rep07	Rep07	Fold3	SVM	Pediatric	Adult	0.312
Fold3.Rep07	Rep07	Fold3	SVM	Pediatric	Adult	0.285
Fold3.Rep07	Rep07	Fold3	SVM	Pediatric	Pediatric	0.773
Fold3.Rep07	Rep07	Fold3	SVM	Pediatric	Adult	0.455
Fold3.Rep07	Rep07	Fold3	SVM	Adult	Adult	0.000
Fold3.Rep07	Rep07	Fold3	SVM	Adult	Adult	0.019
Fold3.Rep07	Rep07	Fold3	SVM	Adult	Adult	0.004
Fold4.Rep07	Rep07	Fold4	RM	Adult	Adult	0.158
Fold4.Rep07	Rep07	Fold4	RM	Adult	Adult	0.052
Fold4.Rep07	Rep07	Fold4	RM	Adult	Adult	0.016
Fold4.Rep07	Rep07	Fold4	RM	Adult	Adult	0.100
Fold4.Rep07	Rep07	Fold4	RM	Adult	Adult	0.222
Fold4.Rep07	Rep07	Fold4	RM	Adult	Adult	0.208
Fold4.Rep07	Rep07	Fold4	RM	Adult	Adult	0.192
Fold4.Rep07	Rep07	Fold4	RM	Pediatric	Pediatric	0.930
Fold4.Rep07	Rep07	Fold4	RM	Pediatric	Pediatric	0.844
Fold4.Rep07	Rep07	Fold4	RM	Pediatric	Pediatric	0.642
Fold4.Rep07	Rep07	Fold4	RM	Pediatric	Pediatric	0.840
Fold4.Rep07	Rep07	Fold4	RM	Pediatric	Pediatric	0.770
Fold4.Rep07	Rep07	Fold4	RM	Adult	Adult	0.100
Fold4.Rep07	Rep07	Fold4	RM	Adult	Adult	0.134
Fold4.Rep07	Rep07	Fold4	GBM	Adult	Adult	0.009
Fold4.Rep07	Rep07	Fold4	GBM	Adult	Adult	0.004
Fold4.Rep07	Rep07	Fold4	GBM	Adult	Adult	0.004
Fold4.Rep07	Rep07	Fold4	GBM	Adult	Adult	0.004
Fold4.Rep07	Rep07	Fold4	GBM	Adult	Adult	0.224
Fold4.Rep07	Rep07	Fold4	GBM	Adult	Adult	0.010
Fold4.Rep07	Rep07	Fold4	GBM	Adult	Adult	0.013
Fold4.Rep07	Rep07	Fold4	GBM	Pediatric	Pediatric	0.984
Fold4.Rep07	Rep07	Fold4	GBM	Pediatric	Pediatric	0.978
Fold4.Rep07	Rep07	Fold4	GBM	Pediatric	Pediatric	0.824
Fold4.Rep07	Rep07	Fold4	GBM	Pediatric	Pediatric	0.951
Fold4.Rep07	Rep07	Fold4	GBM	Pediatric	Pediatric	0.974
Fold4.Rep07	Rep07	Fold4	GBM	Adult	Adult	0.007
Fold4.Rep07	Rep07	Fold4	GBM	Adult	Adult	0.011
Fold4.Rep07	Rep07	Fold4	SVM	Adult	Adult	0.154
Fold4.Rep07	Rep07	Fold4	SVM	Adult	Adult	0.047
Fold4.Rep07	Rep07	Fold4	SVM	Adult	Adult	0.109
Fold4.Rep07	Rep07	Fold4	SVM	Adult	Adult	0.472
Fold4.Rep07	Rep07	Fold4	SVM	Adult	Pediatric	0.673
Fold4.Rep07	Rep07	Fold4	SVM	Adult	Pediatric	0.651
Fold4.Rep07	Rep07	Fold4	SVM	Adult	Pediatric	1.000
Fold4.Rep07	Rep07	Fold4	SVM	Pediatric	Adult	0.439
Fold4.Rep07	Rep07	Fold4	SVM	Pediatric	Pediatric	0.634
Fold4.Rep07	Rep07	Fold4	SVM	Pediatric	Adult	0.325
Fold4.Rep07	Rep07	Fold4	SVM	Pediatric	Pediatric	0.860
Fold4.Rep07	Rep07	Fold4	SVM	Pediatric	Pediatric	0.751
Fold4.Rep07	Rep07	Fold4	SVM	Adult	Adult	0.386
Fold4.Rep07	Rep07	Fold4	SVM	Adult	Adult	0.146
Fold5.Rep07	Rep07	Fold5	RM	Adult	Adult	0.146
Fold5.Rep07	Rep07	Fold5	RM	Adult	Adult	0.036
Fold5.Rep07	Rep07	Fold5	RM	Adult	Adult	0.036
Fold5.Rep07	Rep07	Fold5	RM	Adult	Adult	0.042
Fold5.Rep07	Rep07	Fold5	RM	Adult	Adult	0.272

Fold5.Rep07	Rep07	Fold5	RM	Pediatric	Pediatric	0.838
Fold5.Rep07	Rep07	Fold5	RM	Pediatric	Pediatric	0.682
Fold5.Rep07	Rep07	Fold5	RM	Pediatric	Pediatric	0.804
Fold5.Rep07	Rep07	Fold5	RM	Pediatric	Pediatric	0.842
Fold5.Rep07	Rep07	Fold5	RM	Pediatric	Pediatric	0.850
Fold5.Rep07	Rep07	Fold5	RM	Adult	Adult	0.214
Fold5.Rep07	Rep07	Fold5	RM	Adult	Adult	0.180
Fold5.Rep07	Rep07	Fold5	RM	Adult	Adult	0.270
Fold5.Rep07	Rep07	Fold5	RM	Adult	Adult	0.276
Fold5.Rep07	Rep07	Fold5	GBM	Adult	Adult	0.024
Fold5.Rep07	Rep07	Fold5	GBM	Adult	Adult	0.024
Fold5.Rep07	Rep07	Fold5	GBM	Adult	Adult	0.024
Fold5.Rep07	Rep07	Fold5	GBM	Adult	Adult	0.024
Fold5.Rep07	Rep07	Fold5	GBM	Adult	Adult	0.060
Fold5.Rep07	Rep07	Fold5	GBM	Pediatric	Pediatric	0.970
Fold5.Rep07	Rep07	Fold5	GBM	Pediatric	Pediatric	0.955
Fold5.Rep07	Rep07	Fold5	GBM	Pediatric	Pediatric	0.941
Fold5.Rep07	Rep07	Fold5	GBM	Pediatric	Pediatric	0.974
Fold5.Rep07	Rep07	Fold5	GBM	Pediatric	Pediatric	0.979
Fold5.Rep07	Rep07	Fold5	GBM	Adult	Adult	0.033
Fold5.Rep07	Rep07	Fold5	GBM	Adult	Adult	0.013
Fold5.Rep07	Rep07	Fold5	GBM	Adult	Adult	0.006
Fold5.Rep07	Rep07	Fold5	GBM	Adult	Adult	0.057
Fold5.Rep07	Rep07	Fold5	SVM	Adult	Pediatric	1.000
Fold5.Rep07	Rep07	Fold5	SVM	Adult	Adult	0.045
Fold5.Rep07	Rep07	Fold5	SVM	Adult	Adult	0.064
Fold5.Rep07	Rep07	Fold5	SVM	Adult	Adult	0.100
Fold5.Rep07	Rep07	Fold5	SVM	Adult	Pediatric	0.505
Fold5.Rep07	Rep07	Fold5	SVM	Pediatric	Pediatric	0.781
Fold5.Rep07	Rep07	Fold5	SVM	Pediatric	Adult	0.448
Fold5.Rep07	Rep07	Fold5	SVM	Pediatric	Pediatric	0.749
Fold5.Rep07	Rep07	Fold5	SVM	Pediatric	Pediatric	0.507
Fold5.Rep07	Rep07	Fold5	SVM	Pediatric	Pediatric	0.712
Fold5.Rep07	Rep07	Fold5	SVM	Adult	Adult	0.000
Fold5.Rep07	Rep07	Fold5	SVM	Adult	Adult	0.000
Fold5.Rep07	Rep07	Fold5	SVM	Adult	Adult	0.006
Fold5.Rep07	Rep07	Fold5	SVM	Adult	Adult	0.046
Fold1.Rep08	Rep08	Fold1	RM	Adult	Adult	0.020
Fold1.Rep08	Rep08	Fold1	RM	Adult	Adult	0.098
Fold1.Rep08	Rep08	Fold1	RM	Adult	Adult	0.068
Fold1.Rep08	Rep08	Fold1	RM	Adult	Adult	0.260
Fold1.Rep08	Rep08	Fold1	RM	Adult	Adult	0.158
Fold1.Rep08	Rep08	Fold1	RM	Pediatric	Pediatric	0.724
Fold1.Rep08	Rep08	Fold1	RM	Pediatric	Pediatric	0.866
Fold1.Rep08	Rep08	Fold1	RM	Pediatric	Pediatric	0.616
Fold1.Rep08	Rep08	Fold1	RM	Pediatric	Pediatric	0.702
Fold1.Rep08	Rep08	Fold1	RM	Adult	Adult	0.406
Fold1.Rep08	Rep08	Fold1	RM	Adult	Adult	0.068
Fold1.Rep08	Rep08	Fold1	RM	Adult	Adult	0.088
Fold1.Rep08	Rep08	Fold1	RM	Adult	Adult	0.072
Fold1.Rep08	Rep08	Fold1	RM	Adult	Adult	0.106
Fold1.Rep08	Rep08	Fold1	GBM	Adult	Adult	0.004
Fold1.Rep08	Rep08	Fold1	GBM	Adult	Adult	0.004
Fold1.Rep08	Rep08	Fold1	GBM	Adult	Adult	0.005
Fold1.Rep08	Rep08	Fold1	GBM	Adult	Adult	0.013
Fold1.Rep08	Rep08	Fold1	GBM	Adult	Adult	0.006
Fold1.Rep08	Rep08	Fold1	GBM	Pediatric	Pediatric	0.990
Fold1.Rep08	Rep08	Fold1	GBM	Pediatric	Pediatric	0.990
Fold1.Rep08	Rep08	Fold1	GBM	Pediatric	Pediatric	0.973

Fold1.Rep08	Rep08	Fold1	GBM	Pediatric	Pediatric	0.973
Fold1.Rep08	Rep08	Fold1	GBM	Adult	Adult	0.358
Fold1.Rep08	Rep08	Fold1	GBM	Adult	Adult	0.002
Fold1.Rep08	Rep08	Fold1	GBM	Adult	Adult	0.008
Fold1.Rep08	Rep08	Fold1	GBM	Adult	Adult	0.002
Fold1.Rep08	Rep08	Fold1	GBM	Adult	Adult	0.004
Fold1.Rep08	Rep08	Fold1	SVM	Adult	Adult	0.002
Fold1.Rep08	Rep08	Fold1	SVM	Adult	Adult	0.121
Fold1.Rep08	Rep08	Fold1	SVM	Adult	Adult	0.426
Fold1.Rep08	Rep08	Fold1	SVM	Adult	Adult	0.175
Fold1.Rep08	Rep08	Fold1	SVM	Adult	Adult	0.000
Fold1.Rep08	Rep08	Fold1	SVM	Pediatric	Pediatric	0.737
Fold1.Rep08	Rep08	Fold1	SVM	Pediatric	Pediatric	0.733
Fold1.Rep08	Rep08	Fold1	SVM	Pediatric	Adult	0.050
Fold1.Rep08	Rep08	Fold1	SVM	Pediatric	Pediatric	0.870
Fold1.Rep08	Rep08	Fold1	SVM	Adult	Adult	0.253
Fold1.Rep08	Rep08	Fold1	SVM	Adult	Adult	0.000
Fold1.Rep08	Rep08	Fold1	SVM	Adult	Adult	0.020
Fold1.Rep08	Rep08	Fold1	SVM	Adult	Adult	0.005
Fold1.Rep08	Rep08	Fold1	SVM	Adult	Adult	0.006
Fold2.Rep08	Rep08	Fold2	RM	Adult	Adult	0.026
Fold2.Rep08	Rep08	Fold2	RM	Adult	Adult	0.032
Fold2.Rep08	Rep08	Fold2	RM	Adult	Adult	0.026
Fold2.Rep08	Rep08	Fold2	RM	Adult	Adult	0.364
Fold2.Rep08	Rep08	Fold2	RM	Adult	Adult	0.206
Fold2.Rep08	Rep08	Fold2	RM	Adult	Adult	0.170
Fold2.Rep08	Rep08	Fold2	RM	Pediatric	Pediatric	0.792
Fold2.Rep08	Rep08	Fold2	RM	Pediatric	Pediatric	0.732
Fold2.Rep08	Rep08	Fold2	RM	Pediatric	Pediatric	0.836
Fold2.Rep08	Rep08	Fold2	RM	Pediatric	Pediatric	0.690
Fold2.Rep08	Rep08	Fold2	RM	Pediatric	Pediatric	0.896
Fold2.Rep08	Rep08	Fold2	RM	Adult	Adult	0.208
Fold2.Rep08	Rep08	Fold2	RM	Adult	Adult	0.068
Fold2.Rep08	Rep08	Fold2	RM	Adult	Adult	0.330
Fold2.Rep08	Rep08	Fold2	RM	Adult	Adult	0.114
Fold2.Rep08	Rep08	Fold2	GBM	Adult	Adult	0.004
Fold2.Rep08	Rep08	Fold2	GBM	Adult	Adult	0.005
Fold2.Rep08	Rep08	Fold2	GBM	Adult	Adult	0.004
Fold2.Rep08	Rep08	Fold2	GBM	Adult	Adult	0.036
Fold2.Rep08	Rep08	Fold2	GBM	Adult	Adult	0.002
Fold2.Rep08	Rep08	Fold2	GBM	Adult	Adult	0.006
Fold2.Rep08	Rep08	Fold2	GBM	Pediatric	Pediatric	0.978
Fold2.Rep08	Rep08	Fold2	GBM	Pediatric	Pediatric	0.966
Fold2.Rep08	Rep08	Fold2	GBM	Pediatric	Pediatric	0.984
Fold2.Rep08	Rep08	Fold2	GBM	Pediatric	Pediatric	0.967
Fold2.Rep08	Rep08	Fold2	GBM	Pediatric	Pediatric	0.987
Fold2.Rep08	Rep08	Fold2	GBM	Adult	Adult	0.035
Fold2.Rep08	Rep08	Fold2	GBM	Adult	Adult	0.002
Fold2.Rep08	Rep08	Fold2	GBM	Adult	Adult	0.057
Fold2.Rep08	Rep08	Fold2	GBM	Adult	Adult	0.002
Fold2.Rep08	Rep08	Fold2	SVM	Adult	Adult	0.079
Fold2.Rep08	Rep08	Fold2	SVM	Adult	Adult	0.162
Fold2.Rep08	Rep08	Fold2	SVM	Adult	Adult	0.121
Fold2.Rep08	Rep08	Fold2	SVM	Adult	Adult	0.000
Fold2.Rep08	Rep08	Fold2	SVM	Adult	Adult	0.123
Fold2.Rep08	Rep08	Fold2	SVM	Adult	Adult	0.000
Fold2.Rep08	Rep08	Fold2	SVM	Pediatric	Pediatric	1.000
Fold2.Rep08	Rep08	Fold2	SVM	Pediatric	Adult	0.430
Fold2.Rep08	Rep08	Fold2	SVM	Pediatric	Pediatric	0.685

Fold2.Rep08	Rep08	Fold2	SVM	Pediatric	Pediatric	0.811
Fold2.Rep08	Rep08	Fold2	SVM	Pediatric	Pediatric	0.734
Fold2.Rep08	Rep08	Fold2	SVM	Adult	Adult	0.000
Fold2.Rep08	Rep08	Fold2	SVM	Adult	Adult	0.000
Fold2.Rep08	Rep08	Fold2	SVM	Adult	Adult	0.035
Fold2.Rep08	Rep08	Fold2	SVM	Adult	Adult	0.091
Fold3.Rep08	Rep08	Fold3	RM	Adult	Adult	0.034
Fold3.Rep08	Rep08	Fold3	RM	Adult	Adult	0.166
Fold3.Rep08	Rep08	Fold3	RM	Adult	Adult	0.058
Fold3.Rep08	Rep08	Fold3	RM	Adult	Adult	0.100
Fold3.Rep08	Rep08	Fold3	RM	Adult	Adult	0.004
Fold3.Rep08	Rep08	Fold3	RM	Adult	Adult	0.050
Fold3.Rep08	Rep08	Fold3	RM	Adult	Adult	0.148
Fold3.Rep08	Rep08	Fold3	RM	Adult	Adult	0.240
Fold3.Rep08	Rep08	Fold3	RM	Pediatric	Pediatric	0.842
Fold3.Rep08	Rep08	Fold3	RM	Pediatric	Pediatric	0.672
Fold3.Rep08	Rep08	Fold3	RM	Pediatric	Pediatric	0.696
Fold3.Rep08	Rep08	Fold3	RM	Pediatric	Pediatric	0.798
Fold3.Rep08	Rep08	Fold3	RM	Adult	Adult	0.180
Fold3.Rep08	Rep08	Fold3	GBM	Adult	Adult	0.005
Fold3.Rep08	Rep08	Fold3	GBM	Adult	Adult	0.005
Fold3.Rep08	Rep08	Fold3	GBM	Adult	Adult	0.002
Fold3.Rep08	Rep08	Fold3	GBM	Adult	Adult	0.004
Fold3.Rep08	Rep08	Fold3	GBM	Adult	Adult	0.002
Fold3.Rep08	Rep08	Fold3	GBM	Adult	Adult	0.005
Fold3.Rep08	Rep08	Fold3	GBM	Adult	Adult	0.008
Fold3.Rep08	Rep08	Fold3	GBM	Adult	Adult	0.091
Fold3.Rep08	Rep08	Fold3	GBM	Pediatric	Pediatric	0.988
Fold3.Rep08	Rep08	Fold3	GBM	Pediatric	Pediatric	0.967
Fold3.Rep08	Rep08	Fold3	GBM	Pediatric	Pediatric	0.976
Fold3.Rep08	Rep08	Fold3	GBM	Pediatric	Pediatric	0.986
Fold3.Rep08	Rep08	Fold3	GBM	Adult	Adult	0.003
Fold3.Rep08	Rep08	Fold3	SVM	Adult	Adult	0.116
Fold3.Rep08	Rep08	Fold3	SVM	Adult	Pediatric	1.000
Fold3.Rep08	Rep08	Fold3	SVM	Adult	Adult	0.074
Fold3.Rep08	Rep08	Fold3	SVM	Adult	Adult	0.135
Fold3.Rep08	Rep08	Fold3	SVM	Adult	Adult	0.174
Fold3.Rep08	Rep08	Fold3	SVM	Adult	Adult	0.207
Fold3.Rep08	Rep08	Fold3	SVM	Adult	Pediatric	0.713
Fold3.Rep08	Rep08	Fold3	SVM	Adult	Pediatric	0.761
Fold3.Rep08	Rep08	Fold3	SVM	Pediatric	Pediatric	0.640
Fold3.Rep08	Rep08	Fold3	SVM	Pediatric	Adult	0.178
Fold3.Rep08	Rep08	Fold3	SVM	Pediatric	Pediatric	0.603
Fold3.Rep08	Rep08	Fold3	SVM	Pediatric	Pediatric	0.926
Fold3.Rep08	Rep08	Fold3	SVM	Adult	Adult	0.014
Fold4.Rep08	Rep08	Fold4	RM	Adult	Adult	0.024
Fold4.Rep08	Rep08	Fold4	RM	Adult	Adult	0.136
Fold4.Rep08	Rep08	Fold4	RM	Adult	Adult	0.016
Fold4.Rep08	Rep08	Fold4	RM	Adult	Adult	0.172
Fold4.Rep08	Rep08	Fold4	RM	Adult	Adult	0.164
Fold4.Rep08	Rep08	Fold4	RM	Pediatric	Pediatric	0.846
Fold4.Rep08	Rep08	Fold4	RM	Pediatric	Pediatric	0.816
Fold4.Rep08	Rep08	Fold4	RM	Pediatric	Pediatric	0.808
Fold4.Rep08	Rep08	Fold4	RM	Pediatric	Pediatric	0.714
Fold4.Rep08	Rep08	Fold4	RM	Adult	Adult	0.122
Fold4.Rep08	Rep08	Fold4	RM	Adult	Adult	0.218
Fold4.Rep08	Rep08	Fold4	RM	Adult	Adult	0.064
Fold4.Rep08	Rep08	Fold4	RM	Adult	Adult	0.164
Fold4.Rep08	Rep08	Fold4	GBM	Adult	Adult	0.005

Fold4.Rep08	Rep08	Fold4	GBM	Adult	Adult	0.007
Fold4.Rep08	Rep08	Fold4	GBM	Adult	Adult	0.002
Fold4.Rep08	Rep08	Fold4	GBM	Adult	Adult	0.003
Fold4.Rep08	Rep08	Fold4	GBM	Adult	Adult	0.005
Fold4.Rep08	Rep08	Fold4	GBM	Pediatric	Pediatric	0.986
Fold4.Rep08	Rep08	Fold4	GBM	Pediatric	Pediatric	0.974
Fold4.Rep08	Rep08	Fold4	GBM	Pediatric	Pediatric	0.981
Fold4.Rep08	Rep08	Fold4	GBM	Pediatric	Pediatric	0.980
Fold4.Rep08	Rep08	Fold4	GBM	Adult	Adult	0.007
Fold4.Rep08	Rep08	Fold4	GBM	Adult	Adult	0.009
Fold4.Rep08	Rep08	Fold4	GBM	Adult	Adult	0.005
Fold4.Rep08	Rep08	Fold4	GBM	Adult	Adult	0.019
Fold4.Rep08	Rep08	Fold4	SVM	Adult	Adult	0.116
Fold4.Rep08	Rep08	Fold4	SVM	Adult	Adult	0.102
Fold4.Rep08	Rep08	Fold4	SVM	Adult	Adult	0.041
Fold4.Rep08	Rep08	Fold4	SVM	Adult	Adult	0.000
Fold4.Rep08	Rep08	Fold4	SVM	Adult	Pediatric	1.000
Fold4.Rep08	Rep08	Fold4	SVM	Pediatric	Pediatric	0.603
Fold4.Rep08	Rep08	Fold4	SVM	Pediatric	Pediatric	0.597
Fold4.Rep08	Rep08	Fold4	SVM	Pediatric	Pediatric	0.690
Fold4.Rep08	Rep08	Fold4	SVM	Pediatric	Pediatric	0.667
Fold4.Rep08	Rep08	Fold4	SVM	Adult	Adult	0.217
Fold4.Rep08	Rep08	Fold4	SVM	Adult	Adult	0.000
Fold4.Rep08	Rep08	Fold4	SVM	Adult	Adult	0.115
Fold4.Rep08	Rep08	Fold4	SVM	Adult	Adult	0.065
Fold5.Rep08	Rep08	Fold5	RM	Adult	Adult	0.020
Fold5.Rep08	Rep08	Fold5	RM	Adult	Adult	0.086
Fold5.Rep08	Rep08	Fold5	RM	Adult	Adult	0.034
Fold5.Rep08	Rep08	Fold5	RM	Adult	Adult	0.014
Fold5.Rep08	Rep08	Fold5	RM	Adult	Adult	0.258
Fold5.Rep08	Rep08	Fold5	RM	Adult	Adult	0.310
Fold5.Rep08	Rep08	Fold5	RM	Pediatric	Pediatric	0.808
Fold5.Rep08	Rep08	Fold5	RM	Pediatric	Pediatric	0.756
Fold5.Rep08	Rep08	Fold5	RM	Pediatric	Pediatric	0.822
Fold5.Rep08	Rep08	Fold5	RM	Pediatric	Pediatric	0.630
Fold5.Rep08	Rep08	Fold5	RM	Pediatric	Pediatric	0.792
Fold5.Rep08	Rep08	Fold5	RM	Adult	Adult	0.178
Fold5.Rep08	Rep08	Fold5	RM	Adult	Adult	0.092
Fold5.Rep08	Rep08	Fold5	RM	Adult	Adult	0.150
Fold5.Rep08	Rep08	Fold5	GBM	Adult	Adult	0.011
Fold5.Rep08	Rep08	Fold5	GBM	Adult	Adult	0.013
Fold5.Rep08	Rep08	Fold5	GBM	Adult	Adult	0.003
Fold5.Rep08	Rep08	Fold5	GBM	Adult	Adult	0.003
Fold5.Rep08	Rep08	Fold5	GBM	Adult	Adult	0.011
Fold5.Rep08	Rep08	Fold5	GBM	Adult	Adult	0.013
Fold5.Rep08	Rep08	Fold5	GBM	Pediatric	Pediatric	0.971
Fold5.Rep08	Rep08	Fold5	GBM	Pediatric	Pediatric	0.968
Fold5.Rep08	Rep08	Fold5	GBM	Pediatric	Pediatric	0.955
Fold5.Rep08	Rep08	Fold5	GBM	Pediatric	Pediatric	0.795
Fold5.Rep08	Rep08	Fold5	GBM	Pediatric	Pediatric	0.945
Fold5.Rep08	Rep08	Fold5	GBM	Adult	Adult	0.005
Fold5.Rep08	Rep08	Fold5	GBM	Adult	Adult	0.003
Fold5.Rep08	Rep08	Fold5	GBM	Adult	Adult	0.017
Fold5.Rep08	Rep08	Fold5	SVM	Adult	Adult	0.003
Fold5.Rep08	Rep08	Fold5	SVM	Adult	Adult	0.120
Fold5.Rep08	Rep08	Fold5	SVM	Adult	Adult	0.029
Fold5.Rep08	Rep08	Fold5	SVM	Adult	Adult	0.006
Fold5.Rep08	Rep08	Fold5	SVM	Adult	Adult	0.005
Fold5.Rep08	Rep08	Fold5	SVM	Adult	Pediatric	0.972

Fold5.Rep08	Rep08	Fold5	SVM	Pediatric	Pediatric	0.849
Fold5.Rep08	Rep08	Fold5	SVM	Pediatric	Adult	0.349
Fold5.Rep08	Rep08	Fold5	SVM	Pediatric	Pediatric	0.820
Fold5.Rep08	Rep08	Fold5	SVM	Pediatric	Adult	0.338
Fold5.Rep08	Rep08	Fold5	SVM	Pediatric	Pediatric	0.951
Fold5.Rep08	Rep08	Fold5	SVM	Adult	Adult	0.011
Fold5.Rep08	Rep08	Fold5	SVM	Adult	Adult	0.167
Fold5.Rep08	Rep08	Fold5	SVM	Adult	Adult	0.239
Fold1.Rep09	Rep09	Fold1	RM	Adult	Adult	0.008
Fold1.Rep09	Rep09	Fold1	RM	Adult	Adult	0.106
Fold1.Rep09	Rep09	Fold1	RM	Adult	Adult	0.194
Fold1.Rep09	Rep09	Fold1	RM	Adult	Adult	0.070
Fold1.Rep09	Rep09	Fold1	RM	Pediatric	Pediatric	0.924
Fold1.Rep09	Rep09	Fold1	RM	Pediatric	Pediatric	0.648
Fold1.Rep09	Rep09	Fold1	RM	Pediatric	Pediatric	0.858
Fold1.Rep09	Rep09	Fold1	RM	Pediatric	Pediatric	0.722
Fold1.Rep09	Rep09	Fold1	RM	Adult	Adult	0.176
Fold1.Rep09	Rep09	Fold1	RM	Adult	Adult	0.090
Fold1.Rep09	Rep09	Fold1	RM	Adult	Adult	0.054
Fold1.Rep09	Rep09	Fold1	RM	Adult	Adult	0.126
Fold1.Rep09	Rep09	Fold1	RM	Adult	Adult	0.144
Fold1.Rep09	Rep09	Fold1	GBM	Adult	Adult	0.000
Fold1.Rep09	Rep09	Fold1	GBM	Adult	Adult	0.000
Fold1.Rep09	Rep09	Fold1	GBM	Adult	Adult	0.000
Fold1.Rep09	Rep09	Fold1	GBM	Adult	Adult	0.000
Fold1.Rep09	Rep09	Fold1	GBM	Pediatric	Pediatric	1.000
Fold1.Rep09	Rep09	Fold1	GBM	Pediatric	Pediatric	1.000
Fold1.Rep09	Rep09	Fold1	GBM	Pediatric	Pediatric	1.000
Fold1.Rep09	Rep09	Fold1	GBM	Pediatric	Pediatric	0.999
Fold1.Rep09	Rep09	Fold1	GBM	Adult	Adult	0.000
Fold1.Rep09	Rep09	Fold1	GBM	Adult	Adult	0.000
Fold1.Rep09	Rep09	Fold1	GBM	Adult	Adult	0.000
Fold1.Rep09	Rep09	Fold1	GBM	Adult	Adult	0.000
Fold1.Rep09	Rep09	Fold1	GBM	Adult	Adult	0.000
Fold1.Rep09	Rep09	Fold1	SVM	Adult	Adult	0.149
Fold1.Rep09	Rep09	Fold1	SVM	Adult	Adult	0.149
Fold1.Rep09	Rep09	Fold1	SVM	Adult	Pediatric	1.000
Fold1.Rep09	Rep09	Fold1	SVM	Adult	Adult	0.000
Fold1.Rep09	Rep09	Fold1	SVM	Pediatric	Adult	0.356
Fold1.Rep09	Rep09	Fold1	SVM	Pediatric	Pediatric	0.510
Fold1.Rep09	Rep09	Fold1	SVM	Pediatric	Pediatric	0.528
Fold1.Rep09	Rep09	Fold1	SVM	Pediatric	Adult	0.298
Fold1.Rep09	Rep09	Fold1	SVM	Adult	Adult	0.078
Fold1.Rep09	Rep09	Fold1	SVM	Adult	Adult	0.123
Fold1.Rep09	Rep09	Fold1	SVM	Adult	Adult	0.019
Fold1.Rep09	Rep09	Fold1	SVM	Adult	Adult	0.091
Fold1.Rep09	Rep09	Fold1	SVM	Adult	Adult	0.046
Fold2.Rep09	Rep09	Fold2	RM	Adult	Adult	0.040
Fold2.Rep09	Rep09	Fold2	RM	Adult	Adult	0.104
Fold2.Rep09	Rep09	Fold2	RM	Adult	Adult	0.044
Fold2.Rep09	Rep09	Fold2	RM	Adult	Adult	0.092
Fold2.Rep09	Rep09	Fold2	RM	Adult	Adult	0.138
Fold2.Rep09	Rep09	Fold2	RM	Adult	Adult	0.270
Fold2.Rep09	Rep09	Fold2	RM	Adult	Adult	0.196
Fold2.Rep09	Rep09	Fold2	RM	Pediatric	Pediatric	0.750
Fold2.Rep09	Rep09	Fold2	RM	Pediatric	Pediatric	0.650
Fold2.Rep09	Rep09	Fold2	RM	Pediatric	Pediatric	0.828
Fold2.Rep09	Rep09	Fold2	RM	Pediatric	Pediatric	0.660
Fold2.Rep09	Rep09	Fold2	RM	Pediatric	Pediatric	0.718

Fold2.Rep09	Rep09	Fold2	RM	Adult	Adult	0.184
Fold2.Rep09	Rep09	Fold2	RM	Adult	Adult	0.126
Fold2.Rep09	Rep09	Fold2	RM	Adult	Adult	0.046
Fold2.Rep09	Rep09	Fold2	GBM	Adult	Adult	0.010
Fold2.Rep09	Rep09	Fold2	GBM	Adult	Adult	0.010
Fold2.Rep09	Rep09	Fold2	GBM	Adult	Adult	0.002
Fold2.Rep09	Rep09	Fold2	GBM	Adult	Adult	0.009
Fold2.Rep09	Rep09	Fold2	GBM	Adult	Adult	0.001
Fold2.Rep09	Rep09	Fold2	GBM	Adult	Adult	0.011
Fold2.Rep09	Rep09	Fold2	GBM	Adult	Adult	0.007
Fold2.Rep09	Rep09	Fold2	GBM	Pediatric	Pediatric	0.944
Fold2.Rep09	Rep09	Fold2	GBM	Pediatric	Pediatric	0.781
Fold2.Rep09	Rep09	Fold2	GBM	Pediatric	Pediatric	0.971
Fold2.Rep09	Rep09	Fold2	GBM	Pediatric	Pediatric	0.894
Fold2.Rep09	Rep09	Fold2	GBM	Pediatric	Pediatric	0.952
Fold2.Rep09	Rep09	Fold2	GBM	Adult	Adult	0.079
Fold2.Rep09	Rep09	Fold2	GBM	Adult	Adult	0.018
Fold2.Rep09	Rep09	Fold2	GBM	Adult	Adult	0.001
Fold2.Rep09	Rep09	Fold2	SVM	Adult	Adult	0.119
Fold2.Rep09	Rep09	Fold2	SVM	Adult	Pediatric	1.000
Fold2.Rep09	Rep09	Fold2	SVM	Adult	Adult	0.016
Fold2.Rep09	Rep09	Fold2	SVM	Adult	Adult	0.191
Fold2.Rep09	Rep09	Fold2	SVM	Adult	Adult	0.045
Fold2.Rep09	Rep09	Fold2	SVM	Adult	Adult	0.239
Fold2.Rep09	Rep09	Fold2	SVM	Adult	Adult	0.000
Fold2.Rep09	Rep09	Fold2	SVM	Pediatric	Pediatric	1.000
Fold2.Rep09	Rep09	Fold2	SVM	Pediatric	Adult	0.039
Fold2.Rep09	Rep09	Fold2	SVM	Pediatric	Adult	0.381
Fold2.Rep09	Rep09	Fold2	SVM	Pediatric	Adult	0.000
Fold2.Rep09	Rep09	Fold2	SVM	Pediatric	Pediatric	0.681
Fold2.Rep09	Rep09	Fold2	SVM	Adult	Adult	0.000
Fold2.Rep09	Rep09	Fold2	SVM	Adult	Adult	0.095
Fold2.Rep09	Rep09	Fold2	SVM	Adult	Adult	0.043
Fold3.Rep09	Rep09	Fold3	RM	Adult	Adult	0.018
Fold3.Rep09	Rep09	Fold3	RM	Adult	Adult	0.010
Fold3.Rep09	Rep09	Fold3	RM	Adult	Adult	0.132
Fold3.Rep09	Rep09	Fold3	RM	Adult	Adult	0.134
Fold3.Rep09	Rep09	Fold3	RM	Adult	Adult	0.152
Fold3.Rep09	Rep09	Fold3	RM	Pediatric	Pediatric	0.806
Fold3.Rep09	Rep09	Fold3	RM	Pediatric	Pediatric	0.854
Fold3.Rep09	Rep09	Fold3	RM	Pediatric	Pediatric	0.832
Fold3.Rep09	Rep09	Fold3	RM	Pediatric	Pediatric	0.778
Fold3.Rep09	Rep09	Fold3	RM	Adult	Adult	0.076
Fold3.Rep09	Rep09	Fold3	RM	Adult	Adult	0.240
Fold3.Rep09	Rep09	Fold3	RM	Adult	Adult	0.172
Fold3.Rep09	Rep09	Fold3	RM	Adult	Adult	0.120
Fold3.Rep09	Rep09	Fold3	GBM	Adult	Adult	0.008
Fold3.Rep09	Rep09	Fold3	GBM	Adult	Adult	0.008
Fold3.Rep09	Rep09	Fold3	GBM	Adult	Adult	0.004
Fold3.Rep09	Rep09	Fold3	GBM	Adult	Adult	0.012
Fold3.Rep09	Rep09	Fold3	GBM	Adult	Adult	0.009
Fold3.Rep09	Rep09	Fold3	GBM	Pediatric	Pediatric	0.950
Fold3.Rep09	Rep09	Fold3	GBM	Pediatric	Pediatric	0.978
Fold3.Rep09	Rep09	Fold3	GBM	Pediatric	Pediatric	0.939
Fold3.Rep09	Rep09	Fold3	GBM	Pediatric	Pediatric	0.960
Fold3.Rep09	Rep09	Fold3	GBM	Adult	Adult	0.004
Fold3.Rep09	Rep09	Fold3	GBM	Adult	Adult	0.038
Fold3.Rep09	Rep09	Fold3	GBM	Adult	Adult	0.002
Fold3.Rep09	Rep09	Fold3	GBM	Adult	Adult	0.010

Fold3.Rep09	Rep09	Fold3	SVM	Adult	Adult	0.141
Fold3.Rep09	Rep09	Fold3	SVM	Adult	Adult	0.019
Fold3.Rep09	Rep09	Fold3	SVM	Adult	Adult	0.084
Fold3.Rep09	Rep09	Fold3	SVM	Adult	Pediatric	0.825
Fold3.Rep09	Rep09	Fold3	SVM	Adult	Adult	0.000
Fold3.Rep09	Rep09	Fold3	SVM	Pediatric	Adult	0.403
Fold3.Rep09	Rep09	Fold3	SVM	Pediatric	Pediatric	0.987
Fold3.Rep09	Rep09	Fold3	SVM	Pediatric	Pediatric	0.825
Fold3.Rep09	Rep09	Fold3	SVM	Pediatric	Pediatric	0.519
Fold3.Rep09	Rep09	Fold3	SVM	Adult	Adult	0.158
Fold3.Rep09	Rep09	Fold3	SVM	Adult	Adult	0.054
Fold3.Rep09	Rep09	Fold3	SVM	Adult	Adult	0.011
Fold3.Rep09	Rep09	Fold3	SVM	Adult	Adult	0.136
Fold4.Rep09	Rep09	Fold4	RM	Adult	Adult	0.022
Fold4.Rep09	Rep09	Fold4	RM	Adult	Adult	0.128
Fold4.Rep09	Rep09	Fold4	RM	Adult	Adult	0.034
Fold4.Rep09	Rep09	Fold4	RM	Adult	Adult	0.024
Fold4.Rep09	Rep09	Fold4	RM	Adult	Adult	0.032
Fold4.Rep09	Rep09	Fold4	RM	Adult	Adult	0.366
Fold4.Rep09	Rep09	Fold4	RM	Adult	Adult	0.260
Fold4.Rep09	Rep09	Fold4	RM	Adult	Adult	0.288
Fold4.Rep09	Rep09	Fold4	RM	Pediatric	Pediatric	0.866
Fold4.Rep09	Rep09	Fold4	RM	Pediatric	Pediatric	0.618
Fold4.Rep09	Rep09	Fold4	RM	Pediatric	Pediatric	0.700
Fold4.Rep09	Rep09	Fold4	RM	Pediatric	Pediatric	0.744
Fold4.Rep09	Rep09	Fold4	RM	Adult	Adult	0.052
Fold4.Rep09	Rep09	Fold4	RM	Adult	Adult	0.412
Fold4.Rep09	Rep09	Fold4	GBM	Adult	Adult	0.000
Fold4.Rep09	Rep09	Fold4	GBM	Adult	Adult	0.000
Fold4.Rep09	Rep09	Fold4	GBM	Adult	Adult	0.000
Fold4.Rep09	Rep09	Fold4	GBM	Adult	Adult	0.000
Fold4.Rep09	Rep09	Fold4	GBM	Adult	Adult	0.000
Fold4.Rep09	Rep09	Fold4	GBM	Adult	Adult	0.003
Fold4.Rep09	Rep09	Fold4	GBM	Adult	Adult	0.078
Fold4.Rep09	Rep09	Fold4	GBM	Adult	Adult	0.001
Fold4.Rep09	Rep09	Fold4	GBM	Pediatric	Pediatric	1.000
Fold4.Rep09	Rep09	Fold4	GBM	Pediatric	Pediatric	0.997
Fold4.Rep09	Rep09	Fold4	GBM	Pediatric	Pediatric	0.997
Fold4.Rep09	Rep09	Fold4	GBM	Pediatric	Pediatric	0.997
Fold4.Rep09	Rep09	Fold4	GBM	Adult	Adult	0.000
Fold4.Rep09	Rep09	Fold4	GBM	Adult	Pediatric	0.729
Fold4.Rep09	Rep09	Fold4	SVM	Adult	Adult	0.000
Fold4.Rep09	Rep09	Fold4	SVM	Adult	Adult	0.025
Fold4.Rep09	Rep09	Fold4	SVM	Adult	Adult	0.015
Fold4.Rep09	Rep09	Fold4	SVM	Adult	Adult	0.029
Fold4.Rep09	Rep09	Fold4	SVM	Adult	Adult	0.045
Fold4.Rep09	Rep09	Fold4	SVM	Adult	Pediatric	0.876
Fold4.Rep09	Rep09	Fold4	SVM	Adult	Pediatric	0.785
Fold4.Rep09	Rep09	Fold4	SVM	Adult	Pediatric	0.875
Fold4.Rep09	Rep09	Fold4	SVM	Pediatric	Pediatric	0.929
Fold4.Rep09	Rep09	Fold4	SVM	Pediatric	Pediatric	0.505
Fold4.Rep09	Rep09	Fold4	SVM	Pediatric	Pediatric	0.832
Fold4.Rep09	Rep09	Fold4	SVM	Pediatric	Pediatric	0.986
Fold4.Rep09	Rep09	Fold4	SVM	Adult	Adult	0.000
Fold4.Rep09	Rep09	Fold4	SVM	Adult	Adult	0.195
Fold5.Rep09	Rep09	Fold5	RM	Adult	Adult	0.040
Fold5.Rep09	Rep09	Fold5	RM	Adult	Adult	0.080
Fold5.Rep09	Rep09	Fold5	RM	Adult	Adult	0.026
Fold5.Rep09	Rep09	Fold5	RM	Adult	Adult	0.008

Fold5.Rep09	Rep09	Fold5	RM	Adult	Adult	0.044
Fold5.Rep09	Rep09	Fold5	RM	Adult	Adult	0.230
Fold5.Rep09	Rep09	Fold5	RM	Pediatric	Pediatric	0.772
Fold5.Rep09	Rep09	Fold5	RM	Pediatric	Pediatric	0.716
Fold5.Rep09	Rep09	Fold5	RM	Pediatric	Pediatric	0.754
Fold5.Rep09	Rep09	Fold5	RM	Pediatric	Pediatric	0.586
Fold5.Rep09	Rep09	Fold5	RM	Pediatric	Pediatric	0.818
Fold5.Rep09	Rep09	Fold5	RM	Adult	Adult	0.130
Fold5.Rep09	Rep09	Fold5	RM	Adult	Adult	0.212
Fold5.Rep09	Rep09	Fold5	RM	Adult	Adult	0.128
Fold5.Rep09	Rep09	Fold5	GBM	Adult	Adult	0.009
Fold5.Rep09	Rep09	Fold5	GBM	Adult	Adult	0.009
Fold5.Rep09	Rep09	Fold5	GBM	Adult	Adult	0.002
Fold5.Rep09	Rep09	Fold5	GBM	Adult	Adult	0.002
Fold5.Rep09	Rep09	Fold5	GBM	Adult	Adult	0.009
Fold5.Rep09	Rep09	Fold5	GBM	Adult	Adult	0.008
Fold5.Rep09	Rep09	Fold5	GBM	Pediatric	Pediatric	0.985
Fold5.Rep09	Rep09	Fold5	GBM	Pediatric	Pediatric	0.985
Fold5.Rep09	Rep09	Fold5	GBM	Pediatric	Pediatric	0.975
Fold5.Rep09	Rep09	Fold5	GBM	Pediatric	Pediatric	0.918
Fold5.Rep09	Rep09	Fold5	GBM	Pediatric	Pediatric	0.985
Fold5.Rep09	Rep09	Fold5	GBM	Adult	Adult	0.008
Fold5.Rep09	Rep09	Fold5	GBM	Adult	Adult	0.010
Fold5.Rep09	Rep09	Fold5	GBM	Adult	Adult	0.002
Fold5.Rep09	Rep09	Fold5	SVM	Adult	Adult	0.045
Fold5.Rep09	Rep09	Fold5	SVM	Adult	Adult	0.054
Fold5.Rep09	Rep09	Fold5	SVM	Adult	Adult	0.000
Fold5.Rep09	Rep09	Fold5	SVM	Adult	Adult	0.000
Fold5.Rep09	Rep09	Fold5	SVM	Adult	Adult	0.082
Fold5.Rep09	Rep09	Fold5	SVM	Adult	Adult	0.000
Fold5.Rep09	Rep09	Fold5	SVM	Pediatric	Adult	0.270
Fold5.Rep09	Rep09	Fold5	SVM	Pediatric	Adult	0.155
Fold5.Rep09	Rep09	Fold5	SVM	Pediatric	Pediatric	0.530
Fold5.Rep09	Rep09	Fold5	SVM	Pediatric	Adult	0.475
Fold5.Rep09	Rep09	Fold5	SVM	Pediatric	Pediatric	0.672
Fold5.Rep09	Rep09	Fold5	SVM	Adult	Adult	0.244
Fold5.Rep09	Rep09	Fold5	SVM	Adult	Adult	0.000
Fold5.Rep09	Rep09	Fold5	SVM	Adult	Adult	0.157
Fold1.Rep10	Rep10	Fold1	RM	Adult	Adult	0.014
Fold1.Rep10	Rep10	Fold1	RM	Adult	Adult	0.036
Fold1.Rep10	Rep10	Fold1	RM	Adult	Adult	0.126
Fold1.Rep10	Rep10	Fold1	RM	Adult	Adult	0.026
Fold1.Rep10	Rep10	Fold1	RM	Adult	Adult	0.354
Fold1.Rep10	Rep10	Fold1	RM	Adult	Adult	0.170
Fold1.Rep10	Rep10	Fold1	RM	Adult	Adult	0.182
Fold1.Rep10	Rep10	Fold1	RM	Pediatric	Pediatric	0.818
Fold1.Rep10	Rep10	Fold1	RM	Pediatric	Pediatric	0.830
Fold1.Rep10	Rep10	Fold1	RM	Pediatric	Pediatric	0.620
Fold1.Rep10	Rep10	Fold1	RM	Pediatric	Pediatric	0.710
Fold1.Rep10	Rep10	Fold1	RM	Adult	Adult	0.156
Fold1.Rep10	Rep10	Fold1	RM	Adult	Adult	0.058
Fold1.Rep10	Rep10	Fold1	RM	Adult	Adult	0.248
Fold1.Rep10	Rep10	Fold1	GBM	Adult	Adult	0.005
Fold1.Rep10	Rep10	Fold1	GBM	Adult	Adult	0.005
Fold1.Rep10	Rep10	Fold1	GBM	Adult	Adult	0.009
Fold1.Rep10	Rep10	Fold1	GBM	Adult	Adult	0.003
Fold1.Rep10	Rep10	Fold1	GBM	Adult	Adult	0.023
Fold1.Rep10	Rep10	Fold1	GBM	Adult	Adult	0.002
Fold1.Rep10	Rep10	Fold1	GBM	Adult	Adult	0.007

Fold1.Rep10	Rep10	Fold1	GBM	Pediatric	Pediatric	0.983
Fold1.Rep10	Rep10	Fold1	GBM	Pediatric	Pediatric	0.965
Fold1.Rep10	Rep10	Fold1	GBM	Pediatric	Pediatric	0.922
Fold1.Rep10	Rep10	Fold1	GBM	Pediatric	Pediatric	0.983
Fold1.Rep10	Rep10	Fold1	GBM	Adult	Adult	0.009
Fold1.Rep10	Rep10	Fold1	GBM	Adult	Adult	0.002
Fold1.Rep10	Rep10	Fold1	GBM	Adult	Adult	0.019
Fold1.Rep10	Rep10	Fold1	SVM	Adult	Adult	0.097
Fold1.Rep10	Rep10	Fold1	SVM	Adult	Adult	0.194
Fold1.Rep10	Rep10	Fold1	SVM	Adult	Adult	0.139
Fold1.Rep10	Rep10	Fold1	SVM	Adult	Adult	0.119
Fold1.Rep10	Rep10	Fold1	SVM	Adult	Pediatric	0.540
Fold1.Rep10	Rep10	Fold1	SVM	Adult	Adult	0.157
Fold1.Rep10	Rep10	Fold1	SVM	Adult	Adult	0.154
Fold1.Rep10	Rep10	Fold1	SVM	Pediatric	Adult	0.313
Fold1.Rep10	Rep10	Fold1	SVM	Pediatric	Pediatric	0.555
Fold1.Rep10	Rep10	Fold1	SVM	Pediatric	Pediatric	0.711
Fold1.Rep10	Rep10	Fold1	SVM	Pediatric	Pediatric	0.879
Fold1.Rep10	Rep10	Fold1	SVM	Adult	Adult	0.000
Fold1.Rep10	Rep10	Fold1	SVM	Adult	Adult	0.000
Fold1.Rep10	Rep10	Fold1	SVM	Adult	Adult	0.057
Fold2.Rep10	Rep10	Fold2	RM	Adult	Adult	0.022
Fold2.Rep10	Rep10	Fold2	RM	Adult	Adult	0.122
Fold2.Rep10	Rep10	Fold2	RM	Pediatric	Pediatric	0.844
Fold2.Rep10	Rep10	Fold2	RM	Pediatric	Pediatric	0.734
Fold2.Rep10	Rep10	Fold2	RM	Pediatric	Pediatric	0.848
Fold2.Rep10	Rep10	Fold2	RM	Pediatric	Pediatric	0.858
Fold2.Rep10	Rep10	Fold2	RM	Pediatric	Pediatric	0.848
Fold2.Rep10	Rep10	Fold2	RM	Adult	Adult	0.160
Fold2.Rep10	Rep10	Fold2	RM	Adult	Adult	0.260
Fold2.Rep10	Rep10	Fold2	RM	Adult	Adult	0.408
Fold2.Rep10	Rep10	Fold2	RM	Adult	Adult	0.058
Fold2.Rep10	Rep10	Fold2	RM	Adult	Adult	0.166
Fold2.Rep10	Rep10	Fold2	RM	Adult	Adult	0.086
Fold2.Rep10	Rep10	Fold2	RM	Adult	Adult	0.166
Fold2.Rep10	Rep10	Fold2	GBM	Adult	Adult	0.000
Fold2.Rep10	Rep10	Fold2	GBM	Adult	Adult	0.000
Fold2.Rep10	Rep10	Fold2	GBM	Pediatric	Pediatric	1.000
Fold2.Rep10	Rep10	Fold2	GBM	Pediatric	Pediatric	1.000
Fold2.Rep10	Rep10	Fold2	GBM	Pediatric	Pediatric	1.000
Fold2.Rep10	Rep10	Fold2	GBM	Pediatric	Pediatric	1.000
Fold2.Rep10	Rep10	Fold2	GBM	Pediatric	Pediatric	1.000
Fold2.Rep10	Rep10	Fold2	GBM	Adult	Adult	0.000
Fold2.Rep10	Rep10	Fold2	GBM	Adult	Adult	0.000
Fold2.Rep10	Rep10	Fold2	GBM	Adult	Adult	0.113
Fold2.Rep10	Rep10	Fold2	GBM	Adult	Adult	0.000
Fold2.Rep10	Rep10	Fold2	GBM	Adult	Adult	0.000
Fold2.Rep10	Rep10	Fold2	GBM	Adult	Adult	0.000
Fold2.Rep10	Rep10	Fold2	GBM	Adult	Adult	0.000
Fold2.Rep10	Rep10	Fold2	SVM	Adult	Adult	0.000
Fold2.Rep10	Rep10	Fold2	SVM	Adult	Adult	0.259
Fold2.Rep10	Rep10	Fold2	SVM	Pediatric	Pediatric	0.669
Fold2.Rep10	Rep10	Fold2	SVM	Pediatric	Adult	0.204
Fold2.Rep10	Rep10	Fold2	SVM	Pediatric	Pediatric	0.746
Fold2.Rep10	Rep10	Fold2	SVM	Pediatric	Pediatric	0.522
Fold2.Rep10	Rep10	Fold2	SVM	Pediatric	Adult	0.474
Fold2.Rep10	Rep10	Fold2	SVM	Adult	Adult	0.195
Fold2.Rep10	Rep10	Fold2	SVM	Adult	Adult	0.000
Fold2.Rep10	Rep10	Fold2	SVM	Adult	Adult	0.438

Fold2.Rep10	Rep10	Fold2	SVM	Adult	Adult	0.005
Fold2.Rep10	Rep10	Fold2	SVM	Adult	Adult	0.035
Fold2.Rep10	Rep10	Fold2	SVM	Adult	Adult	0.025
Fold2.Rep10	Rep10	Fold2	SVM	Adult	Adult	0.249
Fold3.Rep10	Rep10	Fold3	RM	Adult	Adult	0.010
Fold3.Rep10	Rep10	Fold3	RM	Adult	Adult	0.142
Fold3.Rep10	Rep10	Fold3	RM	Adult	Adult	0.010
Fold3.Rep10	Rep10	Fold3	RM	Adult	Adult	0.148
Fold3.Rep10	Rep10	Fold3	RM	Adult	Adult	0.140
Fold3.Rep10	Rep10	Fold3	RM	Pediatric	Pediatric	0.836
Fold3.Rep10	Rep10	Fold3	RM	Pediatric	Pediatric	0.606
Fold3.Rep10	Rep10	Fold3	RM	Pediatric	Pediatric	0.656
Fold3.Rep10	Rep10	Fold3	RM	Pediatric	Pediatric	0.604
Fold3.Rep10	Rep10	Fold3	RM	Adult	Adult	0.174
Fold3.Rep10	Rep10	Fold3	RM	Adult	Adult	0.050
Fold3.Rep10	Rep10	Fold3	RM	Adult	Adult	0.068
Fold3.Rep10	Rep10	Fold3	RM	Adult	Adult	0.100
Fold3.Rep10	Rep10	Fold3	GBM	Adult	Adult	0.009
Fold3.Rep10	Rep10	Fold3	GBM	Adult	Adult	0.003
Fold3.Rep10	Rep10	Fold3	GBM	Adult	Adult	0.002
Fold3.Rep10	Rep10	Fold3	GBM	Adult	Adult	0.004
Fold3.Rep10	Rep10	Fold3	GBM	Adult	Adult	0.002
Fold3.Rep10	Rep10	Fold3	GBM	Pediatric	Pediatric	0.986
Fold3.Rep10	Rep10	Fold3	GBM	Pediatric	Pediatric	0.904
Fold3.Rep10	Rep10	Fold3	GBM	Pediatric	Pediatric	0.966
Fold3.Rep10	Rep10	Fold3	GBM	Pediatric	Pediatric	0.898
Fold3.Rep10	Rep10	Fold3	GBM	Adult	Adult	0.004
Fold3.Rep10	Rep10	Fold3	GBM	Adult	Adult	0.002
Fold3.Rep10	Rep10	Fold3	GBM	Adult	Adult	0.002
Fold3.Rep10	Rep10	Fold3	GBM	Adult	Adult	0.002
Fold3.Rep10	Rep10	Fold3	SVM	Adult	Adult	0.184
Fold3.Rep10	Rep10	Fold3	SVM	Adult	Adult	0.152
Fold3.Rep10	Rep10	Fold3	SVM	Adult	Adult	0.132
Fold3.Rep10	Rep10	Fold3	SVM	Adult	Pediatric	0.801
Fold3.Rep10	Rep10	Fold3	SVM	Adult	Adult	0.000
Fold3.Rep10	Rep10	Fold3	SVM	Pediatric	Adult	0.481
Fold3.Rep10	Rep10	Fold3	SVM	Pediatric	Pediatric	0.500
Fold3.Rep10	Rep10	Fold3	SVM	Pediatric	Adult	0.497
Fold3.Rep10	Rep10	Fold3	SVM	Pediatric	Adult	0.407
Fold3.Rep10	Rep10	Fold3	SVM	Adult	Adult	0.122
Fold3.Rep10	Rep10	Fold3	SVM	Adult	Adult	0.273
Fold3.Rep10	Rep10	Fold3	SVM	Adult	Adult	0.497
Fold3.Rep10	Rep10	Fold3	SVM	Adult	Adult	0.067
Fold4.Rep10	Rep10	Fold4	RM	Adult	Adult	0.026
Fold4.Rep10	Rep10	Fold4	RM	Adult	Adult	0.126
Fold4.Rep10	Rep10	Fold4	RM	Adult	Adult	0.026
Fold4.Rep10	Rep10	Fold4	RM	Adult	Adult	0.014
Fold4.Rep10	Rep10	Fold4	RM	Adult	Adult	0.030
Fold4.Rep10	Rep10	Fold4	RM	Adult	Adult	0.290
Fold4.Rep10	Rep10	Fold4	RM	Adult	Adult	0.148
Fold4.Rep10	Rep10	Fold4	RM	Adult	Adult	0.260
Fold4.Rep10	Rep10	Fold4	RM	Adult	Adult	0.178
Fold4.Rep10	Rep10	Fold4	RM	Pediatric	Pediatric	0.842
Fold4.Rep10	Rep10	Fold4	RM	Pediatric	Pediatric	0.690
Fold4.Rep10	Rep10	Fold4	RM	Pediatric	Pediatric	0.824
Fold4.Rep10	Rep10	Fold4	RM	Pediatric	Pediatric	0.826
Fold4.Rep10	Rep10	Fold4	GBM	Adult	Adult	0.004
Fold4.Rep10	Rep10	Fold4	GBM	Adult	Adult	0.004
Fold4.Rep10	Rep10	Fold4	GBM	Adult	Adult	0.004

Fold4.Rep10	Rep10	Fold4	GBM	Adult	Adult	0.002
Fold4.Rep10	Rep10	Fold4	GBM	Adult	Adult	0.004
Fold4.Rep10	Rep10	Fold4	GBM	Adult	Adult	0.022
Fold4.Rep10	Rep10	Fold4	GBM	Adult	Adult	0.004
Fold4.Rep10	Rep10	Fold4	GBM	Adult	Adult	0.029
Fold4.Rep10	Rep10	Fold4	GBM	Adult	Adult	0.004
Fold4.Rep10	Rep10	Fold4	GBM	Pediatric	Pediatric	0.971
Fold4.Rep10	Rep10	Fold4	GBM	Pediatric	Pediatric	0.971
Fold4.Rep10	Rep10	Fold4	GBM	Pediatric	Pediatric	0.982
Fold4.Rep10	Rep10	Fold4	GBM	Pediatric	Pediatric	0.960
Fold4.Rep10	Rep10	Fold4	SVM	Adult	Adult	0.001
Fold4.Rep10	Rep10	Fold4	SVM	Adult	Adult	0.159
Fold4.Rep10	Rep10	Fold4	SVM	Adult	Adult	0.023
Fold4.Rep10	Rep10	Fold4	SVM	Adult	Adult	0.016
Fold4.Rep10	Rep10	Fold4	SVM	Adult	Adult	0.062
Fold4.Rep10	Rep10	Fold4	SVM	Adult	Adult	0.046
Fold4.Rep10	Rep10	Fold4	SVM	Adult	Adult	0.000
Fold4.Rep10	Rep10	Fold4	SVM	Adult	Adult	0.000
Fold4.Rep10	Rep10	Fold4	SVM	Adult	Pediatric	1.000
Fold4.Rep10	Rep10	Fold4	SVM	Pediatric	Pediatric	0.862
Fold4.Rep10	Rep10	Fold4	SVM	Pediatric	Adult	0.476
Fold4.Rep10	Rep10	Fold4	SVM	Pediatric	Pediatric	0.657
Fold4.Rep10	Rep10	Fold4	SVM	Pediatric	Pediatric	0.942
Fold5.Rep10	Rep10	Fold5	RM	Adult	Adult	0.038
Fold5.Rep10	Rep10	Fold5	RM	Adult	Adult	0.104
Fold5.Rep10	Rep10	Fold5	RM	Adult	Adult	0.126
Fold5.Rep10	Rep10	Fold5	RM	Adult	Adult	0.070
Fold5.Rep10	Rep10	Fold5	RM	Adult	Adult	0.030
Fold5.Rep10	Rep10	Fold5	RM	Adult	Adult	0.200
Fold5.Rep10	Rep10	Fold5	RM	Adult	Adult	0.282
Fold5.Rep10	Rep10	Fold5	RM	Pediatric	Pediatric	0.814
Fold5.Rep10	Rep10	Fold5	RM	Pediatric	Pediatric	0.818
Fold5.Rep10	Rep10	Fold5	RM	Pediatric	Pediatric	0.872
Fold5.Rep10	Rep10	Fold5	RM	Pediatric	Pediatric	0.676
Fold5.Rep10	Rep10	Fold5	RM	Pediatric	Pediatric	0.692
Fold5.Rep10	Rep10	Fold5	RM	Adult	Adult	0.090
Fold5.Rep10	Rep10	Fold5	RM	Adult	Adult	0.148
Fold5.Rep10	Rep10	Fold5	RM	Adult	Adult	0.128
Fold5.Rep10	Rep10	Fold5	GBM	Adult	Adult	0.013
Fold5.Rep10	Rep10	Fold5	GBM	Adult	Adult	0.017
Fold5.Rep10	Rep10	Fold5	GBM	Adult	Adult	0.012
Fold5.Rep10	Rep10	Fold5	GBM	Adult	Adult	0.017
Fold5.Rep10	Rep10	Fold5	GBM	Adult	Adult	0.002
Fold5.Rep10	Rep10	Fold5	GBM	Adult	Adult	0.192
Fold5.Rep10	Rep10	Fold5	GBM	Adult	Adult	0.024
Fold5.Rep10	Rep10	Fold5	GBM	Pediatric	Pediatric	0.980
Fold5.Rep10	Rep10	Fold5	GBM	Pediatric	Pediatric	0.966
Fold5.Rep10	Rep10	Fold5	GBM	Pediatric	Pediatric	0.969
Fold5.Rep10	Rep10	Fold5	GBM	Pediatric	Pediatric	0.945
Fold5.Rep10	Rep10	Fold5	GBM	Pediatric	Pediatric	0.942
Fold5.Rep10	Rep10	Fold5	GBM	Adult	Adult	0.006
Fold5.Rep10	Rep10	Fold5	GBM	Adult	Adult	0.013
Fold5.Rep10	Rep10	Fold5	GBM	Adult	Adult	0.069
Fold5.Rep10	Rep10	Fold5	SVM	Adult	Adult	0.010
Fold5.Rep10	Rep10	Fold5	SVM	Adult	Adult	0.053
Fold5.Rep10	Rep10	Fold5	SVM	Adult	Adult	0.015
Fold5.Rep10	Rep10	Fold5	SVM	Adult	Adult	0.060
Fold5.Rep10	Rep10	Fold5	SVM	Adult	Adult	0.001
Fold5.Rep10	Rep10	Fold5	SVM	Adult	Pediatric	0.642

Fold5.Rep10	Rep10	Fold5	SVM	Adult	Pediatric	0.808
Fold5.Rep10	Rep10	Fold5	SVM	Pediatric	Adult	0.296
Fold5.Rep10	Rep10	Fold5	SVM	Pediatric	Pediatric	1.000
Fold5.Rep10	Rep10	Fold5	SVM	Pediatric	Pediatric	0.813
Fold5.Rep10	Rep10	Fold5	SVM	Pediatric	Pediatric	0.593
Fold5.Rep10	Rep10	Fold5	SVM	Pediatric	Pediatric	0.834
Fold5.Rep10	Rep10	Fold5	SVM	Adult	Adult	0.017
Fold5.Rep10	Rep10	Fold5	SVM	Adult	Adult	0.326
Fold5.Rep10	Rep10	Fold5	SVM	Adult	Adult	0.001

NestedCV_Summary	
**Method**	**Pooled_AUC**
GBM	1
Random Forest	1
SVM	0.869274662

NestedCV_Summary				
**Outer Resample**	**Outer Repea**	**Outer Fold**	**Method**	**BestTune**
Fold1.Rep01	Rep01	Fold1	RM	mtry=2
Fold1.Rep01	Rep01	Fold1	GBM	n.trees=50;
Fold1.Rep01	Rep01	Fold1	SVM	C=1
Fold2.Rep01	Rep01	Fold2	RM	mtry=2
Fold2.Rep01	Rep01	Fold2	GBM	n.trees=50;
Fold2.Rep01	Rep01	Fold2	SVM	C=1
Fold3.Rep01	Rep01	Fold3	RM	mtry=2
Fold3.Rep01	Rep01	Fold3	GBM	n.trees=50;
Fold3.Rep01	Rep01	Fold3	SVM	C=1
Fold4.Rep01	Rep01	Fold4	RM	mtry=2
Fold4.Rep01	Rep01	Fold4	GBM	n.trees=100;
Fold4.Rep01	Rep01	Fold4	SVM	C=1
Fold5.Rep01	Rep01	Fold5	RM	mtry=2
Fold5.Rep01	Rep01	Fold5	GBM	n.trees=50;
Fold5.Rep01	Rep01	Fold5	SVM	C=1
Fold1.Rep02	Rep02	Fold1	RM	mtry=2
Fold1.Rep02	Rep02	Fold1	GBM	n.trees=100;
Fold1.Rep02	Rep02	Fold1	SVM	C=1
Fold2.Rep02	Rep02	Fold2	RM	mtry=2
Fold2.Rep02	Rep02	Fold2	GBM	n.trees=50;
Fold2.Rep02	Rep02	Fold2	SVM	C=1
Fold3.Rep02	Rep02	Fold3	RM	mtry=2
Fold3.Rep02	Rep02	Fold3	GBM	n.trees=50;
Fold3.Rep02	Rep02	Fold3	SVM	C=1
Fold4.Rep02	Rep02	Fold4	RM	mtry=2
Fold4.Rep02	Rep02	Fold4	GBM	n.trees=50;
Fold4.Rep02	Rep02	Fold4	SVM	C=1
Fold5.Rep02	Rep02	Fold5	RM	mtry=2
Fold5.Rep02	Rep02	Fold5	GBM	n.trees=50;
Fold5.Rep02	Rep02	Fold5	SVM	C=1
Fold1.Rep03	Rep03	Fold1	RM	mtry=2
Fold1.Rep03	Rep03	Fold1	GBM	n.trees=50;
Fold1.Rep03	Rep03	Fold1	SVM	C=1
Fold2.Rep03	Rep03	Fold2	RM	mtry=2
Fold2.Rep03	Rep03	Fold2	GBM	n.trees=50;
Fold2.Rep03	Rep03	Fold2	SVM	C=1
Fold3.Rep03	Rep03	Fold3	RM	mtry=2
Fold3.Rep03	Rep03	Fold3	GBM	n.trees=100;
Fold3.Rep03	Rep03	Fold3	SVM	C=1
Fold4.Rep03	Rep03	Fold4	RM	mtry=2
Fold4.Rep03	Rep03	Fold4	GBM	n.trees=50;
Fold4.Rep03	Rep03	Fold4	SVM	C=1
Fold5.Rep03	Rep03	Fold5	RM	mtry=2
Fold5.Rep03	Rep03	Fold5	GBM	n.trees=50;
Fold5.Rep03	Rep03	Fold5	SVM	C=1
Fold1.Rep04	Rep04	Fold1	RM	mtry=2
Fold1.Rep04	Rep04	Fold1	GBM	n.trees=50;
Fold1.Rep04	Rep04	Fold1	SVM	C=1
Fold2.Rep04	Rep04	Fold2	RM	mtry=2
Fold2.Rep04	Rep04	Fold2	GBM	n.trees=50;
Fold2.Rep04	Rep04	Fold2	SVM	C=1
Fold3.Rep04	Rep04	Fold3	RM	mtry=2
Fold3.Rep04	Rep04	Fold3	GBM	n.trees=50;
Fold3.Rep04	Rep04	Fold3	SVM	C=1
Fold4.Rep04	Rep04	Fold4	RM	mtry=2
Fold4.Rep04	Rep04	Fold4	GBM	n.trees=100;
Fold4.Rep04	Rep04	Fold4	SVM	C=1

Fold5.Rep04	Rep04	Fold5	RM	mtry=2
Fold5.Rep04	Rep04	Fold5	GBM	n.trees=50;
Fold5.Rep04	Rep04	Fold5	SVM	C=1
Fold1.Rep05	Rep05	Fold1	RM	mtry=2
Fold1.Rep05	Rep05	Fold1	GBM	n.trees=50;
Fold1.Rep05	Rep05	Fold1	SVM	C=1
Fold2.Rep05	Rep05	Fold2	RM	mtry=2
Fold2.Rep05	Rep05	Fold2	GBM	n.trees=50;
Fold2.Rep05	Rep05	Fold2	SVM	C=1
Fold3.Rep05	Rep05	Fold3	RM	mtry=2
Fold3.Rep05	Rep05	Fold3	GBM	n.trees=50;
Fold3.Rep05	Rep05	Fold3	SVM	C=1
Fold4.Rep05	Rep05	Fold4	RM	mtry=2
Fold4.Rep05	Rep05	Fold4	GBM	n.trees=50;
Fold4.Rep05	Rep05	Fold4	SVM	C=1
Fold5.Rep05	Rep05	Fold5	RM	mtry=2
Fold5.Rep05	Rep05	Fold5	GBM	n.trees=50;
Fold5.Rep05	Rep05	Fold5	SVM	C=1
Fold1.Rep06	Rep06	Fold1	RM	mtry=2
Fold1.Rep06	Rep06	Fold1	GBM	n.trees=50;
Fold1.Rep06	Rep06	Fold1	SVM	C=1
Fold2.Rep06	Rep06	Fold2	RM	mtry=2
Fold2.Rep06	Rep06	Fold2	GBM	n.trees=50;
Fold2.Rep06	Rep06	Fold2	SVM	C=1
Fold3.Rep06	Rep06	Fold3	RM	mtry=2
Fold3.Rep06	Rep06	Fold3	GBM	n.trees=150;
Fold3.Rep06	Rep06	Fold3	SVM	C=1
Fold4.Rep06	Rep06	Fold4	RM	mtry=2
Fold4.Rep06	Rep06	Fold4	GBM	n.trees=50;
Fold4.Rep06	Rep06	Fold4	SVM	C=1
Fold5.Rep06	Rep06	Fold5	RM	mtry=2
Fold5.Rep06	Rep06	Fold5	GBM	n.trees=50;
Fold5.Rep06	Rep06	Fold5	SVM	C=1
Fold1.Rep07	Rep07	Fold1	RM	mtry=2
Fold1.Rep07	Rep07	Fold1	GBM	n.trees=50;
Fold1.Rep07	Rep07	Fold1	SVM	C=1
Fold2.Rep07	Rep07	Fold2	RM	mtry=2
Fold2.Rep07	Rep07	Fold2	GBM	n.trees=50;
Fold2.Rep07	Rep07	Fold2	SVM	C=1
Fold3.Rep07	Rep07	Fold3	RM	mtry=2
Fold3.Rep07	Rep07	Fold3	GBM	n.trees=50;
Fold3.Rep07	Rep07	Fold3	SVM	C=1
Fold4.Rep07	Rep07	Fold4	RM	mtry=2
Fold4.Rep07	Rep07	Fold4	GBM	n.trees=50;
Fold4.Rep07	Rep07	Fold4	SVM	C=1
Fold5.Rep07	Rep07	Fold5	RM	mtry=2
Fold5.Rep07	Rep07	Fold5	GBM	n.trees=50;
Fold5.Rep07	Rep07	Fold5	SVM	C=1
Fold1.Rep08	Rep08	Fold1	RM	mtry=2
Fold1.Rep08	Rep08	Fold1	GBM	n.trees=50;
Fold1.Rep08	Rep08	Fold1	SVM	C=1
Fold2.Rep08	Rep08	Fold2	RM	mtry=2
Fold2.Rep08	Rep08	Fold2	GBM	n.trees=50;
Fold2.Rep08	Rep08	Fold2	SVM	C=1
Fold3.Rep08	Rep08	Fold3	RM	mtry=2
Fold3.Rep08	Rep08	Fold3	GBM	n.trees=50;
Fold3.Rep08	Rep08	Fold3	SVM	C=1
Fold4.Rep08	Rep08	Fold4	RM	mtry=2
Fold4.Rep08	Rep08	Fold4	GBM	n.trees=50;

Fold4.Rep08	Rep08	Fold4	SVM	C=1
Fold5.Rep08	Rep08	Fold5	RM	mtry=2
Fold5.Rep08	Rep08	Fold5	GBM	n.trees=50;
Fold5.Rep08	Rep08	Fold5	SVM	C=1
Fold1.Rep09	Rep09	Fold1	RM	mtry=2
Fold1.Rep09	Rep09	Fold1	GBM	n.trees=150;
Fold1.Rep09	Rep09	Fold1	SVM	C=1
Fold2.Rep09	Rep09	Fold2	RM	mtry=2
Fold2.Rep09	Rep09	Fold2	GBM	n.trees=50;
Fold2.Rep09	Rep09	Fold2	SVM	C=1
Fold3.Rep09	Rep09	Fold3	RM	mtry=2
Fold3.Rep09	Rep09	Fold3	GBM	n.trees=50;
Fold3.Rep09	Rep09	Fold3	SVM	C=1
Fold4.Rep09	Rep09	Fold4	RM	mtry=2
Fold4.Rep09	Rep09	Fold4	GBM	n.trees=100;
Fold4.Rep09	Rep09	Fold4	SVM	C=1
Fold5.Rep09	Rep09	Fold5	RM	mtry=2
Fold5.Rep09	Rep09	Fold5	GBM	n.trees=50;
Fold5.Rep09	Rep09	Fold5	SVM	C=1
Fold1.Rep10	Rep10	Fold1	RM	mtry=2
Fold1.Rep10	Rep10	Fold1	GBM	n.trees=50;
Fold1.Rep10	Rep10	Fold1	SVM	C=1
Fold2.Rep10	Rep10	Fold2	RM	mtry=2
Fold2.Rep10	Rep10	Fold2	GBM	n.trees=150;
Fold2.Rep10	Rep10	Fold2	SVM	C=1
Fold3.Rep10	Rep10	Fold3	RM	mtry=2
Fold3.Rep10	Rep10	Fold3	GBM	n.trees=50;
Fold3.Rep10	Rep10	Fold3	SVM	C=1
Fold4.Rep10	Rep10	Fold4	RM	mtry=2
Fold4.Rep10	Rep10	Fold4	GBM	n.trees=50;
Fold4.Rep10	Rep10	Fold4	SVM	C=1
Fold5.Rep10	Rep10	Fold5	RM	mtry=2
Fold5.Rep10	Rep10	Fold5	GBM	n.trees=50;
Fold5.Rep10	Rep10	Fold5	SVM	C=1

Importance	
**RF_Importance**	
**Genus**	**Overall**
Clavispora	100
Aureobasidium	54.5750617
Cladosporium	51.26593628
Chytridiomycota_gen_Incertae_sedis	40.77820004
Vishniacozyma	27.46584667
Cutaneotrichosporon	23.05860617
Saccharomycetes_gen_Incertae_sedis	20.82665502
Aphelidium	15.78266592
Betamyces	15.39905897
Neodevriesiaceae_gen_Incertae_sedis	15.30044195
**GBM_Importance**	
**Genus**	**Overall**
Clavispora	100
Cladosporium	0.050835607
Cutaneotrichosporon	0.001733505
Chytridiomycota_gen_Incertae_sedis	0.00079186
Fungi_gen_Incertae_sedis	0
Cryptococcus	0
Ascomycota	0
Vishniacozyma	0
Hyphodontia	0
Malassezia	0
**SVM_Importance**	
**Genus**	**Overall**
Clavispora	100
Aureobasidium	76.23762376
Chytridiomycota_gen_Incertae_sedis	73.56435644
Cladosporium	69.9009901
Vishniacozyma	56.43564356
Cutaneotrichosporon	51.68316832
Betamyces	45.54455446
Saccharomycetes_gen_Incertae_sedis	41.58415842
Aphelidium	40.3960396
Fusicolla	34.65346535
