# Supplementary material for: Ecological patterns of the gut mycobiome and microbiome in ulcerative colitis across life stages
Source: Front Cell Infect Microbiol. 2026 Apr 28;16:1769892. doi: 10.3389/fcimb.2026.1769892 (PMC13161133; doi:10.3389/fcimb.2026.1769892)
Supplement: Supplementary file 3 [file DataSheet3.pdf]

feature	metadata	coef	stderr	pval	qval
Neodevriesiaceae_gen_Incertae_sedis	CategorigalAge	-5.750	1.070	0.00000	0.00043
Cladosporium	CategorigalAge	-5.599	1.077	0.00000	0.00043
Clavispora	CategorigalAge	7.245	1.659	0.00005	0.00561
Betamyces	immuno_therapy	4.179	0.978	0.00007	0.00580
Neodevriesiaceae_gen_Incertae_sedis	BMI	1.645	0.449	0.00054	0.03463
Chytridiomycota_gen_Incertae_sedis	CategorigalAge	5.516	1.627	0.00127	0.06753
Aphelidium	immuno_therapy	4.037	1.255	0.00213	0.08515
Betamyces	CategorigalAge	4.501	1.387	0.00196	0.08515
Saccharomycetes_gen_Incertae_sedis	immuno_therapy	2.715	0.936	0.00525	0.18676
Aphelidium	CategorigalAge	4.965	1.781	0.00716	0.22920
Aureobasidium	CategorigalAge	-4.177	1.537	0.00864	0.25144
Vishniacozyma	immuno_therapy	-3.198	1.264	0.01412	0.37648
Rozellomycota_gen_Incertae_sedis	Disease_duration	-0.770	0.319	0.01896	0.46662
Fungi_gen_Incertae_sedis	BMI	1.353	0.581	0.02337	0.46978
Fusicolla	CategorigalAge	2.441	1.036	0.02181	0.46978
Neodevriesiaceae_gen_Incertae_sedis	biologic_therapy	-1.908	0.820	0.02349	0.46978
Clavispora	5-ASA	-2.720	1.196	0.02670	0.47473
Kurtzmaniella	biologic_therapy	-2.616	1.146	0.02612	0.47473
Clavispora	immuno_therapy	-2.614	1.169	0.02922	0.48363
Neodevriesiaceae_gen_Incertae_sedis	MAYO	3.235	1.456	0.03023	0.48363
Ascomycota	immuno_therapy	2.525	1.166	0.03453	0.48549
Cutaneotrichosporon	CategorigalAge	3.554	1.623	0.03256	0.48549
Botrytis	BMI	-0.768	0.356	0.03489	0.48549
Vishniacozyma	5-ASA	-2.611	1.293	0.04804	0.56771
Vishniacozyma	CategorigalAge	3.616	1.793	0.04832	0.56771
Fusicolla	Disease_duration	-0.681	0.338	0.04848	0.56771
Wickerhamomyces	CategorigalAge	2.875	1.410	0.04606	0.56771
Alternaria	BMI	1.775	0.886	0.04967	0.56771
Candida	BMI	-1.165	0.607	0.05964	0.65814
Aureobasidium	MAYO	3.938	2.091	0.06469	0.68999
Exophiala	biologic_therapy	-2.058	1.102	0.06685	0.69007
Fungi_gen_Incertae_sedis	Disease_duration	-0.818	0.451	0.07516	0.74121
Neodevriesiaceae_gen_Incertae_sedis	Steroids	-1.374	0.762	0.07644	0.74121
Aureobasidium	Steroids	-1.940	1.094	0.08141	0.74436
Wickerhamomyces	biologic_therapy	-1.924	1.081	0.08025	0.74436
Aureobasidium	5-ASA	1.928	1.108	0.08723	0.74557
Aphelidium	biologic_therapy	2.364	1.365	0.08854	0.74557
Chytridiomycota_gen_Incertae_sedis	5-ASA	-2.053	1.174	0.08550	0.74557
Candida	Disease_duration	0.779	0.471	0.10377	0.76604
Fusicolla	5-ASA	1.259	0.747	0.09732	0.76604
Neodevriesiaceae_gen_Incertae_sedis	MAYO	2.281	1.377	0.10308	0.76604
Aureobasidium	MAYO	3.322	2.010	0.10375	0.76604
Aureobasidium	immuno_therapy	-1.782	1.083	0.10533	0.76604
Rhizophydiales_gen_Incertae_sedis	BMI	0.607	0.365	0.10228	0.76604
Aureobasidium	BMI	-1.053	0.645	0.10799	0.76790
Saccharomycetes_gen_Incertae_sedis	CategorigalAge	-2.131	1.328	0.11397	0.79284
Cladosporium	Steroids	1.162	0.766	0.13482	0.81130
Cladosporium	5-ASA	-1.223	0.776	0.12076	0.81130
Saccharomycetes	immuno_therapy	0.703	0.464	0.13483	0.81130

Chytridiomycota_gen_Incertae_sedis	immuno_therapy	-1.730	1.147	0.13691	0.81130
Rhizophydiales_gen_Incertae_sedis	Disease_duration	-0.431	0.284	0.13406	0.81130
Alternaria	immuno_therapy	2.276	1.488	0.13150	0.81130
Rhizophydium	immuno_therapy	-0.659	0.429	0.13026	0.81130
Betamyces	BMI	0.898	0.582	0.12838	0.81130
Fusicolla	Steroids	-1.094	0.737	0.14312	0.81131
Coniochaeta	immuno_therapy	-0.396	0.272	0.15098	0.81131
Saccharomycetes_gen_Incertae_sedis	MAYO	2.589	1.807	0.15719	0.81131
Saccharomycetes_gen_Incertae_sedis	5-ASA	1.375	0.958	0.15636	0.81131
Kurtzmaniella	CategorigalAge	2.211	1.495	0.14462	0.81131
Alternaria	MAYO	4.079	2.760	0.14476	0.81131
Rhizophydium	Disease_duration	-0.286	0.199	0.15491	0.81131
Rozellomycota_gen_Incertae_sedis	5-ASA	1.030	0.706	0.14983	0.81131
Rhizophydiales_gen_Incertae_sedis	5-ASA	-0.888	0.628	0.16286	0.82722
Cladosporium	Disease_duration	0.492	0.351	0.16627	0.83137
Saccharomycetes_gen_Incertae_sedis	BMI	0.771	0.557	0.17149	0.83648
Alternaria	CategorigalAge	-2.915	2.110	0.17252	0.83648
Ascomycota	CategorigalAge	2.182	1.655	0.19250	0.83974
Hyphodontia	MAYO	-1.958	1.562	0.21506	0.83974
Cutaneotrichosporon	Disease_duration	0.718	0.529	0.18019	0.83974
Malassezia	Disease_duration	-0.930	0.696	0.18663	0.83974
Malassezia	5-ASA	2.003	1.540	0.19843	0.83974
Clavispora	BMI	-0.874	0.696	0.21447	0.83974
Clavispora	biologic_therapy	-1.595	1.271	0.21465	0.83974
Candida	biologic_therapy	-1.399	1.108	0.21174	0.83974
Coniochaeta	CategorigalAge	0.526	0.386	0.17792	0.83974
Aphelidium	5-ASA	1.615	1.284	0.21356	0.83974
Debaryomyces	5-ASA	1.753	1.349	0.19912	0.83974
Saccharomycetes	5-ASA	0.634	0.475	0.18708	0.83974
Meyerozyma	biologic_therapy	-0.902	0.720	0.21518	0.83974
Alternaria	biologic_therapy	2.133	1.617	0.19250	0.83974
Dioszegia	Steroids	0.908	0.698	0.19848	0.83974
Rozellomycota_gen_Incertae_sedis	BMI	0.531	0.411	0.20076	0.83974
Ascomycota	5-ASA	1.405	1.193	0.24387	0.85642
Cutaneotrichosporon	BMI	0.822	0.681	0.23219	0.85642
Cutaneotrichosporon	Steroids	-1.327	1.155	0.25521	0.85642
Malassezia	MAYO	-3.441	2.905	0.24112	0.85642
Fusicolla	biologic_therapy	0.960	0.794	0.23122	0.85642
Botrytis	Steroids	-0.691	0.603	0.25693	0.85642
Neodevriesiaceae_gen_Incertae_sedis	5-ASA	-0.922	0.772	0.23730	0.85642
Debaryomyces	CategorigalAge	2.225	1.871	0.23927	0.85642
Saccharomycetes_gen_Incertae_sedis	Disease_duration	-0.505	0.433	0.24810	0.85642
Didymellaceae	MAYO	1.988	1.727	0.25437	0.85642
Rhizophydium	Steroids	-0.517	0.434	0.23852	0.85642
Dioszegia	Disease_duration	-0.378	0.320	0.24153	0.85642
Exophiala	5-ASA	-1.196	1.037	0.25343	0.85642
Exophiala	CategorigalAge	1.650	1.438	0.25580	0.85642
Nak5-ASAwaea	Steroids	-1.254	1.118	0.26686	0.87137
Kurtzmaniella	5-ASA	1.213	1.078	0.26509	0.87137
Hyphodontia	MAYO	-1.699	1.652	0.30814	0.89993

Cutaneotrichosporon	MAYO	2.248	2.122	0.29392	0.89993
Candida	MAYO	-1.936	1.860	0.30226	0.89993
Entyloma	MAYO	-0.707	0.675	0.29987	0.89993
Cladosporium	BMI	0.462	0.452	0.31114	0.89993
Aphelidium	Disease_duration	-0.592	0.581	0.31216	0.89993
Debaryomyces	Steroids	-1.384	1.332	0.30284	0.89993
Debaryomyces	biologic_therapy	-1.515	1.434	0.29517	0.89993
Saccharomycetes_gen_Incertae_sedis	Steroids	-0.983	0.945	0.30231	0.89993
Wickerhamomyces	Steroids	-1.040	1.004	0.30436	0.89993
Betamyces	MAYO	1.979	1.814	0.27991	0.89993
Dioszegia	CategorigalAge	1.062	0.981	0.28337	0.89993
Rozellomycota_gen_Incertae_sedis	Steroids	-0.713	0.696	0.31025	0.89993
Candida	MAYO	-1.903	1.890	0.31833	0.90147
Coniochaeta	Disease_duration	-0.127	0.126	0.31597	0.90147
Fungi_gen_Incertae_sedis	immuno_therapy	-0.909	0.976	0.35554	0.90799
Vishniacozyma	Disease_duration	0.561	0.584	0.34086	0.90799
Hyphodontia	Steroids	0.805	0.864	0.35534	0.90799
Cutaneotrichosporon	biologic_therapy	-1.121	1.244	0.37105	0.90799
Cutaneotrichosporon	5-ASA	1.123	1.170	0.34133	0.90799
Malassezia	MAYO	-2.513	2.747	0.36407	0.90799
Entyloma	Disease_duration	-0.146	0.162	0.37171	0.90799
Entyloma	MAYO	-0.605	0.639	0.34776	0.90799
Coniochaeta	biologic_therapy	-0.294	0.296	0.32445	0.90799
Neodevriesiaceae_gen_Incertae_sedis	immuno_therapy	-0.679	0.754	0.37169	0.90799
Aureobasidium	biologic_therapy	-1.079	1.178	0.36330	0.90799
Saccharomycetes_gen_Incertae_sedis	MAYO	1.631	1.708	0.34364	0.90799
Chytridiomycota_gen_Incertae_sedis	MAYO	-1.934	2.094	0.35949	0.90799
Chytridiomycota_gen_Incertae_sedis	MAYO	-2.001	2.215	0.36988	0.90799
Nak5-ASAwaea	BMI	-0.606	0.659	0.36217	0.90799
Meyerozyma	immuno_therapy	-0.627	0.662	0.34725	0.90799
Didymellaceae	Disease_duration	-0.427	0.438	0.33325	0.90799
Exophiala	BMI	0.556	0.603	0.36045	0.90799
Debaryomyces	MAYO	2.266	2.546	0.37713	0.91349
Rhizophydiales_gen_Incertae_sedis	immuno_therapy	-0.539	0.614	0.38331	0.91349
Rhizophydium	BMI	0.224	0.256	0.38427	0.91349
Betamyces	MAYO	1.651	1.888	0.38538	0.91349
Fungi_gen_Incertae_sedis	MAYO	1.252	1.810	0.49209	0.91409
Fungi_gen_Incertae_sedis	MAYO	0.921	1.781	0.60692	0.91409
Fungi_gen_Incertae_sedis	MAYO	-1.086	1.884	0.56649	0.91409
Fungi_gen_Incertae_sedis	biologic_therapy	0.401	1.061	0.70721	0.91409
Fungi_gen_Incertae_sedis	CategorigalAge	0.593	1.384	0.67009	0.91409
Ascomycota	BMI	0.359	0.694	0.60699	0.91409
Ascomycota	Disease_duration	-0.318	0.539	0.55757	0.91409
Ascomycota	MAYO	1.671	2.252	0.46099	0.91409
Ascomycota	Steroids	-0.631	1.178	0.59441	0.91409
Ascomycota	biologic_therapy	-0.993	1.268	0.43689	0.91409
Vishniacozyma	BMI	-0.613	0.752	0.41849	0.91409
Vishniacozyma	MAYO	-1.294	2.307	0.57708	0.91409
Vishniacozyma	MAYO	-1.891	2.439	0.44143	0.91409
Vishniacozyma	Steroids	0.764	1.276	0.55166	0.91409

Vishniacozyma	biologic_therapy	-0.992	1.374	0.47298	0.91409
Hyphodontia	Disease_duration	0.304	0.396	0.44514	0.91409
Hyphodontia	MAYO	-0.816	1.588	0.60946	0.91409
Hyphodontia	immuno_therapy	0.333	0.856	0.69832	0.91409
Hyphodontia	5-ASA	-0.450	0.876	0.60965	0.91409
Hyphodontia	CategorigalAge	-0.559	1.214	0.64701	0.91409
Cutaneotrichosporon	MAYO	0.961	2.088	0.64698	0.91409
Malassezia	BMI	0.425	0.896	0.63697	0.91409
Malassezia	MAYO	-1.473	2.792	0.59966	0.91409
Malassezia	biologic_therapy	0.649	1.636	0.69324	0.91409
Malassezia	CategorigalAge	0.917	2.135	0.66921	0.91409
Clavispora	Disease_duration	0.458	0.541	0.40017	0.91409
Clavispora	MAYO	-1.100	2.257	0.62794	0.91409
Candida	Steroids	0.560	1.029	0.58808	0.91409
Candida	immuno_therapy	-0.449	1.019	0.66116	0.91409
Entyloma	BMI	-0.139	0.208	0.50798	0.91409
Entyloma	MAYO	-0.462	0.649	0.47903	0.91409
Entyloma	immuno_therapy	0.302	0.350	0.39153	0.91409
Entyloma	biologic_therapy	-0.209	0.380	0.58414	0.91409
Entyloma	5-ASA	0.280	0.358	0.43684	0.91409
Entyloma	CategorigalAge	0.391	0.496	0.43402	0.91409
Fusicolla	BMI	0.203	0.435	0.64298	0.91409
Fusicolla	MAYO	0.743	1.333	0.57952	0.91409
Fusicolla	MAYO	0.863	1.409	0.54251	0.91409
Fusicolla	immuno_therapy	0.485	0.730	0.50908	0.91409
Coniochaeta	BMI	0.077	0.162	0.63782	0.91409
Coniochaeta	MAYO	0.266	0.505	0.60077	0.91409
Coniochaeta	Steroids	-0.103	0.275	0.71000	0.91409
Coniochaeta	5-ASA	0.194	0.278	0.48980	0.91409
Botrytis	Disease_duration	-0.232	0.276	0.40465	0.91409
Botrytis	MAYO	0.840	1.108	0.45172	0.91409
Botrytis	immuno_therapy	-0.473	0.597	0.43187	0.91409
Botrytis	biologic_therapy	0.289	0.650	0.65808	0.91409
Botrytis	CategorigalAge	0.406	0.848	0.63335	0.91409
Cladosporium	MAYO	0.904	1.408	0.52324	0.91409
Cladosporium	MAYO	0.519	1.385	0.70926	0.91409
Aureobasidium	MAYO	1.103	1.977	0.57927	0.91409
Aphelidium	BMI	0.549	0.747	0.46515	0.91409
Aphelidium	MAYO	1.128	2.423	0.64334	0.91409
Aphelidium	Steroids	-0.776	1.268	0.54290	0.91409
Debaryomyces	Disease_duration	0.339	0.610	0.58049	0.91409
Debaryomyces	MAYO	1.364	2.447	0.57925	0.91409
Debaryomyces	immuno_therapy	0.522	1.319	0.69358	0.91409
Saccharomycetes	BMI	-0.115	0.276	0.67819	0.91409
Saccharomycetes	Disease_duration	0.162	0.215	0.45201	0.91409
Saccharomycetes	MAYO	-0.492	0.861	0.57000	0.91409
Saccharomycetes	MAYO	-0.488	0.847	0.56658	0.91409
Saccharomycetes	MAYO	0.333	0.895	0.71127	0.91409
Chytridiomycota_gen_Incertae_sedis	BMI	-0.395	0.683	0.56533	0.91409
Chytridiomycota_gen_Incertae_sedis	Disease_duration	0.384	0.531	0.47176	0.91409

Chytridiomycota_gen_Incertae_sedis	MAYO	0.903	2.128	0.67283	0.91409
Chytridiomycota_gen_Incertae_sedis	biologic_therapy	-0.593	1.247	0.63615	0.91409
Nak5-ASAwaea	Disease_duration	-0.220	0.512	0.66893	0.91409
Nak5-ASAwaea	MAYO	1.108	2.055	0.59184	0.91409
Nak5-ASAwaea	MAYO	0.984	2.138	0.64720	0.91409
Nak5-ASAwaea	immuno_therapy	-0.683	1.107	0.53978	0.91409
Nak5-ASAwaea	5-ASA	0.624	1.133	0.58413	0.91409
Nak5-ASAwaea	CategorigalAge	0.628	1.571	0.69080	0.91409
Meyerozyma	BMI	0.155	0.394	0.69642	0.91409
Meyerozyma	MAYO	0.470	1.228	0.70330	0.91409
Meyerozyma	MAYO	1.008	1.208	0.40762	0.91409
Meyerozyma	Steroids	-0.296	0.668	0.65942	0.91409
Meyerozyma	CategorigalAge	0.402	0.939	0.66987	0.91409
Kurtzmaniella	MAYO	0.934	1.955	0.63471	0.91409
Kurtzmaniella	MAYO	1.300	2.035	0.52529	0.91409
Kurtzmaniella	immuno_therapy	-0.758	1.054	0.47478	0.91409
Wickerhamomyces	BMI	0.511	0.592	0.39113	0.91409
Wickerhamomyces	Disease_duration	-0.270	0.460	0.55898	0.91409
Wickerhamomyces	MAYO	0.949	1.844	0.60879	0.91409
Wickerhamomyces	MAYO	0.723	1.815	0.69159	0.91409
Wickerhamomyces	MAYO	1.017	1.919	0.59820	0.91409
Wickerhamomyces	5-ASA	0.551	1.017	0.59019	0.91409
Rhizophydiales_gen_Incertae_sedis	MAYO	0.665	1.139	0.56126	0.91409
Rhizophydiales_gen_Incertae_sedis	MAYO	0.828	1.121	0.46308	0.91409
Rhizophydiales_gen_Incertae_sedis	MAYO	-0.550	1.185	0.64458	0.91409
Rhizophydiales_gen_Incertae_sedis	Steroids	-0.431	0.620	0.48971	0.91409
Alternaria	MAYO	1.139	2.715	0.67652	0.91409
Alternaria	Steroids	-0.588	1.502	0.69700	0.91409
Alternaria	5-ASA	-0.706	1.522	0.64458	0.91409
Didymellaceae	Steroids	-0.635	0.955	0.50870	0.91409
Didymellaceae	immuno_therapy	-0.731	0.946	0.44295	0.91409
Didymellaceae	biologic_therapy	0.805	1.029	0.43713	0.91409
Rhizophydium	MAYO	0.370	0.784	0.63871	0.91409
Rhizophydium	biologic_therapy	-0.221	0.467	0.63728	0.91409
Rhizophydium	CategorigalAge	0.444	0.609	0.46904	0.91409
Betamyces	Disease_duration	-0.207	0.452	0.64869	0.91409
Betamyces	Steroids	-0.671	0.988	0.49944	0.91409
Betamyces	5-ASA	0.820	1.001	0.41608	0.91409
Dioszegia	MAYO	-0.705	1.282	0.58479	0.91409
Dioszegia	MAYO	-0.878	1.334	0.51318	0.91409
Dioszegia	immuno_therapy	0.461	0.691	0.50700	0.91409
Dioszegia	5-ASA	-0.431	0.707	0.54438	0.91409
Rozellomycota_gen_Incertae_sedis	MAYO	0.881	1.259	0.48702	0.91409
Rozellomycota_gen_Incertae_sedis	immuno_therapy	-0.573	0.690	0.40910	0.91409
Rozellomycota_gen_Incertae_sedis	CategorigalAge	0.806	0.978	0.41320	0.91409
Exophiala	MAYO	1.010	1.880	0.59329	0.91409
Exophiala	MAYO	1.006	1.850	0.58886	0.91409
Exophiala	MAYO	1.587	1.957	0.42050	0.91409
Exophiala	Steroids	-0.593	1.023	0.56450	0.91409
Exophiala	immuno_therapy	0.621	1.014	0.54269	0.91409

Malassezia	Steroids	-0.553	1.519	0.71704	0.91493
Meyerozyma	Disease_duration	0.110	0.306	0.72051	0.91493
Kurtzmaniella	MAYO	0.693	1.924	0.71983	0.91493
Hyphodontia	BMI	0.177	0.510	0.73003	0.92314
Neodevriesiaceae_gen_Incertae_sedis	Disease_duration	-0.120	0.349	0.73274	0.92314
Ascomycota	MAYO	0.638	2.164	0.76898	0.92391
Candida	MAYO	-0.614	1.967	0.75622	0.92391
Candida	5-ASA	0.347	1.043	0.74039	0.92391
Coniochaeta	MAYO	-0.145	0.497	0.77089	0.92391
Cladosporium	biologic_therapy	0.255	0.825	0.75806	0.92391
Saccharomycetes_gen_Incertae_sedis	biologic_therapy	-0.311	1.018	0.76093	0.92391
Kurtzmaniella	BMI	0.186	0.627	0.76831	0.92391
Wickerhamomyces	immuno_therapy	-0.316	0.994	0.75158	0.92391
Rhizophydiales_gen_Incertae_sedis	CategorigalAge	0.285	0.871	0.74429	0.92391
Rhizophydium	MAYO	0.252	0.829	0.76189	0.92391
Dioszegia	MAYO	0.397	1.262	0.75409	0.92391
Rozellomycota_gen_Incertae_sedis	MAYO	0.393	1.331	0.76882	0.92391
Exophiala	Disease_duration	-0.154	0.469	0.74306	0.92391
Botrytis	5-ASA	-0.175	0.611	0.77555	0.92537
Debaryomyces	BMI	-0.223	0.785	0.77789	0.92537
Fungi_gen_Incertae_sedis	5-ASA	0.247	0.998	0.80549	0.92941
Clavispora	MAYO	-0.576	2.134	0.78832	0.92941
Fusicolla	MAYO	0.333	1.354	0.80678	0.92941
Aphelidium	MAYO	0.570	2.329	0.80743	0.92941
Debaryomyces	MAYO	0.609	2.407	0.80119	0.92941
Kurtzmaniella	Disease_duration	0.121	0.487	0.80529	0.92941
Alternaria	Disease_duration	-0.183	0.688	0.79101	0.92941
Rhizophydium	MAYO	0.197	0.797	0.80590	0.92941
Dioszegia	biologic_therapy	-0.195	0.752	0.79580	0.92941
Clavispora	MAYO	-0.523	2.169	0.81033	0.92942
Rozellomycota_gen_Incertae_sedis	MAYO	0.302	1.279	0.81434	0.93068
Didymellaceae	MAYO	0.402	1.827	0.82658	0.94130
Hyphodontia	biologic_therapy	-0.199	0.931	0.83171	0.94290
Neodevriesiaceae_gen_Incertae_sedis	MAYO	-0.295	1.399	0.83388	0.94290
Rozellomycota_gen_Incertae_sedis	biologic_therapy	0.150	0.750	0.84189	0.94861
Fungi_gen_Incertae_sedis	Steroids	0.189	0.985	0.84867	0.94956
Botrytis	MAYO	0.223	1.153	0.84704	0.94956
Malassezia	immuno_therapy	0.238	1.505	0.87512	0.95071
Cladosporium	MAYO	0.232	1.465	0.87474	0.95071
Cladosporium	immuno_therapy	-0.119	0.759	0.87644	0.95071
Kurtzmaniella	Steroids	-0.184	1.064	0.86353	0.95071
Rhizophydiales_gen_Incertae_sedis	biologic_therapy	0.109	0.667	0.87123	0.95071
Didymellaceae	BMI	0.094	0.563	0.86803	0.95071
Rhizophydium	5-ASA	-0.069	0.439	0.87574	0.95071
Betamyces	biologic_therapy	0.198	1.063	0.85282	0.95071
Dioszegia	BMI	0.075	0.412	0.85595	0.95071
Vishniacozyma	MAYO	-0.287	2.344	0.90292	0.97613
Betamyces	MAYO	0.194	1.785	0.91378	0.98454
Didymellaceae	CategorigalAge	0.140	1.342	0.91716	0.98487
Cutaneotrichosporon	MAYO	0.200	2.208	0.92824	0.99264

Cutaneotrichosporon	immuno_therapy	-0.100	1.144	0.93089	0.99264
Candida	CategorigalAge	0.121	1.446	0.93370	0.99264
Clavispora	Steroids	-0.056	1.181	0.96239	0.99310
Entyloma	Steroids	0.017	0.353	0.96237	0.99310
Coniochaeta	MAYO	0.024	0.525	0.96369	0.99310
Botrytis	MAYO	0.038	1.091	0.97197	0.99310
Saccharomycetes_gen_Incertae_sedis	MAYO	0.127	1.736	0.94195	0.99310
Saccharomycetes	biologic_therapy	0.024	0.504	0.96200	0.99310
Saccharomycetes	CategorigalAge	0.037	0.658	0.95544	0.99310
Chytridiomycota_gen_Incertae_sedis	Steroids	-0.080	1.158	0.94510	0.99310
Nak5-ASAwaea	MAYO	-0.065	2.022	0.97448	0.99310
Nak5-ASAwaea	biologic_therapy	-0.050	1.204	0.96688	0.99310
Meyerozyma	MAYO	0.051	1.278	0.96850	0.99310
Didymellaceae	MAYO	-0.070	1.755	0.96830	0.99310
Didymellaceae	5-ASA	0.051	0.968	0.95828	0.99310
Meyerozyma	5-ASA	-0.017	0.677	0.97993	0.99549
Ascomycota	MAYO	-0.017	2.129	0.99378	0.99670
Aureobasidium	Disease_duration	0.010	0.501	0.98493	0.99670
Aphelidium	MAYO	0.020	2.291	0.99290	0.99670
Saccharomycetes	Steroids	0.003	0.468	0.99438	0.99670
Alternaria	MAYO	-0.012	2.872	0.99670	0.99670
