# Supplementary material for: Ecological patterns of the gut mycobiome and microbiome in ulcerative colitis across life stages
Source: Front Cell Infect Microbiol. 2026 Apr 28;16:1769892. doi: 10.3389/fcimb.2026.1769892 (PMC13161133; doi:10.3389/fcimb.2026.1769892)
Supplement: Supplementary file 4 [file DataSheet4.pdf]

Table S3. Age-group comparisons after stratification by disease activity. Mann–Whitney U test p-values comparing paediatric and adult ulcerative colitis (UC) patients within matched disease activity categories (Mayo endoscopic score). Significant results ($p < 0.05$) identify taxa showing age-associated differences within comparable disease severity stratification.

**Taxon**	**Mild activity** p value	**Moderate activity** p value	**Severe activity** p value
Aureobasidium	0.004	0.015	0.023
Clavispora	0.004	<0.001	<0.001
Cutaneotrichosporon	0.03	0.015	0.026
Vishniacozyma	0.03	0.06	0.064
CAG508	0.03	0.179	0.6
Cladosporium	0.052	0.015	0.001
Chytridiomycota gen. incertae sedis	0.082	0.001	—
Saccharomycetes gen. incertae sedis	0.247	0.042	0.099
Phocaeicola A858004	0.329	0.019	0.672
Romboutsia B	0.329	0.464	0.032
Ruminococcus E	0.429	0.009	0.11
Gemmiger A73129	0.662	0.006	0.135
